# Supplementary material for: Synthesis of Carbonyl-Containing Oxindoles via Ni-Catalyzed Reductive Aryl-Acylation and Aryl-Esterification of Alkenes
Source: Molecules. 2022 Sep 11;27(18):5899. doi: 10.3390/molecules27185899 (PMC9503384; doi:10.3390/molecules27185899)
Supplement: Supplementary file 1 [file molecules-27-05899-s001.zip › molecules-1881755-supplementary.pdf]

Supporting information

Synthesis of Carbonyl-Containing Oxindoles via Ni-Catalyzed Reductive Aryl-Acylation and Aryl-Esterification of Alkenes

Zhengtian Ding, Wangqing Kong*

The Institute for Advanced Studies (IAS), Wuhan University, Wuhan, 430072, P. R. China

E-mail: wqkong@whu.edu.cn

Table of Contents

1. General Information S3

2. General procedure S4

3. Optimization of reaction conditions S5

4. Control experiments S6

5. Characterization data of products S7

6. Copies of the ^{1}H, ^{19}F NMR and ^{13}C NMR spectra S32

7. References S77

1. General information

^{1}H and ^{13}C NMR data were recorded with Bruker ADVANCE III (600 MHz) or JNM-ECZ400S/L1 (400 MHz) spectrometers. Chemical shifts are given in ppm. The spectra are calibrated to the residual ^{1}H and ^{13}C signals of the solvents. Multiplicities are abbreviated as follows: singlet (s), doublet (d), triplet (t), quartet (q), doublet-doublet (dd), quintet (quint), septet (sept), multiplet (m), and broad (b). ^{19}F NMR spectra were recorded using $CFCl_3$ as internal standard. Gas chromatography were determined with a SHIMADZU Nexis GC 2030 gas chromatography instrument with an FID detector. High-resolution mass spectra (HRMS) were recorded on Thermo Fisher Orbitrap Elite mass spectrometer. Column and elution details were specified in each entry.

Unless otherwise stated, starting materials were purchased from commercial suppliers (Energy Chemical, Alfa, Aldrich and so on). All reactions dealing with air- or moisture-sensitive compounds were performed in the argon-filled glove box or by standard Schlenk techniques in oven-dried reaction vessels under argon atmosphere. Solvents were purchased in HPLC quality, degassed by purging thoroughly with argon and dried over activated molecular sieves of appropriate size. More sensitive compounds were stored in a desiccator or in a glove-box if required. Reactions were monitored by thin layer chromatography (TLC) using glass 0.25 mm silica gel plates. Compounds were visualized by UV-light at 254 nm and by dipping the plates in an aqueous potassium permanganate solution followed by heating. Flash column chromatography was performed over silica gel (200-400 mesh).

2. General procedure

2.1 General Procedure for the synthesis of ketones

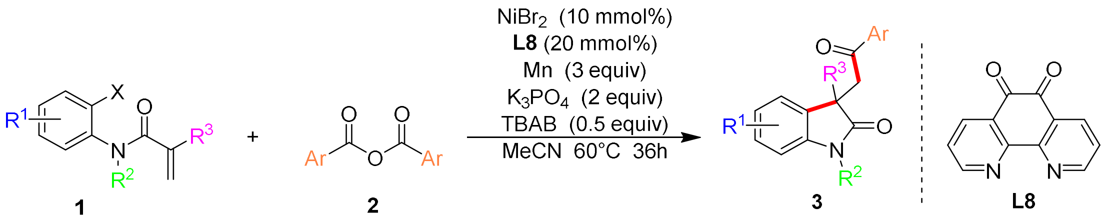

An oven-dried sealed tube equipped with a PTFE-coated stir bar was charged with $NiBr_2$ (10 mol%), 1,10-phenanthroline-5,6-dione (**L8**) (20 mol%), **1** (0.1 mmol, 1.0 equiv), manganese powder (3.0 equiv), TBAB (0.5 equiv) and K_3PO_4 (2.0 equiv). The sealed tube was evacuated and backfilled with argon (this process was repeated for three times) and then MeCN (0.05 M) was added. This reaction mixture was stirred at room temperature for 15 minutes and then aryl anhydride **2** (2.0 equiv) was added. The reaction was heated at 60 ºC until the reaction was complete (monitored by TLC). The resulting mixture was purified by chromatography on silica gel, eluting with ethyl acetate/petroleum ether 1:20~1:5 (v/v) to afford the corresponding products **3**.

2.2 General Procedure for the synthesis of esters

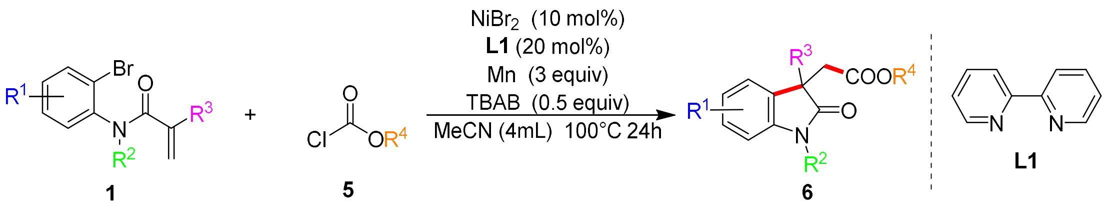

An oven-dried sealed tube equipped with a PTFE-coated stir bar was charged with $NiBr_2$ (10 mol%), bpy (**L1**) (20 mol%), **1** (0.1 mmol, 1.0 equiv), manganese powder (3.0 equiv) and TBAB (0.5 equiv). The sealed tube was evacuated and backfilled with argon (this process was repeated for three times) and then MeCN (0.025 M) was added. This reaction mixture was stirred at room temperature for 15 minutes and then acid chloride **5** (2.0~4.0 equiv) was added. Then, the reaction heated at 100 ºC until the reaction was complete (monitored by TLC). The resulting mixture was purified by chromatography on silica gel, eluting with ethyl acetate/petroleum ether 1:20~1:5 (v/v) to afford the corresponding products **6**.

3. Optimization of reaction conditions

Table S1: Optimization reaction conditions for the synthesis of esters

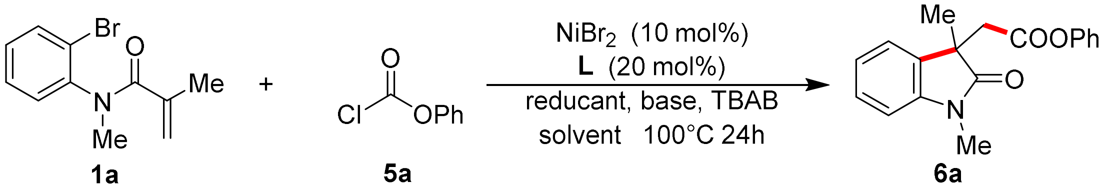

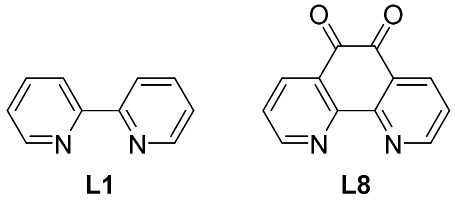

entry	L	reductant	solvent	base	yield of **6a** (%)[b]
1	**L8**	Mn	MeCN	K_3PO_4	51
2	**L8**	Mn	DMF	K_3PO_4	<1
3	**L8**	Mn	DMAc	K_3PO_4	<1
4	**L8**	Zn	MeCN	K_3PO_4	17
5	**L8**	Mn	MeCN	K_3CO_4	42
6	**L8**	Mn	MeCN	Cs_2CO_3	52
7	**L8**	Mn	MeCN	-	53
8[c]	**L8**	Mn	MeCN	-	58
9[c]	**L1**	Mn	MeCN	-	75
10[c,d]	**L1**	Mn	MeCN	-	51
11[d,e]	**L1**	Mn	MeCN	-	55

[a] Unless indicated otherwise, reactions of **1a** (0.10 mmol), **5a** (0.40 mmol), $NiBr_2$ (0.01 mmol), ligand (0.02 mmol), reductant (0.30 mmol), TBAB (0.05 mmol), and K_3PO_4 (0.20 mmol) were carried out in solvent (2 mL) at 100 °C for 24h. [b]Isolated yields. [c]4mL MeCN was used. [d]80 °C. [e]Without TBAB.

4. Control experiments

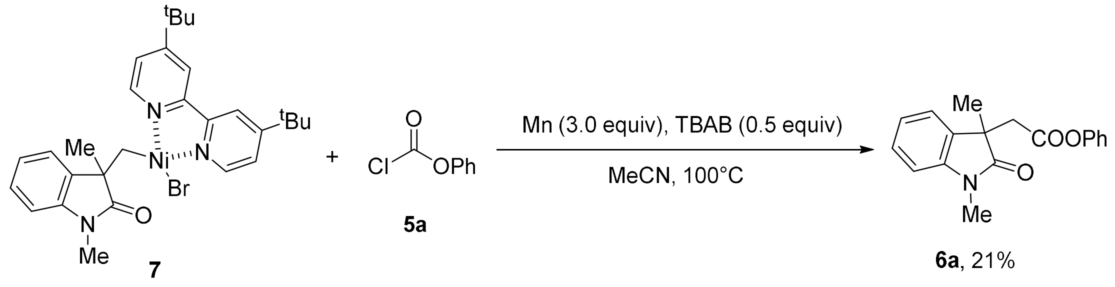

Experimental procedure[1]: An oven-dried seal-tube equipped with a PTFE-coated stir bar was charged with TBAB (0.025 mmol, 8.1 mg), Mn^0 (0.15 mmol, 9.8 mg), complex **7** (0.05 mmol, 29.1 mg) and phenyl carbonochloridate (0.1 mmol, 15.7 mg) in anhydrous MeCN (1 mL). The seal-tube was sealed and removed from the glovebox. Then the reaction was stirred at 100 °C for overnight. The resulting mixture was purified by chromatography on silica gel, eluting with ethyl acetate/petroleum ether 1:10 (v/v) to afford the corresponding products **6a** (3.1 mg, 21% yield).

5. Characterization data of products

1,3-dimethyl-3-(2-oxo-2-phenylethyl)indolin-2-one (3a)

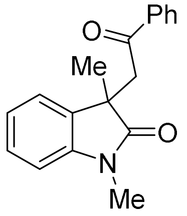

Chemical Formula: $C_{18}H_{17}NO_2$
Exact Mass: 279.1259

3a was prepared according to general procedure **2.1** using *N*-(2-bromophenyl)-*N*-methylmethacrylamide (0.1 mmol, 25.4 mg) and benzoic anhydride (0.2 mmol, 45.2 mg) and was purified by silica gel column chromatography (petroleum ether/ethyl acetate =10/1~5/1) to obtain **3a** as yellow oil (23.7 mg, 85% yield). TLC (petroleum ether: ethyl acetate, 5:1 v/v): Rf = 0.4. The ^{1}H NMR data matched those reported in the literature.[2]

^{1}H NMR (400 MHz, $CDCl_3$) δ 7.87-7.80 (m, 2H), 7.56-7.48 (m, 1H), 7.45-7.36 (m, 2H), 7.28-7.22 (m, 1H), 7.16-7.12 (m, 1H), 7.01-6.95 (m, 1H), 6.90 (d, *J* = 7.8 Hz, 1H), 3.72 (d, *J* = 17.9 Hz, 1H), 3.65 (d, *J* = 17.8 Hz, 1H), 3.31 (s, 3H), 1.44 (s, 3H).

^{13}C NMR (101 MHz, $CDCl_3$) δ 196.1, 180.6, 143.8, 136.4, 133.7, 133.2, 128.5, 128.0, 127.8, 122.2, 121.8, 108.2, 46.0, 45.3, 26.5, 24.9.

5-methoxy-1,3-dimethyl-3-(2-oxo-2-phenylethyl)indolin-2-one (3b)

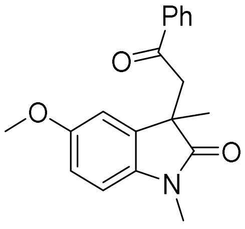

Chemical Formula: $C_{19}H_{19}NO_3$
Exact Mass: 309.14

3b was prepared according to general procedure **2.1** using *N*-(2-bromo-4-methoxyphenyl)-*N*-methylmethacrylamide (0.1 mmol, 28.4 mg) and benzoic anhydride (0.2 mmol, 45.2 mg) and was purified by silica gel column chromatography (petroleum ether/ethyl acetate = 5/1) to obtain **3b** as brown oil (22.9 mg, 74% yield). TLC (petroleum ether: ethyl acetate, 5:1 v/v): Rf

= 0.3. The ^{1}H NMR data matched those reported in the literature.[2]

^{1}H NMR (400 MHz, CDCl$_3$) δ 7.91-7.79 (m, 2H), 7.57-7.46 (m, 1H), 7.46-7.32 (m, 2H), 6.86-6.79 (m, 1H), 6.79-6.71 (m, 2H), 3.73 (s, 3H), 3.67 (s, 2H), 3.29 (s, 3H), 1.43 (s, 3H).

^{13}C NMR (101 MHz, CDCl$_3$) δ 196.0, 155.6, 137.4, 136.2, 135.1, 133.2, 128.4, 127.9, 111.3, 109.8, 108.3, 55.6, 45.9, 45.6, 26.5, 25.0.

1,3,5-trimethyl-3-(2-oxo-2-phenylethyl)indolin-2-one (3c)

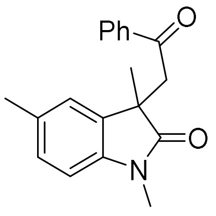

Chemical Formula: $C_{19}H_{19}NO_2$
Exact Mass: 293.1416

3c was prepared according to general procedure **2.1** using *N*-(2-bromo-4-methylphenyl)-*N*-methylmethacrylamide (0.1 mmol, 26.8 mg) and benzoic anhydride (0.2 mmol, 45.2 mg) and was purified by silica gel column chromatography (petroleum ether/ethyl acetate =5/1) to obtain **3c** as yellow oil (23.7 mg, 81% yield). TLC (petroleum ether: ethyl acetate, 5:1 v/v): Rf = 0.4. The ^{1}H NMR data matched those reported in the literature.[3]

^{1}H NMR (400 MHz, CDCl$_3$) δ 7.90-7.81 (m, 2H), 7.56-7.48 (m, 1H), 7.44-7.36 (m, 2H), 7.09-7.02 (m, 1H), 6.97-6.92 (m, 1H), 6.79 (d, J = 7.9 Hz, 1H), 3.75-3.59 (m, 2H), 3.29 (s, 3H), 2.27 (s, 3H), 1.43 (s, 3H).

^{13}C NMR (101 MHz, CDCl$_3$) δ 196.1, 180.5, 141.4, 136.4, 133.7, 133.1, 131.6, 128.5, 128.1, 128.0, 122.7, 107.8, 46.0, 45.3, 26.5, 25.0, 21.1.

5-fluoro-1,3-dimethyl-3-(2-oxo-2-phenylethyl)indolin-2-one (3d)

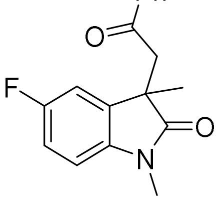

Chemical Formula: $C_{18}H_{16}FNO_2$
Exact Mass: 297.12

3d was prepared according to general procedure **2.1** using *N*-(2-bromo-4-fluorophenyl)-*N*-methylmethacrylamide (0.1 mmol, 27.2 mg) and benzoic anhydride (0.2 mmol, 45.2 mg) and was purified by silica gel column chromatography (petroleum ether/ethyl acetate =10:1~5/1) to obtain **3d** as yellow oil (26.7 mg, 90% yield). TLC (petroleum ether: ethyl acetate, 5:1 v/v): Rf = 0.4. The ^{1}H NMR data matched those reported in the literature.[4]

^{1}H NMR (400 MHz, $CDCl_3$) δ 7.89-7.79 (m, 2H), 7.58-7.49 (m, 1H), 7.46-7.37 (m, 2H), 7.02-6.92 (m, 1H), 6.90 (dd, *J* = 8.0, 2.5 Hz, 1H), 6.82 (dd, *J* = 8.5, 4.2 Hz, 1H), 3.67 (s, 2H), 3.30 (s, 3H), 1.44 (s, 3H).

^{13}C NMR (101 MHz, $CDCl_3$) δ 195.9, 180.2, 157.9, 139.8, 136.1, 135.4 (d, *J* = 7.8 Hz), 133.3, 128.6, 127.9, 113.8 (d, *J* = 23.3 Hz), 110.1 (d, *J* = 24.9 Hz), 108.5 (d, *J* = 8.2 Hz), 46.0, 45.7, 26.6, 24.8.

^{19}F NMR (377 MHz, $CDCl_3$) δ -121.1 (s).

1,3-dimethyl-3-(2-oxo-2-phenylethyl)-5-(trifluoromethyl)indolin-2-one (3e)

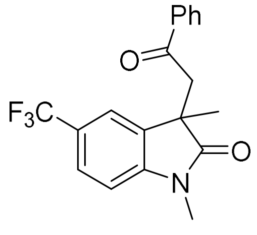

Chemical Formula: $C_{19}H_{16}F_3NO_2$
Exact Mass: 347.11

3e was prepared according to general procedure **2.1** using *N*-(2-bromo-4-(trifluoromethyl)phenyl)-*N*-methylmethacrylamide (0.1 mmol, 32.2 mg) and benzoic anhydride (0.2 mmol, 45.2 mg) and was purified by silica gel column chromatography (petroleum ether/ethyl acetate =10:1~5/1) to obtain **3e** as yellow solid (18.0 mg, 52% yield). TLC (petroleum ether: ethyl acetate, 5:1 v/v): Rf = 0.4. m.p. 114.5-115.9 °C. The ^{1}H NMR data matched those reported in the literature.[5]

^{1}H NMR (400 MHz, $CDCl_3$) δ 7.90-7.80 (m, 2H), 7.59-7.50 (m, 2H), 7.46-7.38 (m, 2H), 7.34 (d, *J* = 1.8 Hz, 1H), 6.98 (d, *J* = 8.2 Hz, 1H), 3.75 (s, 2H), 3.36 (s, 3H), 1.45 (s, 3H).

^{13}C NMR (101 MHz, $CDCl_3$) δ 195.8, 180.5, 146.9, 135.8, 134.4, 133.5, 128.6, 127.9, 125.7 (d, *J* = 4.0 Hz), 118.5, 107.9, 46.1, 45.1, 26.7, 24.9.

^{19}F NMR (377 MHz, $CDCl_3$) δ -61.2 (s).

6-methoxy-1,3-dimethyl-3-(2-oxo-2-phenylethyl)indolin-2-one (3f)

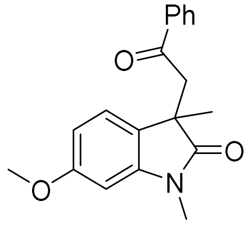

Chemical Formula: $C_{19}H_{19}NO_3$
Exact Mass: 309.14

3f was prepared according to general procedure **2.1** using *N*-(2-bromo-5-methoxyphenyl)-*N*-methylmethacrylamide (0.1 mmol, 28.4 mg) and benzoic anhydride (0.2 mmol, 45.2 mg) and was purified by silica gel column chromatography (petroleum ether/ethyl acetate =10:1~5/1) to obtain **3f** as yellow oil (19.5 mg, 63% yield). TLC (petroleum ether: ethyl acetate, 5:1 v/v): Rf = 0.3. The ^1H NMR data matched those reported in the literature.[5]

^1H NMR (400 MHz, $CDCl_3$) δ 7.88-7.80 (m, 2H), 7.54-7.48 (m, 1H), 7.43-7.36 (m, 2H), 7.03 (d, J = 8.0 Hz, 1H), 6.52-6.44 (m, 2H), 3.80 (s, 3H), 3.69 (d, J = 17.9 Hz, 1H), 3.60 (d, J = 17.9 Hz, 1H), 3.29 (s, 3H).

^{13}C NMR (101 MHz, $CDCl_3$) δ 196.3, 181.1, 159.9, 145.0, 136.3, 133.1, 128.4, 127.9, 125.6, 122.3, 105.8, 96.2, 55.4, 46.0, 44.8, 26.4, 25.0.

6-chloro-1,3-dimethyl-3-(2-oxo-2-phenylethyl)indolin-2-one (3g)

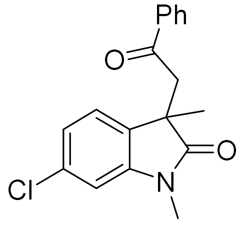

Chemical Formula: $C_{18}H_{16}ClNO_2$
Exact Mass: 313.09

3g was prepared according to general procedure **2.1** using *N*-(2-bromo-5-chlorophenyl)-*N*-methylmethacrylamide (0.1 mmol, 28.9 mg) and benzoic anhydride (0.2 mmol, 45.2 mg) and was purified by silica gel column chromatography (petroleum ether/ethyl acetate =10:1~5/1) to obtain **3g** as white solid (24.4 mg, 78% yield). TLC (petroleum ether: ethyl acetate, 5:1 v/v):

Rf = 0.4. m.p. 142.1-143.4 °C. The ^1H NMR data matched those reported in the literature.[8]

^1H NMR (400 MHz, $CDCl_3$) δ 7.88-7.79 (m, 2H), 7.57-7.48 (m, 1H), 7.46-7.35 (m, 2H), 7.03 (d, *J* = 7.8 Hz, 1H), 6.94 (dd, *J* = 7.8, 1.8 Hz, 1H), 6.90 (d, *J* = 1.8 Hz, 1H), 3.69 (dd, *J* = 21.7, 18.1 Hz, 2H), 3.30 (s, 3H), 1.42 (s, 3H).

^{13}C NMR (101 MHz, $CDCl_3$) δ 195.9, 180.5, 145.0, 136.0, 133.5, 133.3, 132.1, 128.5, 127.9, 122.5, 121.9, 108.9, 46.0, 44.9, 26.5, 24.8.

1,3,6-trimethyl-3-(2-oxo-2-phenylethyl)indolin-2-one (3h)

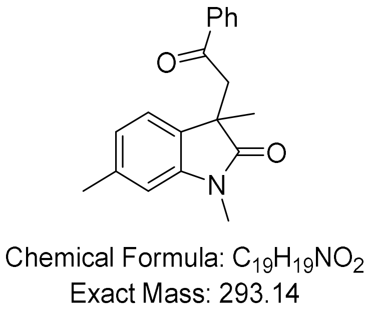

3h was prepared according to general procedure **2.1** using *N*-(2-bromo-5-methylphenyl)-*N*-methylmethacrylamide (0.1 mmol, 26.8 mg) and benzoic anhydride (0.2 mmol, 45.2 mg) and was purified by silica gel column chromatography (petroleum ether/ethyl acetate =10:1~5/1) to obtain **3h** as colorless oil (24.3 mg, 83% yield). TLC (petroleum ether: ethyl acetate, 5:1 v/v): Rf = 0.4. The ^1H NMR data matched those reported in the literature.[4]

^1H NMR (400 MHz, $CDCl_3$) δ 7.91-7.78 (m, 2H), 7.58-7.46 (m, 1H), 7.45-7.34 (m, 2H), 7.02 (d, *J* = 7.5 Hz, 1H), 6.81-6.76 (m, 1H), 6.73 (s, 1H), 3.65 (dd, *J* = 29.9, 17.9 Hz, 2H), 3.29 (s, 3H), 2.37 (s, 3H), 1.42 (s, 3H).

^{13}C NMR (101 MHz, $CDCl_3$) δ 196.2, 180.9, 143.9, 137.8, 136.4, 133.1, 130.8, 128.4, 128.0, 122.6, 121.5, 109.2, 46.0, 45.1, 26.4, 25.0, 21.8.

1,3,7-trimethyl-3-(2-oxo-2-phenylethyl)indolin-2-one (3i)

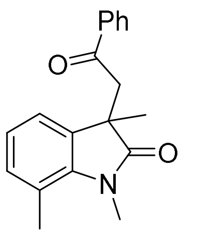

Chemical Formula: $C_{19}H_{19}NO_2$
Exact Mass: 293.14

3i was prepared according to general procedure **2.1** using *N*-(2-bromo-6-methylphenyl)-*N*-methylmethacrylamide (0.1 mmol, 26.8 mg) and benzoic anhydride (0.2 mmol, 45.2 mg) and was purified by silica gel column chromatography (petroleum ether/ethyl acetate =10:1~5/1) to obtain **3i** as brown oil (14.9 mg, 51% yield). TLC (petroleum ether: ethyl acetate, 5:1 v/v): Rf = 0.4. The ^{1}H NMR data matched those reported in the literature.[4]

^{1}H NMR (400 MHz, $CDCl_3$) δ 7.92-7.78 (m, 2H), 7.58-7.47 (m, 1H), 7.47-7.33 (m, 2H), 7.02-6.90 (m, 2H), 6.89-6.81 (m, 1H), 3.67 (d, *J* = 0.8 Hz, 2H), 3.59 (s, 3H), 2.62 (s, 3H), 1.41 (s, 3H).

^{13}C NMR (101 MHz, $CDCl_3$) δ 196.2, 181.4, 141.6, 136.4, 134.3, 133.1, 131.6, 128.4, 128.0, 122.1, 119.8, 119.5, 46.3, 44.7, 29.8, 25.5, 19.1.

1-benzyl-3-methyl-3-(2-oxo-2-phenylethyl)indolin-2-one (3j)

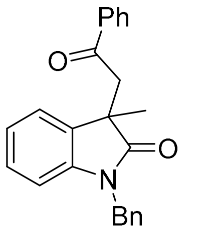

Chemical Formula: $C_{24}H_{21}NO_2$
Exact Mass: 355.16

3j was prepared according to general procedure **2.1** using *N*-benzyl-*N*-(2-bromophenyl)methacrylamide (0.1 mmol, 33.0 mg) and benzoic anhydride (0.2 mmol, 45.2 mg) and was purified by silica gel column chromatography (petroleum ether/ethyl acetate = 5/1) to obtain **3j** as yellow solid (30.2 mg, 85% yield). TLC (petroleum ether: ethyl acetate, 5:1 v/v): Rf = 0.4. m.p. 146.4-148.8 °C. The ^{1}H NMR data matched those reported in the literature.[5]

^{1}H NMR (400 MHz, $CDCl_3$) δ 7.94-7.85 (m, 2H), 7.58-7.49 (m, 1H), 7.48-7.39 (m, 4H), 7.39-7.32 (m, 2H), 7.32-7.26 (m, 1H), 7.18-7.08 (m, 2H), 6.99-6.90 (m, 1H), 6.78-6.71 (m, 1H), 5.10

(d, J = 15.8 Hz, 1H), 4.98 (d, J = 15.8 Hz, 1H), 3.75 (dd, J = 23.3, 18.0 Hz, 2H), 1.51 (s, 3H). ^{13}C NMR (101 MHz, $CDCl_3$) δ 195.9, 180.7, 142.9, 136.31, 136.28, 133.8, 133.3, 128.8, 128.5, 128.1, 127.7, 127.4, 127.3, 122.2, 121.7, 109.3, 45.9, 45.4, 44.0, 25.6.

3-(methoxymethyl)-1-methyl-3-(2-oxo-2-phenylethyl)indolin-2-one (3k)

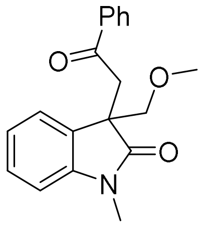

Chemical Formula: $C_{19}H_{19}NO_3$
Exact Mass: 309.14

3k was prepared according to general procedure **2.1** using *N*-(2-bromophenyl)-2-(methoxymethyl)-*N*-methylacrylamide (0.1 mmol, 28.4 mg) and benzoic anhydride (0.2 mmol, 45.2 mg) and was purified by silica gel column chromatography (petroleum ether/ethyl acetate = 5/1) to obtain **3k** as yellow solid (23.8 mg, 77% yield). TLC (petroleum ether: ethyl acetate, 5:1 v/v): Rf = 0.3. m.p. 126.3-128.6 °C. The ^{1}H NMR data matched those reported in the literature.[5]

^{1}H NMR (400 MHz, $CDCl_3$) δ 7.92-7.79 (m, 2H), 7.56-7.46 (m, 1H), 7.45-7.34 (m, 2H), 7.32-7.21 (m, 2H), 7.04-6.94 (m, 1H), 6.90 (d, J = 7.7 Hz, 1H), 3.96 (d, J = 18.1 Hz, 1H), 3.74 (d, J = 8.8 Hz, 1H), 3.64 (d, J = 18.1 Hz, 1H), 3.44 (d, J = 8.7 Hz, 1H), 3.31 (s, 3H), 3.30 (s, 3H). ^{13}C NMR (101 MHz, $CDCl_3$) δ 196.0, 177.9, 144.4, 136.3, 133.1, 130.8, 128.4, 128.1, 128.0, 123.1, 122.0, 108.0, 59.6, 50.5, 42.2, 29.7, 26.5.

3-benzyl-1-methyl-3-(2-oxo-2-phenylethyl)indolin-2-one (3l)

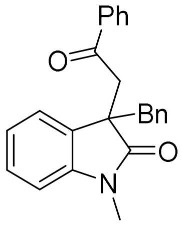

Chemical Formula: $C_{24}H_{21}NO_2$
Exact Mass: 355.16

3l was prepared according to general procedure **2.1** using 2-benzyl-*N*-(2-bromophenyl)-*N*-

methylacrylamide (0.1 mmol, 33.0 mg) and benzoic anhydride (0.2 mmol, 45.2 mg) and was purified by silica gel column chromatography (petroleum ether/ethyl acetate = 5/1) to obtain **3l** as yellow solid (21.3 mg, 60% yield). TLC (petroleum ether: ethyl acetate, 5:1 v/v): Rf = 0.4. m.p. 142.6-144.7 °C. The ^{1}H NMR data matched those reported in the literature.[5]

^{1}H NMR (400 MHz, $CDCl_3$) δ 7.88-7.82 (m, 2H), 7.57-7.49 (m, 1H), 7.44-7.37 (m, 2H), 7.18 (td, *J* = 7.7, 1.3 Hz, 1H), 7.14-7.01 (m, 44H), 6.95 (td, *J* = 7.5, 1.0 Hz, 1H), 6.88-6.80 (m, 2H), 6.66-6.60 (m, 1H), 3.81 (d, *J* = 2.7 Hz, 2H), 3.15 (d, *J* = 12.7 Hz, 1H), 3.09 (d, *J* = 12.7 Hz, 1H), 3.04 (s, 3H).

^{13}C NMR (101 MHz, $CDCl_3$) δ 195.8, 179.1, 144.3, 136.4, 134.8, 133.2, 130.9, 130.0, 128.5, 128.0, 127.9, 127.4, 126.7, 122.7, 121.7, 107.8, 50.9, 44.9, 44.5, 26.0.

3-hexyl-1-methyl-3-(2-oxo-2-phenylethyl)indolin-2-one (3m)

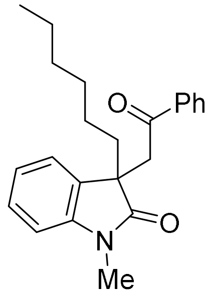

Chemical Formula: $C_{23}H_{27}NO_2$
Exact Mass: 349.2042

3m was prepared according to general procedure **2.1** using *N*-(2-bromophenyl)-*N*-methyl-2-methyleneoctanamide (0.1 mmol, 32.4 mg) and benzoic anhydride (0.2 mmol, 45.2 mg) and was purified by silica gel column chromatography (petroleum ether/ethyl acetate =10:1~5/1) to obtain **3m** as yellow oil (20.9 mg, 60% yield). TLC (petroleum ether: ethyl acetate, 5:1 v/v): Rf = 0.4.

^{1}H NMR (400 MHz, $CDCl_3$) δ 7.85-7.78 (m, 2H), 7.54-7.47 (m, 1H), 7.42-7.35 (m, 2H), 7.28-7.22 (m, 1H), 7.12-7.07 (m, 1H), 7.00-6.94 (m, 1H), 6.87 (d, *J* = 7.6 Hz, 1H), 3.72 (d, *J* = 17.8 Hz, 1H), 3.62 (d, *J* = 17.8 Hz, 1H), 3.29 (s, 3H), 1.95-1.79 (m, 2H), 1.24-0.98 (m, 8H), 0.85-0.77 (m, 3H).

^{13}C NMR (101 MHz, $CDCl_3$) δ 196.2, 180.1, 144.6, 136.5, 133.1, 132.1, 128.4, 127.9, 127.8, 122.03, 121.99, 107.9, 49.4, 45.8, 38.7, 31.5, 29.3, 26.3, 23.2, 22.5, 14.0.

HRMS: (ESI) calcd for $C_{23}H_{27}NO_2H^+[M+H]^+$ 350.2115; found 350.2106.

3-isopropyl-1-methyl-3-(2-oxo-2-phenylethyl)indolin-2-one (3n)

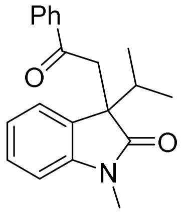

Chemical Formula: $C_{20}H_{21}NO_2$
Exact Mass: 307.1572

3n was prepared according to general procedure **2.1** using *N*-(2-bromophenyl)-*N*,3-dimethyl-2-methylenebutanamide (0.1 mmol, 28.2 mg) and benzoic anhydride (0.2 mmol, 45.2 mg) and was purified by silica gel column chromatography (petroleum ether/ethyl acetate =10:1~5/1) to obtain **3n** as colorless oil (19.0 mg, 62% yield). TLC (petroleum ether: ethyl acetate, 5:1 v/v): Rf = 0.4.

^{1}H NMR (400 MHz, $CDCl_3$) δ 7.85-7.78 (m, 2H), 7.55-7.47 (m, 1H), 7.42-7.35 (m, 2H), 7.28-7.22 (m, 1H), 7.11-7.05 (m, 1H), 6.95 (td, J = 7.5, 1.0 Hz, 1H), 6.87 (d, J = 7.8 Hz, 1H), 3.84 (d, J = 17.7 Hz, 1H), 3.64 (d, J = 17.7 Hz, 1H), 3.28 (s, 3H), 2.32-2.11 (m, 1H), 1.03 (d, J = 6.9 Hz, 3H), 0.74 (d, J = 6.7 Hz, 3H).

^{13}C NMR (101 MHz, $CDCl_3$) δ 196.4, 180.1, 145.1, 136.6, 133.0, 130.5, 128.4, 127.9, 127.8, 122.8, 121.7, 107.7, 52.5, 44.1, 35.8, 26.2, 17.3, 16.8.

HRMS: (ESI) calcd for $C_{20}H_{21}NO_2H^+[M+H]^+$ 308.1645; found 308.1646.

1,3-dimethyl-3-(2-oxo-2-phenylethyl)-1,3-dihydro-2H-pyrrolo[2,3-b]pyridin-2-one (3o)

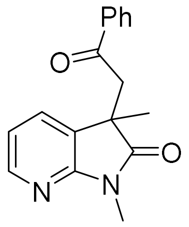

Chemical Formula: $C_{17}H_{16}N_2O_2$
Exact Mass: 280.12

3o was prepared according to general procedure **2.1** using *N*-(3-bromopyridin-2-yl)-*N*-methylmethacrylamide (0.1 mmol, 25.5 mg) and benzoic anhydride (0.2 mmol, 45.2 mg) and

was purified by silica gel column chromatography (petroleum ether/ethyl acetate =10:1~5/1) to obtain **3o** as yellow oil (17.1 mg, 61% yield). TLC (petroleum ether: ethyl acetate, 5:1 v/v): Rf = 0.3. The 1H NMR data matched those reported in the literature.[5]

^{1}H NMR (400 MHz, $CDCl_3$) δ 8.17 (dd, *J* = 5.2, 1.5 Hz, 1H), 7.93-7.78 (m, 2H), 7.59-7.49 (m, 1H), 7.47-7.34 (m, 3H), 6.87 (dd, *J* = 7.2, 5.3 Hz, 1H), 3.69 (dd, *J* = 21.3, 18.2 Hz, 2H), 3.39 (s, 3H), 1.47 (s, 3H).

^{13}C NMR (101 MHz, $CDCl_3$) δ 195.8, 180.2, 157.3, 146.6, 136.1, 133.4, 129.3, 128.6, 128.2, 128.0, 117.8, 45.6, 45.0, 25.6, 24.1.

1,3-dimethyl-3-(2-oxo-2-phenylethyl)-6-phenylindolin-2-one (3p)

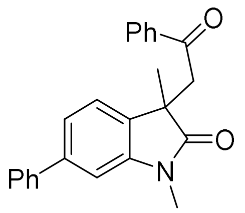

Chemical Formula: $C_{24}H_{21}NO_2$
Exact Mass: 355.1572

3p was prepared according to general procedure **2.1** using 3-(*N*-methylmethacrylamido)-[1,1'-biphenyl]-4-yl trifluoromethanesulfonate (0.1 mmol, 39.9 mg) and benzoic anhydride (0.2 mmol, 45.2 mg) and was purified by silica gel column chromatography (petroleum ether/ethyl acetate =10:1~5/1) to obtain **3p** as yellow oil (18.1 mg, 51% yield). TLC (petroleum ether: ethyl acetate, 5:1 v/v): Rf = 0.3.

^{1}H NMR (400 MHz, $CDCl_3$) δ 7.88-7.83 (m, 2H), 7.61-7.56 (m, 2H), 7.56-7.49 (m, 1H), 7.47-7.38 (m, 4H), 7.38-7.32 (m, 1H), 7.21-7.18 (m, 2H), 7.11-7.07 (m, 1H), 3.76 (d, *J* = 18.0 Hz, 1H), 3.68 (d, *J* = 18.0 Hz, 1H), 3.37 (s, 3H), 1.48 (s, 3H).

^{13}C NMR (101 MHz, $CDCl_3$) δ 196.2, 180.8, 144.4, 141.4, 141.3, 136.3, 133.2, 132.8, 128.7, 128.5, 128.0, 127.4, 127.2, 122.0, 121.2, 107.2, 46.0, 45.2, 26.5, 25.0.

HRMS: (ESI) calcd for $C_{24}H_{21}NO_2H^+$ $[M+H]^+$ 356.1645; found 356.1645.

1,3-dimethyl-3-(2-oxo-2-phenylethyl)-1,3-dihydro-2H-benzo[f]indol-2-one (3q)

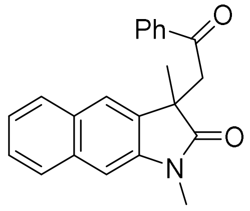

Chemical Formula: $C_{22}H_{19}NO_2$
Exact Mass: 329.1416

3q was prepared according to general procedure **2.1** using 3-(*N*-methylmethacrylamido)naphthalen-2-yl trifluoromethanesulfonate (0.1 mmol, 37.3 mg) and benzoic anhydride (0.2 mmol, 45.2 mg) and was purified by silica gel column chromatography (petroleum ether/ethyl acetate = 5/1) to obtain **3q** as white solid (19.7 mg, 60% yield). TLC (petroleum ether: ethyl acetate, 5:1 v/v): Rf = 0.3. m.p. 147.2-149.0 °C.

^{1}H NMR (400 MHz, $CDCl_3$) δ 7.86-7.82 (m, 2H), 7.78 (d, *J* = 7.7 Hz, 1H), 7.69 (d, *J* = 8.1 Hz, 1H), 7.54-7.48 (m, 2H), 7.45-7.36 (m, 3H), 7.34-7.28 (m, 1H), 7.18 (s, 1H), 3.84 (d, *J* = 18.1 Hz, 1H), 3.78 (d, *J* = 18.1 Hz, 1H), 3.42 (s, 3H), 1.52 (s, 3H).

^{13}C NMR (101 MHz, $CDCl_3$) δ 196.0, 180.2, 142.4, 136.2, 135.0, 133.8, 133.2, 130.2, 128.5, 128.0, 127.8, 127.1, 126.2, 123.9, 120.7, 103.8, 46.6, 44.7, 26.7, 25.5.

HRMS: (ESI) calcd for $C_{22}H_{19}NO_2H^+$ $[M+H]^+$ 330.1489; found 330.1488.

1,3-dimethyl-3-(2-oxo-2-(p-tolyl)ethyl)indolin-2-one (3r)

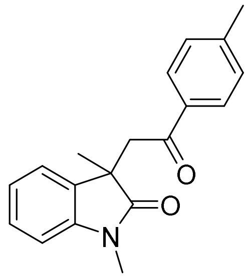

Chemical Formula: $C_{19}H_{19}NO_2$
Exact Mass: 293.1416

3r was prepared according to general procedure **2.1** using *N*-(2-bromophenyl)-*N*-methylmethacrylamide (0.1 mmol, 25.4 mg) and 4-methylbenzoic anhydride (0.2 mmol, 50.8 mg) and was purified by silica gel column chromatography (petroleum ether/ethyl acetate =10:1~5/1) to obtain **3r** as colorless oil (26.7 mg, 91% yield). TLC (petroleum ether: ethyl acetate, 5:1 v/v): Rf = 0.4. The ^{1}H NMR data matched those reported in the literature.[4]

1H NMR (400 MHz, $CDCl_3$) δ 7.77-7.70 (m, 2H), 7.25 (td, *J* = 7.7, 1.3 Hz, 1H), 7.21-7.16 (m, 2H), 7.15-7.11 (m, 1H), 6.97 (td, *J* = 7.6, 1.0 Hz, 1H), 6.89 (d, *J* = 8.1 Hz, 1H), 3.70 (d, *J* = 17.2 Hz, 1H), 3.61 (d, *J* = 17.6 Hz, 1H), 3.31 (s, 3H), 2.37 (s, 3H), 1.43 (s, 3H).

^{13}C NMR (101 MHz, $CDCl_3$) δ 195.7, 180.7, 144.0, 143.8, 133.9, 133.8, 129.1, 128.1, 127.8, 122.1, 121.7, 108.1, 45.9, 45.3, 26.4, 24.9, 21.6.

3-(2-(3,5-dimethylphenyl)-2-oxoethyl)-1,3-dimethylindolin-2-one (3s)

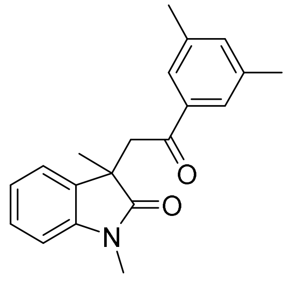

Chemical Formula: $C_{20}H_{21}NO_2$
Exact Mass: 307.1572

3s was prepared according to general procedure **2.1** using *N*-(2-bromophenyl)-*N*-methylmethacrylamide (0.1 mmol, 25.4 mg) and 3,5-dimethylbenzoic anhydride (0.2 mmol, 56.4 mg) at 80 °C and was purified by silica gel column chromatography (petroleum ether/ethyl acetate =10:1~5/1) to obtain **3s** as white solid (23.8 mg, 62% yield). TLC (petroleum ether: ethyl acetate, 5:1 v/v): Rf = 0.4. m.p. 105.2-106.8 °C. The ^{1}H NMR data matched those reported in the literature.[3]

^{1}H NMR (400 MHz, $CDCl_3$) δ 7.44 (s, 2H), 7.27-7.22 (m, 1H), 7.17-7.10 (m, 2H), 6.96 (td, *J* = 7.5, 1.0 Hz, 1H), 6.89 (d, *J* = 7.7 Hz, 1H), 3.70 (d, *J* = 17.8 Hz, 1H), 3.61 (d, *J* = 17.6 Hz, 1H), 3.31 (s, 3H), 2.31 (s, 6H), 1.44 (s, 3H).

^{13}C NMR (101 MHz, $CDCl_3$) δ 196.5, 180.7, 143.9, 138.1, 136.4, 134.8, 133.8, 127.8, 125.8, 122.1, 121.7, 108.1, 46.1, 45.3, 26.4, 24.8, 21.1.

3-(2-(4-fluorophenyl)-2-oxoethyl)-1,3-dimethylindolin-2-one (3t)

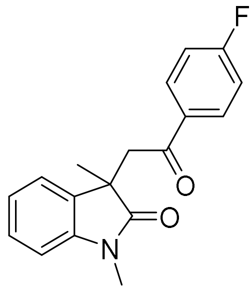

Chemical Formula: $C_{18}H_{16}FNO_2$
Exact Mass: 297.1165

3t was prepared according to general procedure **2.1** using *N*-(2-bromophenyl)-*N*-methylmethacrylamide (0.1 mmol, 25.4 mg) and 4-fluorobenzoic anhydride (0.2 mmol, 52.4 mg) and was purified by silica gel column chromatography (petroleum ether/ethyl acetate =10:1~5/1) to obtain **3t** as white solid (23.8 mg, 80% yield). TLC (petroleum ether: ethyl acetate, 5:1 v/v): Rf = 0.4. m.p. 107.8-109.5 °C. The ^{1}H NMR data matched those reported in the literature.[4]

^{1}H NMR (400 MHz, $CDCl_3$) δ 7.92-7.80 (m, 2H), 7.29-7.23 (m, 1H), 7.16-7.11 (m, 1H), 7.11-7.02 (m, 2H), 6.98 (td, *J* = 7.5, 1.0 Hz, 1H), 6.90 (d, *J* = 7.8 Hz, 1H), 3.65 (d, *J* = 2.3 Hz, 2H), 3.31 (s, 3H), 1.44 (s, 3H).

^{13}C NMR (101 MHz, $CDCl_3$) δ 194.5, 180.5, 165.7 (d, *J* = 254.8 Hz), 143.8, 133.6, 132.7 (m), 130.60 (d, *J* = 9.5 Hz), 127.9, 122.2, 121.7, 115.6 (d, *J* = 22.0 Hz), 108.2, 45.9, 45.3, 26.4, 24.9.

^{19}F NMR (377 MHz, $CDCl_3$) δ -104.8(s).

1,3-dimethyl-3-(2-oxo-3-phenylpropyl)indolin-2-one (3u)

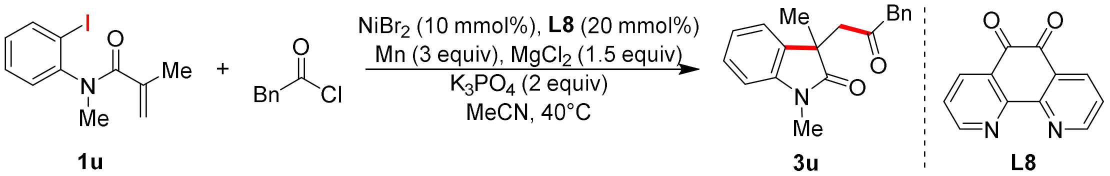

An oven-dried sealed tube equipped with a PTFE-coated stir bar was charged with $NiBr_2$ (10 mol%), **L8** (20 mol%), *N*-(2-iodophenyl)-*N*-methylmethacrylamide (0.1 mmol, 1.0 equiv), manganese powder (3.0 equiv), $MgCl_2$ (1.5 equiv) and K_3PO_4 (2.0 equiv). The sealed tube was evacuated and backfilled with argon (this process was repeated for three times) and then MeCN (2 mL) was added. This reaction mixture was stirred at room temperature for 15 minutes and then 2-phenylacetyl chloride (3.0 equiv) was added. Then, the reaction heated at 40 °C for 36

hours. The resulting mixture was purified by chromatography on silica gel, eluting with ethyl acetate/petroleum ether 1:20~1:5 (v/v) to afford the corresponding product **3u** as yellow oil (17.9 mg, 61% yield). TLC (petroleum ether: ethyl acetate, 5:1 v/v): Rf = 0.5. The ^{1}H NMR data matched those reported in the literature.[6]

^{1}H NMR (400 MHz, $CDCl_3$) δ 7.3-7.2 (m, 4H), 7.1-7.0 (m, 2H), 7.0-6.9 (m, 2H), 6.9-6.8 (m, 1H), 3.5 (d, J = 1.2 Hz, 2H), 3.2 (s, 3H), 3.1 (d, *J* = 3.4 Hz, 2H), 1.3 (s, 3H).

^{13}C NMR (101 MHz, $CDCl_3$) δ 204.3, 180.4, 143.8, 133.7, 133.4, 129.5, 128.8, 128.0, 127.2, 122.3, 121.9, 108.3, 50.2, 48.8, 45.3, 26.5, 24.5.

Phenyl 2-(1,3-dimethyl-2-oxoindolin-3-yl)acetate (6a)

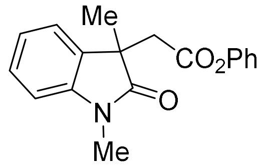

Chemical Formula: $C_{18}H_{17}NO_3$
Exact Mass: 295.1208

6a was prepared according to general procedure **2.2** using *N*-(2-bromophenyl)-*N*-methylmethacrylamide (0.1 mmol, 25.4 mg) and phenyl carbonochloridate (0.4 mmol, 62.6 mg) and was purified by silica gel column chromatography (PE/EtOAc = 15/1~5/1) to obtain **6a** as colorless oil (22.1 mg, 75% yield). TLC (petroleum ether: ethyl acetate, 5:1 v/v): Rf = 0.5. The ^{1}H NMR data matched those reported in the literature.[7]

^{1}H NMR (400 MHz, $CDCl_3$): δ 7.29 (m, 2H), 7.25-7.18 (m, 2H), 7.14-7.04 (m, 2H), 6.90-6.82 (m, 1H), 6.70-6.63 (m, 2H), 3.29 (d, *J* = 16.0 Hz, 1H), 3.20 (s, 3H), 3.06 (d, *J* = 16.0 Hz, 1H), 1.45 (s, 3H).

^{13}C NMR (101 MHz, $CDCl_3$) δ 179.6, 168.5, 150.2, 143.7, 132.5, 129.4, 128.5, 125.9, 122.6, 122.6, 121.4, 108.4, 45.8, 41.9, 26.5, 24.4.

Phenyl 2-(5-methoxy-1,3-dimethyl-2-oxoindolin-3-yl)acetate (6b)

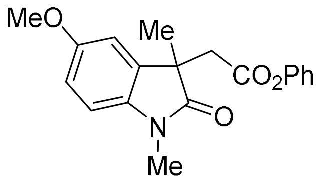

Chemical Formula: $C_{19}H_{19}NO_4$
Exact Mass: 325.1314

6b was prepared according to general procedure **2.2** using *N*-(2-bromo-4-methoxyphenyl)-*N*-methylmethacrylamide (0.1 mmol, 28.4 mg) and phenyl carbonochloridate (0.4 mmol, 62.6 mg) and was purified by silica gel column chromatography (PE/EtOAc = 5/1) to obtain **6b** as brown solid (21.1 mg, 65% yield). TLC (petroleum ether: ethyl acetate, 5:1 v/v): Rf = 0.4. m.p. 86.0-88.4 °C.

^{1}H NMR (400 MHz, $CDCl_3$): δ 7.28-7.22 (m, 2H), 7.16-7.11 (m, 1H), 6.93 (d, *J* = 2.5 Hz, 1H), 6.84-6.80 (m, 1H), 6.78-6.70 (m, 3H), 3.80 (s, 3H), 3.29 (d, *J* = 16.2 Hz, 1H), 3.19 (s, 3H), 3.04 (d, *J* = 16.2 Hz, 1H), 1.45 (s, 3H);

^{13}C NMR (400 MHz, $CDCl_3$): δ 179.3, 168.4, 156.0, 150.2, 137.2, 133.9, 129.3, 125.9, 121.4, 112.3, 110.3, 108.6, 55.9, 46.1, 41.8, 26.5, 24.4;

HRMS: (ESI) calcd for $C_{19}H_{19}NNaO_4^+$[M+Na]$^+$ 348.1212; found 348.1194.

Phenyl 2-(1,3,5-trimethyl-2-oxoindolin-3-yl)acetate (6c)

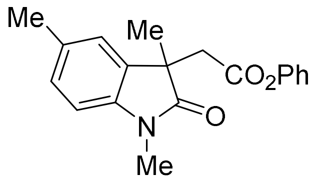

Chemical Formula: $C_{19}H_{19}NO_3$
Exact Mass: 309.1365

6c was prepared according to general procedure **2.2** using *N*-(2-bromo-4-methylphenyl)-*N*-methylmethacrylamide (0.1 mmol, 26.8 mg) and phenyl carbonochloridate (0.4 mmol, 62.6 mg) and was purified by silica gel column chromatography (PE/EtOAc = 15/1~5/1) to obtain **6c** as yellow solid (19.1 mg, 62% yield). TLC (petroleum ether: ethyl acetate, 5:1 v/v): Rf = 0.5. m.p. 100.1-103.0 °C.

^{1}H NMR (400 MHz, $CDCl_3$): δ 7.28-7.21 (m, 2H), 7.16-7.07 (m, 3H), 6.75 (d, *J* = 7.8 Hz, 1H), 6.71-6.65 (m, 2H), 3.28 (d, *J* = 16.0 Hz, 1H), 3.19 (s, 3H), 3.04 (d, *J* = 16.0 Hz, 1H), 2.35 (s,

3H), 1.45 (s, 3H);

^{13}C NMR (101 MHz, $CDCl_3$): δ 179.6, 168.5, 150.2, 141.3, 132.6, 132.1, 129.4, 128.6, 125.9, 123.5, 121.4, 108.1, 45.8, 41.9, 26.5, 24.4, 21.2;

HRMS: (ESI) calcd for $C_{19}H_{19}NNaO_3^+$[M+Na]$^+$ 332.1263; found 332.1261.

Phenyl 2-(5-fluoro-1,3-dimethyl-2-oxoindolin-3-yl)acetate (6d)

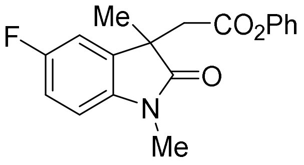

Chemical Formula: $C_{18}H_{16}FNO_3$
Exact Mass: 313.1114

6d was prepared according to general procedure **2.2** using *N*-(2-bromo-4-fluorophenyl)-*N*-methylmethacrylamide (0.1 mmol, 27.2 mg) and phenyl carbonochloridate (0.4 mmol, 62.6 mg) and was purified by silica gel column chromatography (PE/EtOAc = 15/1~5/1) to obtain **6d** as yellow solid (20.0 mg, 64% yield). TLC (petroleum ether: ethyl acetate, 5:1 v/v): Rf = 0.5. m.p. 81.4-84.3 °C.

^1H NMR (400 MHz, $CDCl_3$): δ 7.31-7.23 (m, 2H), 7.18-7.12 (m, 1H), 7.09–7.05 (m, 1H), 7.04–6.97 (m, 1H), 6.80-6.74 (m, 3H), 3.30 (d, J = 16.5 Hz, 1H), 3.21 (s, 3H), 3.06 (d, J = 16.5 Hz, 1H), 1.45 (s, 3H);

^{13}C NMR (101 MHz, $CDCl_3$): δ 179.3, 168.3, 159.3 (d, J = 240.8 Hz), 150.1, 139.7, 134.2, 129.4, 126.0, 121.3, 114.5 (d, J = 23.4 Hz), 110.8 (d, J = 24.9 Hz), 108.8 (d, J = 8.1 Hz), 46.1, 41.6, 26.6, 24.3;

^{19}F NMR (377 MHz, $CDCl_3$) δ -120.5(s).

HRMS: (ESI) calcd for $C_{18}H_{16}FNNaO_3^+$[M+Na]$^+$ 336.1012; found 336.1007.

Phenyl 2-(1,3-dimethyl-2-oxo-5-(trifluoromethyl)indolin-3-yl)acetate (6e)

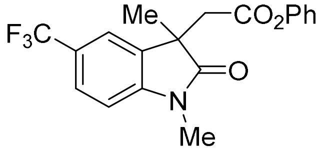

Chemical Formula: $C_{19}H_{16}F_3NO_3$
Exact Mass: 363.1082

6e was prepared according to general procedure **2.2** using *N*-(2-bromo-4-(trifluoromethyl)phenyl)-*N*-methylmethacrylamide (0.1 mmol, 32.2 mg) and phenyl carbonochloridate (0.4 mmol, 62.6 mg) and was purified by silica gel column chromatography (PE/EtOAc = 15/1~5/1) to obtain **6e** as brown solid (23.9 mg, 66% yield). TLC (petroleum ether: ethyl acetate, 5:1 v/v): Rf = 0.5. m.p. 86.8-88.6 °C.

^{1}H NMR (400 MHz, $CDCl_3$): δ 7.62-7.56 (m, 1H), 7.53 (d, *J* = 1.8 Hz, 1H), 7.31-7.23 (m, 2H), 7.18-7.12 (m, 1H), 6.92 (d, *J* = 8.2 Hz, 1H), 6.79-6.77 (m, 1H), 6.76-6.74 (m, 1H), 3.34 (d, *J* = 16.7 Hz, 1H), 3.25 (s, 3H), 3.14 (d, *J* = 16.7 Hz, 1H), 1.47 (s, 3H);

^{13}C NMR (101 MHz, $CDCl_3$): δ 179.6, 168.3, 150.0, 146.8, 133.3, 129.5, 126.2 (q, *J* = 4.0 Hz), 126.1, 125.3 (d, *J* = 91.3 Hz), 123.8 (d, *J* = 147.5 Hz), 121.2, 119.4 (q, *J* = 3.6 Hz), 108.1, 45.6, 41.6, 26.7, 24.3;

^{19}F NMR (377 MHz, $CDCl_3$) δ -61.2(s).

HRMS: (ESI) calcd for $C_{19}H_{16}F_3NNaO_3^+$[M+Na]$^+$ 386.0980; found 386.0976.

Phenyl 2-(6-methoxy-1,3-dimethyl-2-oxoindolin-3-yl)acetate (6f)

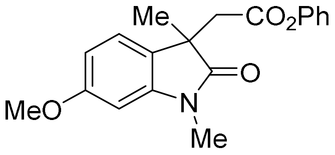

Chemical Formula: $C_{19}H_{19}NO_4$
Exact Mass: 325.1314

6f was prepared according to general procedure **2.2** using *N*-(2-bromo-5-methoxyphenyl)-*N*-methylmethacrylamide (0.1 mmol, 28.4 mg) and phenyl carbonochloridate (0.4 mmol, 62.6 mg) and was purified by silica gel column chromatography (PE/EtOAc =15/1~5/1) to obtain **6f** as colorless oil (19.5 mg, 60% yield). TLC (petroleum ether: ethyl acetate, 5:1 v/v): Rf = 0.4.

^{1}H NMR (400 MHz, $CDCl_3$): δ 7.31-7.22 (m, 2H), 7.20 (d, *J* = 8.1 Hz, 1H), 7.17-7.11 (m, 1H), 6.76-6.68 (m, 2H), 6.62–6.55 (m, 1H), 6.45 (d, *J* = 2.3 Hz, 1H), 3.83 (s, 3H), 3.25 (d, *J* = 15.9 Hz, 1H), 3.20 (s, 3H), 3.03 (d, *J* = 15.9 Hz, 1H), 1.44 (s, 3H);

^{13}C NMR (101 MHz, $CDCl_3$); δ 180.2, 168.6, 160.4, 150.2, 145.0, 129.4, 125.9, 124.4, 123.2, 121.4, 106.2, 96.4, 55.6, 45.3, 42.1, 26.5, 24.5;

HRMS: (ESI) calcd for $C_{19}H_{19}NNaO_4^+$[M+Na]$^+$ 348.1212; found 348.1208.

Phenyl 2-(6-chloro-1,3-dimethyl-2-oxoindolin-3-yl)acetate (6g)

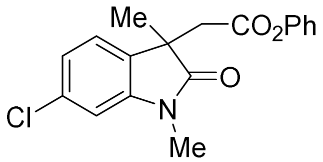

Chemical Formula: $C_{18}H_{16}ClNO_3$
Exact Mass: 329.0819

6g was prepared according to general procedure **2.2** using *N*-(2-bromo-5-chlorophenyl)-*N*-methylmethacrylamide (0.1 mmol, 28.8 mg) and phenyl carbonochloridate (0.4 mmol, 62.6 mg) and was purified by silica gel column chromatography (PE/EtOAc = 15/1~5/1) to obtain **6g** as white solid (18.7 mg, 57% yield). TLC (petroleum ether: ethyl acetate, 5:1 v/v): Rf = 0.5. m.p. 113.0-114.6 °C.

^{1}H NMR (400 MHz, $CDCl_3$): δ 7.31-7.24 (m, 2H), 7.21 (d, *J* = 7.9 Hz, 1H), 7.18-7.12 (m, 1H), 7.09-7.03 (m, 1H), 6.86 (d, *J* = 1.8 Hz, 1H), 6.77-6.75 (m, 1H), 6.75-6.73 (m, 1H), 3.29 (d, *J* = 16.4 Hz, 1H), 3.20 (s, 3H), 3.06 (d, *J* = 16.4 Hz, 1H), 1.44 (s, 3H);

^{13}C NMR (101 MHz, $CDCl_3$): δ 179.6, 168.3, 150.1, 145.0, 134.2, 131.0, 129.5, 126.0, 123.4, 122.3, 121.3, 109.1, 45.4, 41.7, 26.6, 24.3;

HRMS: (ESI) calcd for $C_{18}H_{16}ClNNaO_3^+$[M+Na]$^+$ 352.0717; found 352.0709.

Phenyl 2-(1,3,6-trimethyl-2-oxoindolin-3-yl)acetate (6h)

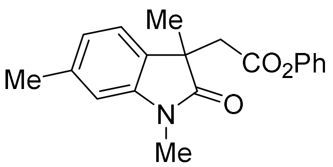

Chemical Formula: $C_{19}H_{19}NO_3$
Exact Mass: 309.1365

6h was prepared according to general procedure **2.2** using *N*-(2-bromo-5-methylphenyl)-*N*-methylmethacrylamide (0.1 mmol, 26.8 mg) and phenyl carbonochloridate (0.4 mmol, 62.6 mg) and was purified by silica gel column chromatography (PE/EtOAc = 15/1~5/1) to obtain **6h** as white solid (18.8 mg, 61% yield). TLC (petroleum ether: ethyl acetate, 5:1 v/v): Rf = 0.5. m.p. 77.9-79.3 °C.

^{1}H NMR (400 MHz, $CDCl_3$): δ 7.28-7.21 (m, 2H), 7.18 (m, 1H), 7.16-7.10 (m, 1H), 6.93-6.86

(m, 1H), 6.74-6.66 (m, 3H), 3.26 (d, J = 16.1 Hz, 1H), 3.20 (s, 3H), 3.04 (d, J = 16.0 Hz, 1H), 2.39 (s, 3H), 1.44 (s, 3H);

^{13}C NMR (101 MHz, $CDCl_3$): δ 180.0, 168.5, 150.2, 143.8, 138.5, 129.6, 129.3, 125.9, 123.0, 122.3, 121.4, 109.3, 45.6, 41.9, 26.4, 24.4, 21.9;

HRMS: (ESI) calcd for $C_{19}H_{19}NNaO_3^+$[M+Na]$^+$ 332.1263; found 332.1258.

Phenyl 2-(1,3,7-trimethyl-2-oxoindolin-3-yl)acetate (6i)

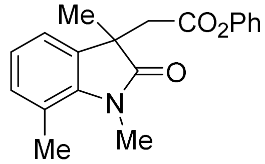

Chemical Formula: $C_{19}H_{19}NO_3$
Exact Mass: 309.1365

6i was prepared according to general procedure **2.2** using *N*-(2-bromo-6-methylphenyl)-*N*-methylmethacrylamide (0.1 mmol, 26.8 mg) and phenyl carbonochloridate (0.4 mmol, 62.6 mg) and was purified by silica gel column chromatography (PE/EtOAc = 15/1~5/1) to obtain **6i** as yellow solid (13.3 mg, 43% yield). TLC (petroleum ether: ethyl acetate, 5:1 v/v): Rf = 0.5. m.p. 103.4-104.8 °C.

^{1}H NMR (400 MHz, $CDCl_3$): δ 7.29–7.22 (m, 2H), 7.17-7.09 (m, 2H), 7.04-7.00 (m, 1H), 7.00–6.94 (m, 1H), 6.71–6.69 (m, 1H), 6.69-6.66 (m, 1H), 3.49 (s, 3H), 3.30 (d, J = 16.0 Hz, 1H), 3.03 (d, J = 16.0 Hz, 1H), 2.56 (s, 3H), 1.43 (s, 3H);

^{13}C NMR (101 MHz, $CDCl_3$): δ 180.4, 168.5, 150.2, 141.5, 133.2, 132.1, 129.4, 125.9, 122.5, 121.4, 120.3, 112.0, 45.1, 42.2, 29.8, 24.8, 19.1;

HRMS: (ESI) calcd for $C_{19}H_{19}NNaO_3^+$[M+Na]$^+$ 332.1263; found 332.1260.

phenyl 2-(1,3,4-trimethyl-2-oxoindolin-3-yl)acetate (6j)

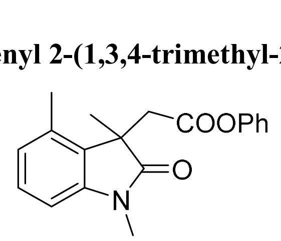

Chemical Formula: $C_{19}H_{19}NO_3$
Exact Mass: 309.1365

6j was prepared according to general procedure **2.2** using *N*-(2-bromo-3-methylphenyl)-*N*-

methylmethacrylamide (0.1 mmol, 26.8 mg) and phenyl carbonochloridate (0.4 mmol, 62.6 mg) and was purified by silica gel column chromatography (PE/EtOAc = 15/1~5/1) to obtain **6j** as white solid (13.6 mg, 44% yield). TLC (petroleum ether: ethyl acetate, 5:1 v/v): Rf = 0.4. m.p. 82.5-84.7 °C.

^{1}H NMR (400 MHz, $CDCl_3$) δ 7.24-7.16 (m, 3H), 7.13-7.07 (m, 1H), 6.86 (d, *J* = 7.8 Hz, 1H), 6.71 (d, *J* = 7.7 Hz, 1H), 6.53-6.46 (m, 2H), 3.41 (d, *J* = 15.3 Hz, 1H), 3.24-3.18 (m, 4H), 2.46 (s, 3H), 1.52 (s, 3H).

^{13}C NMR (101 MHz, $CDCl_3$) δ 179.6, 168.4, 150.1, 144.1, 134.2, 129.2, 128.3, 125.8, 125.1, 121.2, 106.1, 46.8, 41.3, 26.5, 22.4, 18.2.

HRMS: (ESI) calcd for $C_{19}H_{19}NO_3H^+[M+H]^+$ 310.1438; found 310.1440.

phenyl 2-(1,3-dimethyl-2-oxo-2,3-dihydro-1H-pyrrolo[2,3-b]pyridin-3-yl)acetate (**6k**)

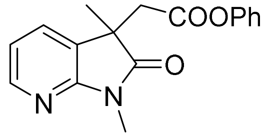

Chemical Formula: $C_{17}H_{16}N_2O_3$
Exact Mass: 296.1161

6k was prepared according to general procedure **2.2** using *N*-(3-bromopyridin-2-yl)-*N*-methylmethacrylamide (0.1 mmol, 25.5 mg) and phenyl carbonochloridate (0.4 mmol, 62.6 mg) and was purified by silica gel column chromatography (PE/EtOAc = 15/1~5/1) to obtain **6k** as colorless oil (18.0 mg, 61% yield). TLC (petroleum ether: ethyl acetate, 5:1 v/v): Rf = 0.4.

^{1}H NMR (600 MHz, $CDCl_3$) δ 8.21 (dd, *J* = 5.3, 1.6 Hz, 1H), 7.56 (dd, *J* = 7.2, 1.6 Hz, 1H), 7.30-7.26 (m, 2H), 7.19-7.14 (m, 1H), 6.97 (dd, *J* = 7.3, 5.3 Hz, 1H), 6.81-6.73 (m, 2H), 3.33-3.26 (m, 4H), 3.09 (d, *J* = 16.5 Hz, 1H), 1.49 (s, 3H).

^{13}C NMR (151 MHz, $CDCl_3$) δ 179.2, 168.2, 157.1, 150.0, 147.1, 130.1, 129.4, 127.1, 126.0, 121.2, 118.1, 45.3, 41.3, 25.6, 23.6.

HRMS: (ESI) calcd for $C_{17}H_{16}N_2O_3H^+[M+H]^+$ 297.1234; found 297.1236.

Phenyl 2-(1-benzyl-3-methyl-2-oxoindolin-3-yl)acetate (**6l**)

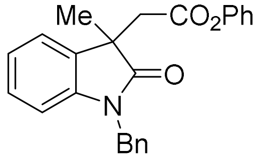

Chemical Formula: $C_{24}H_{21}NO_3$
Exact Mass: 371.1521

6l was prepared according to general procedure **2.2** using *N*-benzyl-*N*-(2-bromophenyl)methacrylamide (0.1 mmol, 33.0 mg) and phenyl carbonochloridate (0.4 mmol, 62.6 mg) and was purified by silica gel column chromatography (PE/EtOAc = 10/1~5/1) to obtain **6l** as yellow oil (16.0 mg, 43% yield). TLC (petroleum ether: ethyl acetate, 5:1 v/v): Rf = 0.5.

^{1}H NMR (400 MHz, $CDCl_3$): δ 7.34-7.29 (m, 1H), 7.28-7.24 (m, 2H), 7.24-7.09 (m, 7H), 7.08-7.02 (m, 1H), 6.75-6.70 (m, 1H), 6..61-6.58 (m, 1H), 6.57-6.55 (m, 1H), 4.92 (q, *J* = 15.7 Hz, 2H), 3.39 (d, *J* = 15.9 Hz, 1H), 3.10 (d, *J* = 15.9 Hz, 1H), 1.52 (s, 3H);

^{13}C NMR (101 MHz, $CDCl_3$): δ 179.7, 168.5, 150.2, 142.8, 135.8, 132.5, 129.3, 128.7, 128.3, 127.5, 127.3, 125.9, 122.7, 122.6, 121.4, 109.5, 45.9, 44.0, 41.8, 25.2;

HRMS: (ESI) calcd for $C_{24}H_{21}NNaO_3^+$[M+Na]$^+$ 394.1419; found 394.1417.

Phenyl 2-(3-(methoxymethyl)-1-methyl-2-oxoindolin-3-yl)acetate (**6m**)

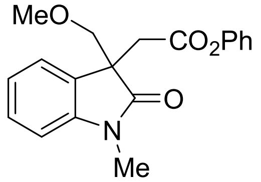

Chemical Formula: $C_{19}H_{19}NO_4$
Exact Mass: 325.1314

6m was prepared according to general procedure **2.2** using *N*-(2-bromophenyl)-2-(methoxymethyl)-*N*-methylacrylamide (0.1 mmol, 28.4 mg) and phenyl carbonochloridate (0.4 mmol, 62.6 mg) and was purified by silica gel column chromatography (PE/EtOAc = 5/1) to obtain **6m** as yellow oil (16.5 mg, 51% yield). TLC (petroleum ether: ethyl acetate, 5:1 v/v): Rf = 0.4.

^{1}H NMR (400 MHz, $CDCl_3$) δ 7.43-7.38 (m, 1H), 7.36-7.30 (m, 1H), 7.27-7.20 (m, 2H), 7.15-7.05 (m, 2H), 6.86 (d, *J* = 7.8 Hz, 1H), 6.68-6.65 (m, 1H), 6.65-6.62 (m, 1H), 3.71 (d, *J* = 8.8

Hz, 1H), 3.52 (d, J = 8.8 Hz, 1H), 3.30 (s, 3H), 3.26 (s, 2H), 3.21 (s, 3H);

^{13}C NMR (101 MHz, $CDCl_3$): δ 177.1, 168.4, 150.2, 144.4, 129.7, 129.3, 128.7, 125.9, 123.8, 122.5, 121.3, 108.2, 76.2, 59.7, 50.9, 37.9, 26.5.

HRMS: (ESI) calcd for $C_{19}H_{19}NNaO_4^+$[M+Na]$^+$ 348.1212; found 348.1212.

Phenyl 2-(3-benzyl-1-methyl-2-oxoindolin-3-yl)acetate (6n)

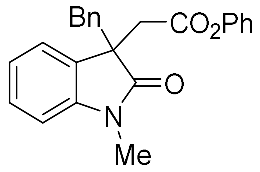

Chemical Formula: $C_{24}H_{21}NO_3$
Exact Mass: 371.1521

6n was prepared according to general procedure **2.2** using 2-benzyl-N-(2-bromophenyl)-N-methylacrylamide (0.1 mmol, 33.0 mg) and phenyl carbonochloridate (0.4 mmol, 62.6 mg) and was purified by silica gel column chromatography (PE/EtOAc = 15/1~5/1) to obtain **6n** as yellow oil (26.1 mg, 70% yield). TLC (petroleum ether: ethyl acetate, 5:1 v/v): Rf = 0.5.

^{1}H NMR (400 MHz, $CDCl_3$): δ 7.27-7.18 (m, 4H), 7.14-7.03 (m, 5H), 6.89-6.84 (m, 2H), 6.66-6.63 (m, 1H), 6.63-6.59 (m, 2H), 3.44 (d, J = 15.9 Hz, 1H), 3.17 (d, J = 16.0 Hz, 1H), 3.12 (s, 2H), 2.97 (s, 3H);

^{13}C NMR (101 MHz, $CDCl_3$): δ 178.2, 168.4, 150.2, 144.26, 134.7, 130.1, 129.8, 129.3, 128.5, 127.6, 126.9, 125.9, 123.5, 122.1, 121.3, 108.0, 51.4, 44.0, 40.6, 26.1.

HRMS: (ESI) calcd for $C_{24}H_{21}NNaO_3^+$[M+Na]$^+$ 394.1419; found 394.1416.

Phenyl 2-(3-hexyl-1-methyl-2-oxoindolin-3-yl)acetate (6o)

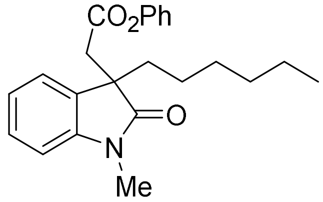

Chemical Formula: $C_{23}H_{27}NO_3$
Exact Mass: 365.1991

6o was prepared according to general procedure **2.2** using *N*-(2-bromophenyl)-*N*-methyl-2-methyleneoctanamide (0.1 mmol, 32.4 mg) and phenyl carbonochloridate (0.4 mmol, 62.6 mg)

and was purified by silica gel column chromatography (PE/EtOAc = 15/1~5/1) to obtain **6o** as brown oil (22.2 mg, 63% yield). TLC (petroleum ether: ethyl acetate, 5:1 v/v): Rf = 0.5.

^{1}H NMR (400 MHz, CDCl$_3$): δ 7.35-7.18 (m, 4H), 7.15-7.05 (m, 2H), 6.85 (d, *J* = 7.8 Hz, 1H), 6.65-6.58 (m, 2H), 3.28 (d, *J* = 15.8 Hz, 1H), 3.20 (s, 3H), 3.06 (d, *J* = 15.8 Hz, 1H), 1.86 (dtd, *J* = 45.9, 12.9, 4.4 Hz, 2H), 1.36-0.70 (m, 13H);

^{13}C NMR (101 MHz, CDCl$_3$): δ 179.1, 168.5, 150.2, 144.5, 130.9, 129.3, 128.37, 125.8, 122.8, 122.5, 121.3, 108.1, 50.0, 41.7, 38.3, 31.5, 29.3, 26.4, 23.6, 22.6, 14.0;

HRMS: (ESI) calcd for $C_{23}H_{27}NNaO_3^+$[M+Na]$^+$ 388.1889; found 388.1881.

Phenyl 2-(3-isopropyl-1-methyl-2-oxoindolin-3-yl)acetate (6p)

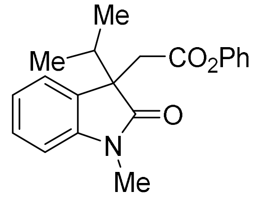

Chemical Formula: $C_{20}H_{21}NO_3$
Exact Mass: 323.1521

6p was prepared according to general procedure **2.2** using *N*-(2-bromophenyl)-*N*,3-dimethyl-2-methylenebutanamide (0.1 mmol, 29.6 mg) and phenyl carbonochloridate (0.4 mmol, 62.6 mg) and was purified by silica gel column chromatography (PE/EtOAc = 15/1~5/1) to obtain **6p** as yellow solid (21.4 mg, 66% yield). TLC (petroleum ether: ethyl acetate, 5:1 v/v): Rf = 0.5. m.p. 75.8-78.2 °C.

^{1}H NMR (400 MHz, CDCl$_3$): δ 7.35-7.29 (m, 1H), 7.28-7.25 (m, 1H), 7.24-7.18 (m, 2H), 7.14-7.04 (m, 2H), 6.87-6.81 (m, 1H), 6.61-6.58 (m, 1H), 6.58-6.55 (m, 1H), 3.32 (d, *J* = 15.8 Hz, 1H), 3.20 (d, *J* = 9.1 Hz, 4H), 2.20 (p, *J* = 6.8 Hz, 1H), 0.99 (d, *J* = 6.9 Hz, 3H), 0.81 (d, *J* = 6.7 Hz, 3H);

^{13}C NMR (101 MHz, CDCl$_3$) δ 179.1, 168.8, 150.2, 144.9, 129.7, 129.3, 128.4, 125.8, 123.5, 122.1, 121.3, 108.0, 53.2, 39.9, 35.6, 26.2, 17.3, 16.9.

HRMS: (ESI) calcd for $C_{20}H_{21}NNaO_3^+$[M+Na]$^+$ 346.1419; found 346.1410.

isobutyl 2-(1,3-dimethyl-2-oxoindolin-3-yl)acetate (6q)

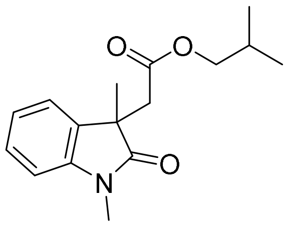

Chemical Formula: $C_{16}H_{21}NO_3$
Exact Mass: 275.1521

6q was prepared according to general procedure **2.2** using *N*-(2-bromophenyl)-*N*-methylmethacrylamide (0.1 mmol, 25.4 mg) and isobutyl carbonochloridate (0.4 mmol, 54.6 mg) and was purified by silica gel column chromatography (PE/EtOAc = 15/1~5/1) to obtain **6q** as yellow oil (16.5 mg, 60% yield). TLC (petroleum ether: ethyl acetate, 5:1 v/v): Rf = 0.5.

^{1}H NMR (400 MHz, $CDCl_3$) δ 7.30-7.23 (m, 1H), 7.20 (d, *J* = 7.0 Hz, 1H), 7.03 (t, *J* = 7.5 Hz, 1H), 6.85 (d, *J* = 7.8 Hz, 1H), 3.61 (d, *J* = 6.6 Hz, 2H), 3.25 (s, 3H), 3.04 (d, *J* = 16.2 Hz, 1H), 2.86 (d, *J* = 16.2 Hz, 1H), 1.74-1.65 (m, 1H), 1.37 (s, 3H), 0.75 (t, *J* = 7.1 Hz, 6H).

^{13}C NMR (101 MHz, $CDCl_3$) δ 179.9, 169.9, 143.5, 133.0, 128.1, 122.4, 122.2, 108.1, 70.7, 45.5, 41.6, 27.5, 26.3, 24.5, 18.9.

HRMS: (ESI) calcd for $C_{16}H_{21}NO_3H^+$ $[M+H]^+$ 276.1594; found 276.1593.

propyl 2-(1,3-dimethyl-2-oxoindolin-3-yl)acetate (6r)

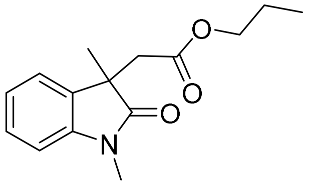

Chemical Formula: $C_{15}H_{19}NO_3$
Exact Mass: 261.1365

6r was prepared according to general procedure **2.2** using *N*-(2-bromophenyl)-*N*-methylmethacrylamide (0.1 mmol, 25.4 mg) and propyl carbonochloridate (0.4 mmol, 49.0 mg) and was purified by silica gel column chromatography (PE/EtOAc = 15/1~5/1) to obtain **6r** as yellow oil (17.7 mg, 68% yield). TLC (petroleum ether: ethyl acetate, 5:1 v/v): Rf = 0.5. The ^{1}H NMR data matched those reported in the literature.[7]

^{1}H NMR (400 MHz, $CDCl_3$) δ 7.30-7.23 (m, 1H), 7.22-7.17 (m, 1H), 7.03 (td, *J* = 7.5, 1.0 Hz, 1H), 6.85 (d, *J* = 7.8 Hz, 1H), 3.86-3.72 (m, 2H), 3.25 (s, 3H), 3.03 (d, *J* = 16.2 Hz, 1H), 2.84 (d, *J* = 16.2 Hz, 1H), 1.45-1.36 (m, 5H), 0.76 (t, *J* = 7.4 Hz, 3H).

^{13}C NMR (101 MHz, $CDCl_3$) δ 179.9, 169.9, 143.6, 133.0, 128.1, 122.4, 122.3, 108.1, 66.1, 45.5, 41.7, 26.3, 24.5, 21.7, 10.2.

butyl 2-(1,3-dimethyl-2-oxoindolin-3-yl)acetate (6s)

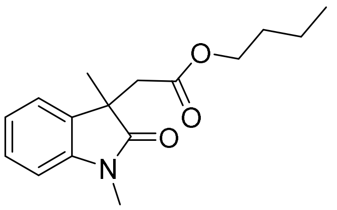

Chemical Formula: $C_{16}H_{21}NO_3$
Exact Mass: 275.1521

6s was prepared according to general procedure **2.2** using *N*-(2-bromophenyl)-*N*-methylmethacrylamide (0.1 mmol, 25.4 mg) and butyl carbonochloridate (0.4 mmol, 54.6 mg) and was purified by silica gel column chromatography (PE/EtOAc = 15/1~5/1) to obtain **6s** as yellow oil (15.7mg, 57% yield). TLC (petroleum ether: ethyl acetate, 5:1 v/v): Rf = 0.5.

^{1}H NMR (400 MHz, $CDCl_3$) δ 7.32-7.23 (m, 1H), 7.23-7.16 (m, 1H), 7.03 (td, *J* = 7.5, 1.0 Hz, 1H), 6.85 (d, *J* = 7.8 Hz, 1H), 3.91-3.75 (m, 2H), 3.25 (s, 3H), 3.03 (d, *J* = 16.2 Hz, 1H), 2.83 (d, J = 16.2 Hz, 1H), 1.40-1.30 (m, 5H), 1.22-1.11 (m, 2H), 0.82 (t, *J* = 7.3 Hz, 3H).

^{13}C NMR (101 MHz, $CDCl_3$) δ 179.9, 169.9, 143.6, 133.0, 128.1, 122.3, 122.3, 108.1, 64.4, 45.5, 41.7, 30.4, 26.3, 24.5, 18.9, 13.6.

HRMS: (ESI) calcd for $C_{16}H_{21}NO_3H^+[M+H]^+$ 276.1594; found 276.1595.

6. Copies of the ^{1}H, ^{19}F and ^{13}C NMR spectra

3a

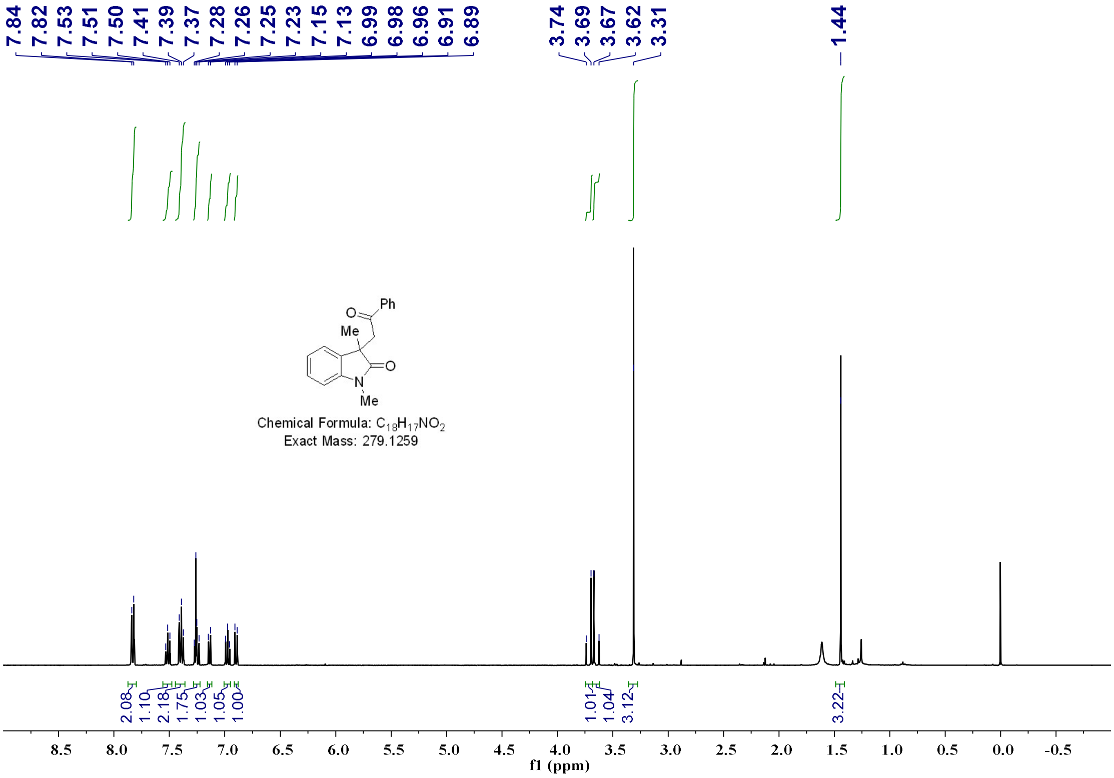

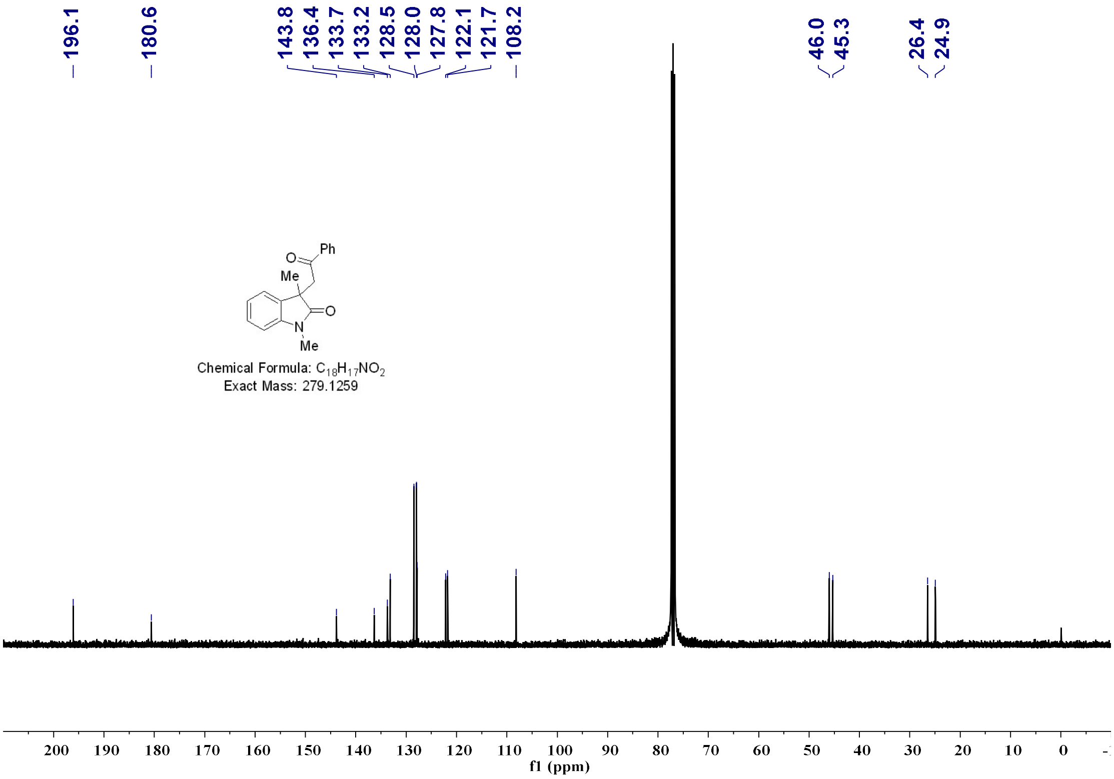

3b

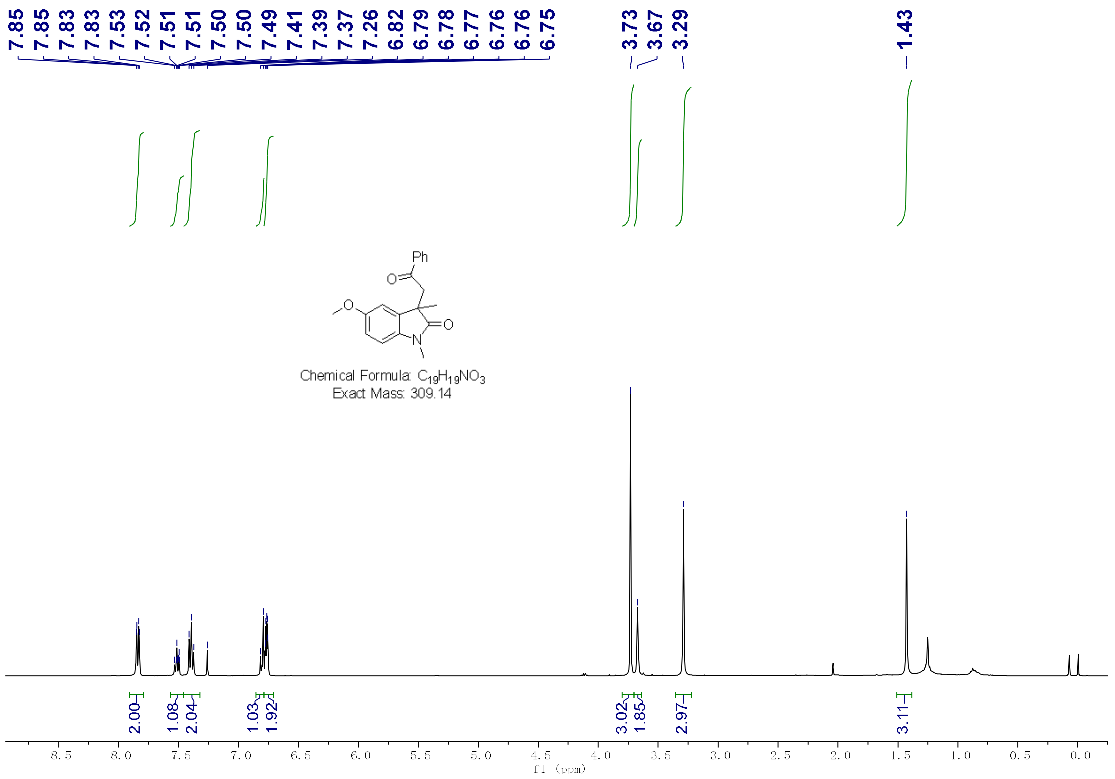

7.85
7.85
7.83
7.83
7.53
7.52
7.51
7.51
7.50
7.50
7.49
7.41
7.39
7.37
7.26
6.82
6.79
6.78
6.77
6.76
6.76
6.75
3.73
3.67
3.29
1.43
Ph
O
N
Chemical Formula: $C_{19}H_{19}NO_3$
Exact Mass: 309.14
2.00
1.08
2.04
1.03
1.92
3.02
1.85
2.97
3.11
f1 (ppm)

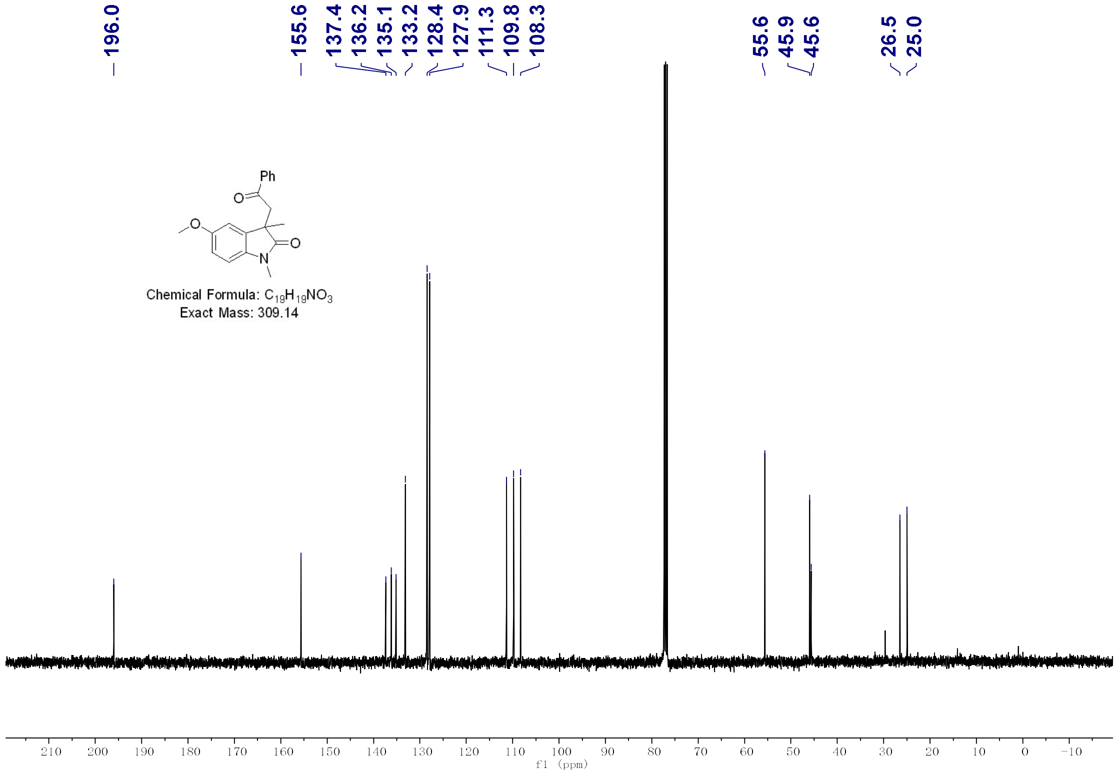

196.0
155.6
137.4
136.2
135.1
133.2
128.4
127.9
111.3
109.8
108.3
55.6
45.9
45.6
26.5
25.0
Ph
O
N
Chemical Formula: $C_{19}H_{19}NO_3$
Exact Mass: 309.14
f1 (ppm)

3c

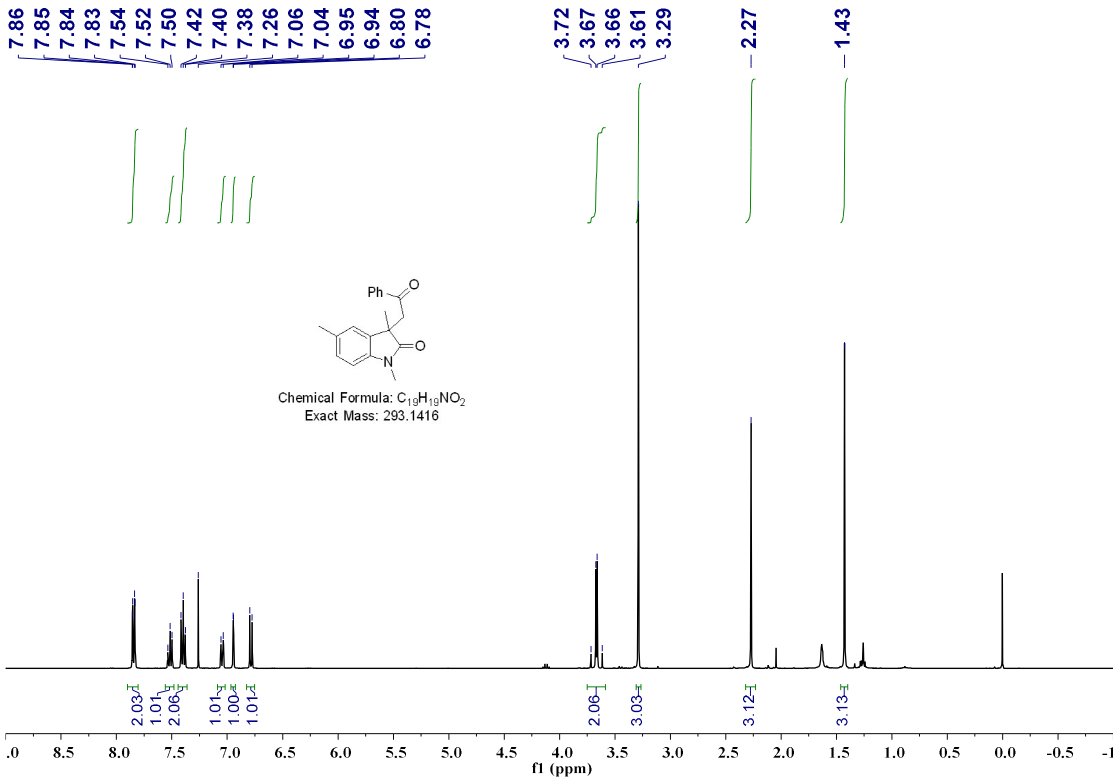
7.86
7.85
7.84
7.83
7.54
7.52
7.50
7.42
7.40
7.38
7.26
7.06
7.04
6.95
6.94
6.80
6.78
3.72
3.67
3.66
3.61
3.29
2.27
1.43
Chemical Formula: $C_{19}H_{19}NO_2$
Exact Mass: 293.1416
2.03
1.01
2.06
1.01
1.00
1.01
2.06
3.03
3.12
3.13
f1 (ppm)

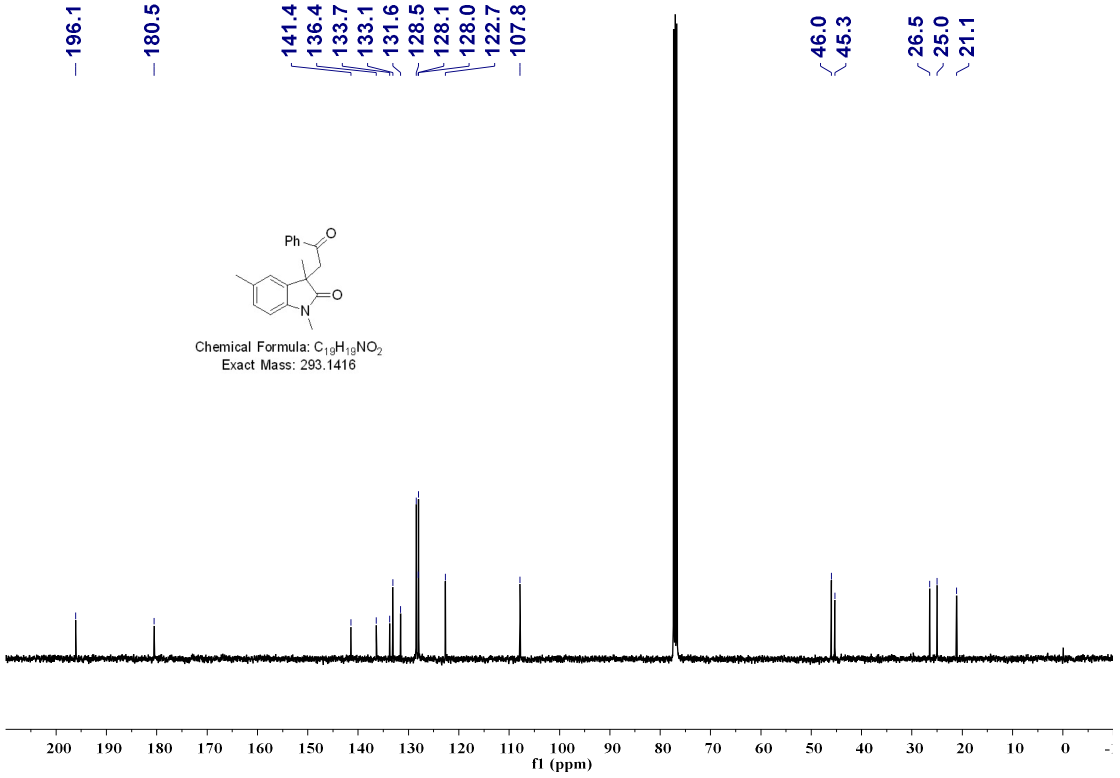
196.1
180.5
141.4
136.4
133.7
133.1
131.6
128.5
128.1
128.0
122.7
107.8
46.0
45.3
26.5
25.0
21.1
Chemical Formula: $C_{19}H_{19}NO_2$
Exact Mass: 293.1416
f1 (ppm)

3d

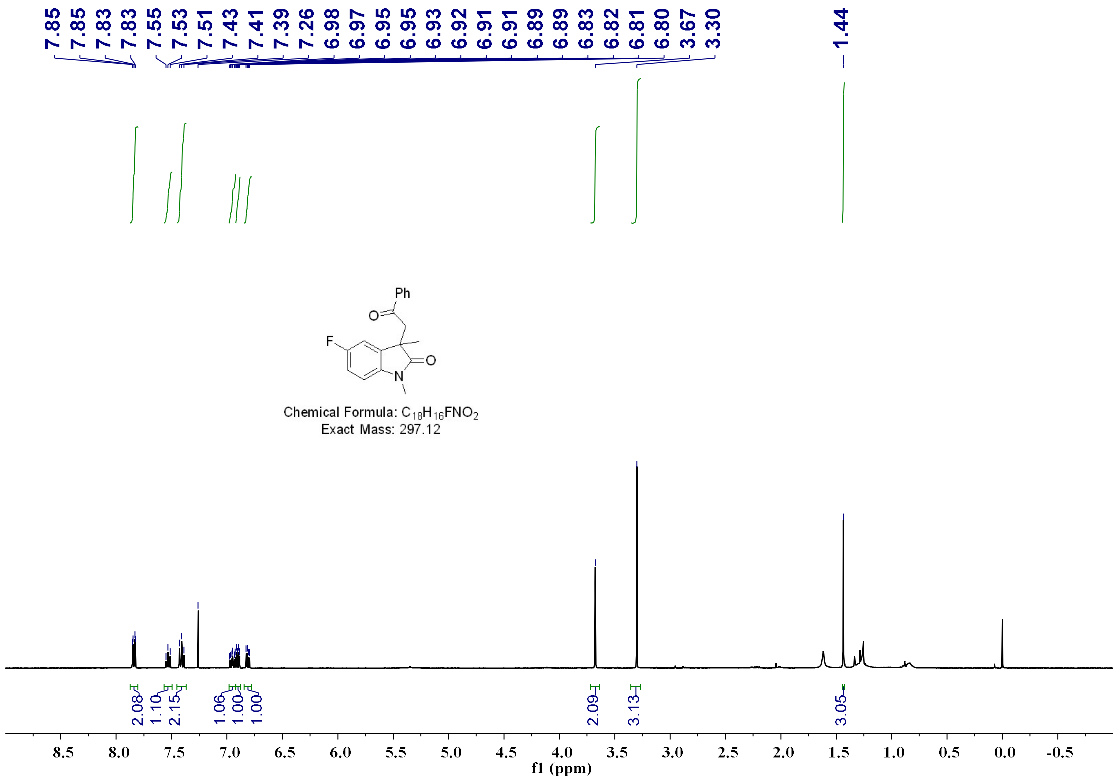
7.85
7.85
7.83
7.83
7.55
7.53
7.51
7.43
7.41
7.39
7.26
6.98
6.97
6.95
6.95
6.93
6.92
6.91
6.91
6.89
6.89
6.83
6.82
6.81
6.80
3.67
3.30
1.44
Ph
O
F
N
O
Chemical Formula: $C_{18}H_{16}FNO_2$
Exact Mass: 297.12
2.08
1.10
2.15
1.06
1.00
1.00
2.09
3.13
3.05
8.5 8.0 7.5 7.0 6.5 6.0 5.5 5.0 4.5 4.0 3.5 3.0 2.5 2.0 1.5 1.0 0.5 0.0 -0.5
f1 (ppm)

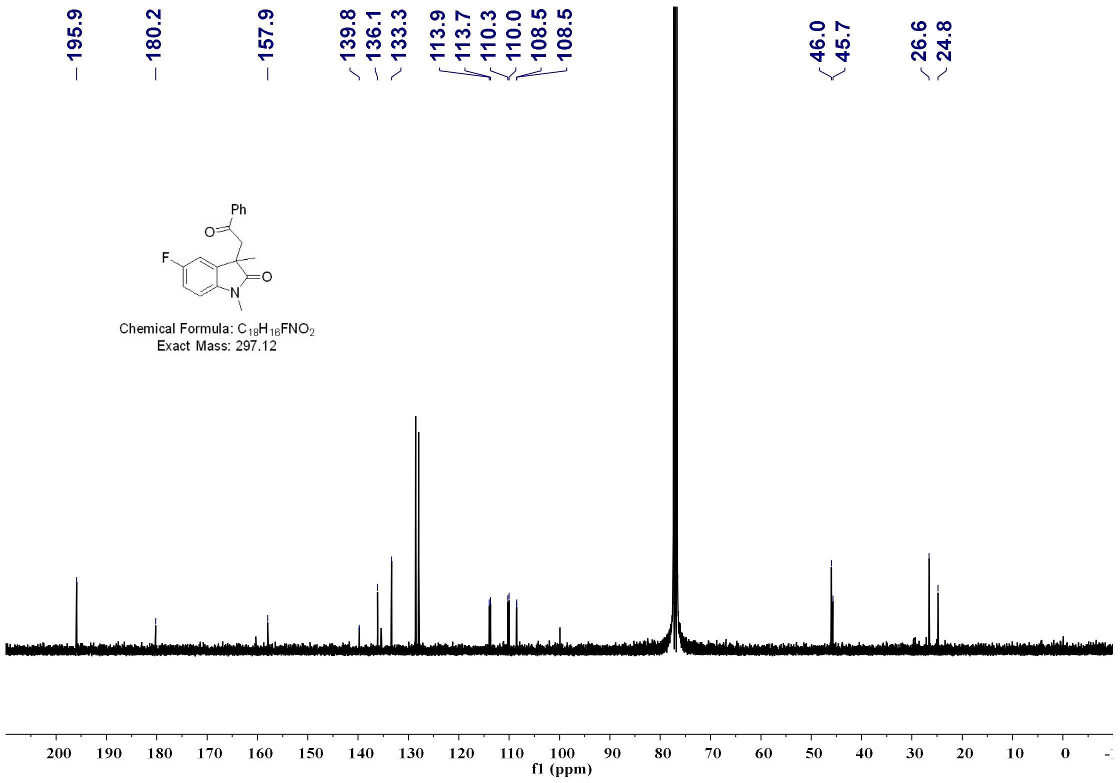
195.9
180.2
157.9
139.8
136.1
133.3
113.9
113.7
110.3
110.0
108.5
108.5
46.0
45.7
26.6
24.8
Ph
O
F
N
O
Chemical Formula: $C_{18}H_{16}FNO_2$
Exact Mass: 297.12
200 190 180 170 160 150 140 130 120 110 100 90 80 70 60 50 40 30 20 10 0
f1 (ppm)

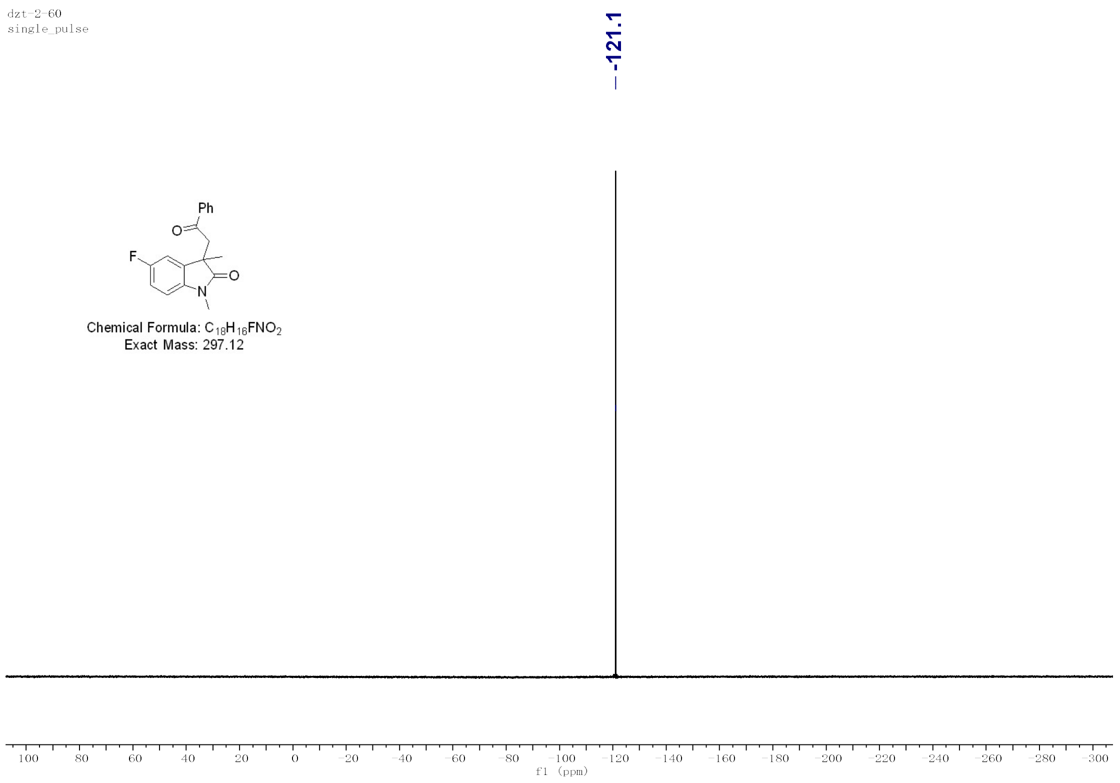
dzt-2-60
single_pulse
-121.1
Ph
O
F
O
N
Chemical Formula: $C_{18}H_{16}FNO_2$
Exact Mass: 297.12
100 80 60 40 20 0 -20 -40 -60 -80 -100 -120 -140 -160 -180 -200 -220 -240 -260 -280 -300
f1 (ppm)

3e

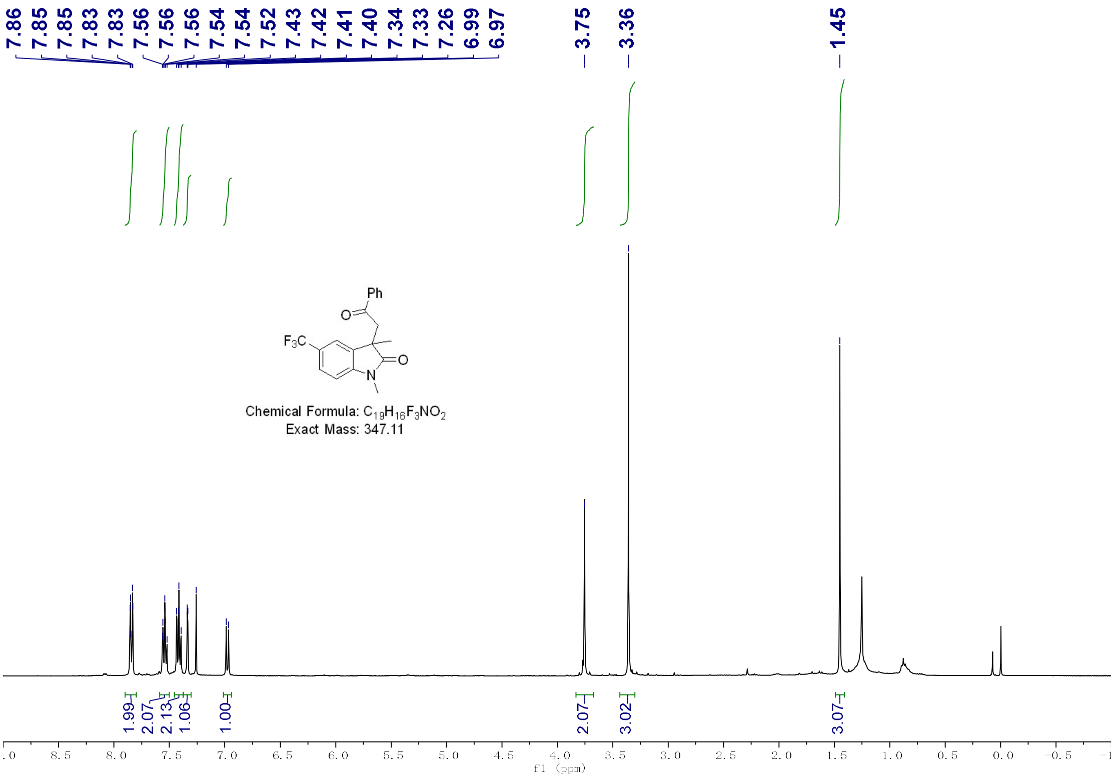

7.86
7.85
7.85
7.83
7.83
7.56
7.56
7.56
7.54
7.54
7.52
7.43
7.42
7.41
7.40
7.34
7.33
7.26
6.99
6.97
3.75
3.36
1.45
Ph
O
F3C
N
O
Chemical Formula: C19H16F3NO2
Exact Mass: 347.11
1.99
2.07
2.13
1.06
1.00
2.07
3.02
3.07
f1 (ppm)

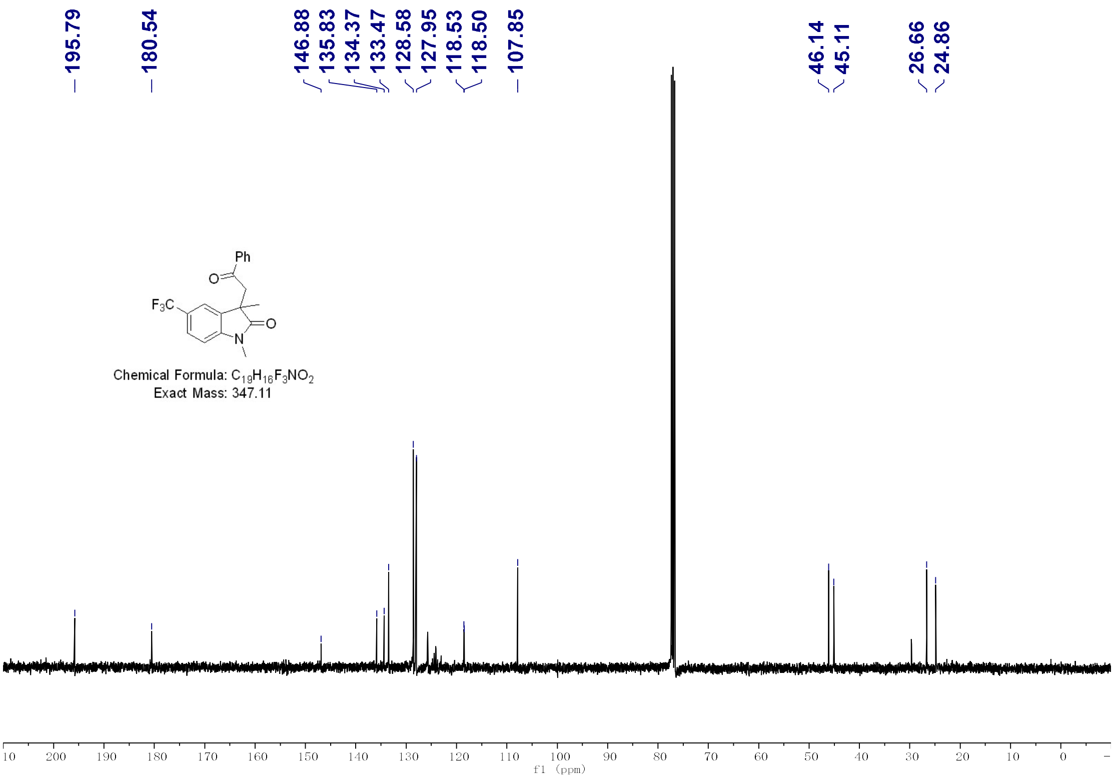

195.79
180.54
146.88
135.83
134.37
133.47
128.58
127.95
118.53
118.50
107.85
46.14
45.11
26.66
24.86
Ph
O
F3C
N
O
Chemical Formula: C19H16F3NO2
Exact Mass: 347.11
f1 (ppm)

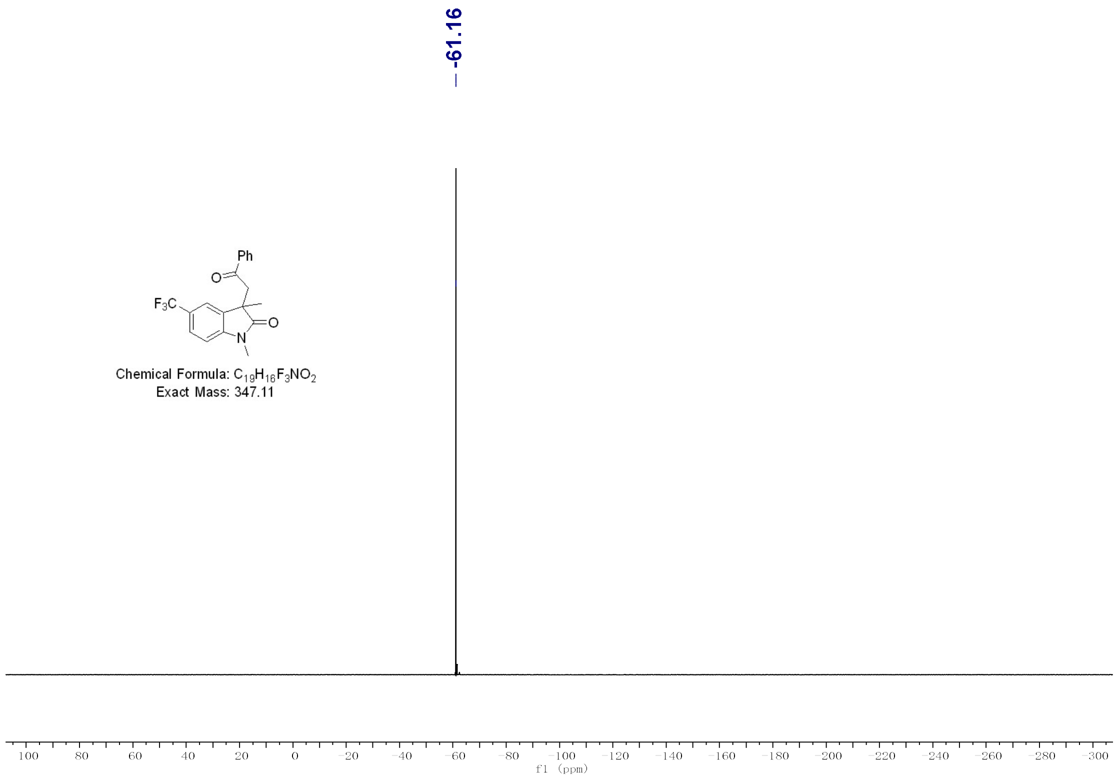
-61.16
Ph
O
F3C
O
N
Chemical Formula: C19H16F3NO2
Exact Mass: 347.11
100 80 60 40 20 0 -20 -40 -60 -80 -100 -120 -140 -160 -180 -200 -220 -240 -260 -280 -300
f1 (ppm)

3f

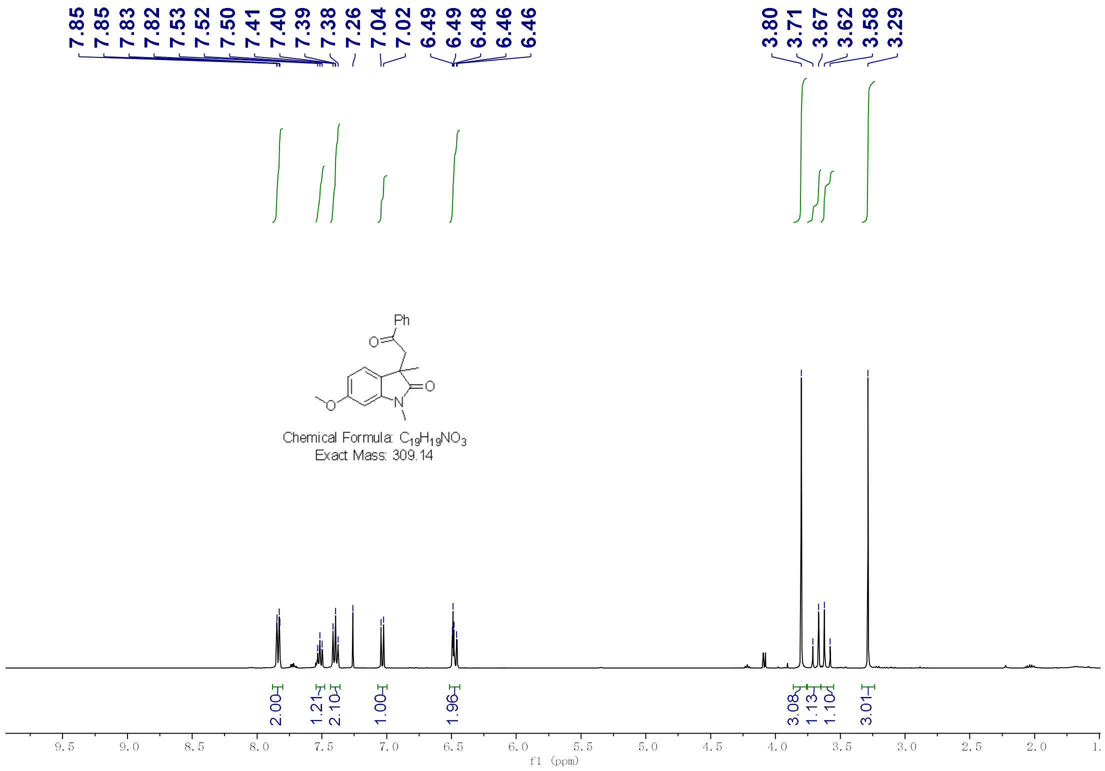

7.85 7.85 7.83 7.82 7.53 7.52 7.50 7.41 7.40 7.39 7.38 7.26 7.04 7.02 6.49 6.49 6.48 6.46 6.46
3.80 3.71 3.67 3.62 3.58 3.29
Ph
O
O
N
O
Chemical Formula: $C_{19}H_{19}NO_3$
Exact Mass: 309.14
2.00 1.21 2.10 1.00 1.96 3.08 1.13 1.10 3.01
9.5 9.0 8.5 8.0 7.5 7.0 6.5 6.0 5.5 5.0 4.5 4.0 3.5 3.0 2.5 2.0 1.
f1 (ppm)

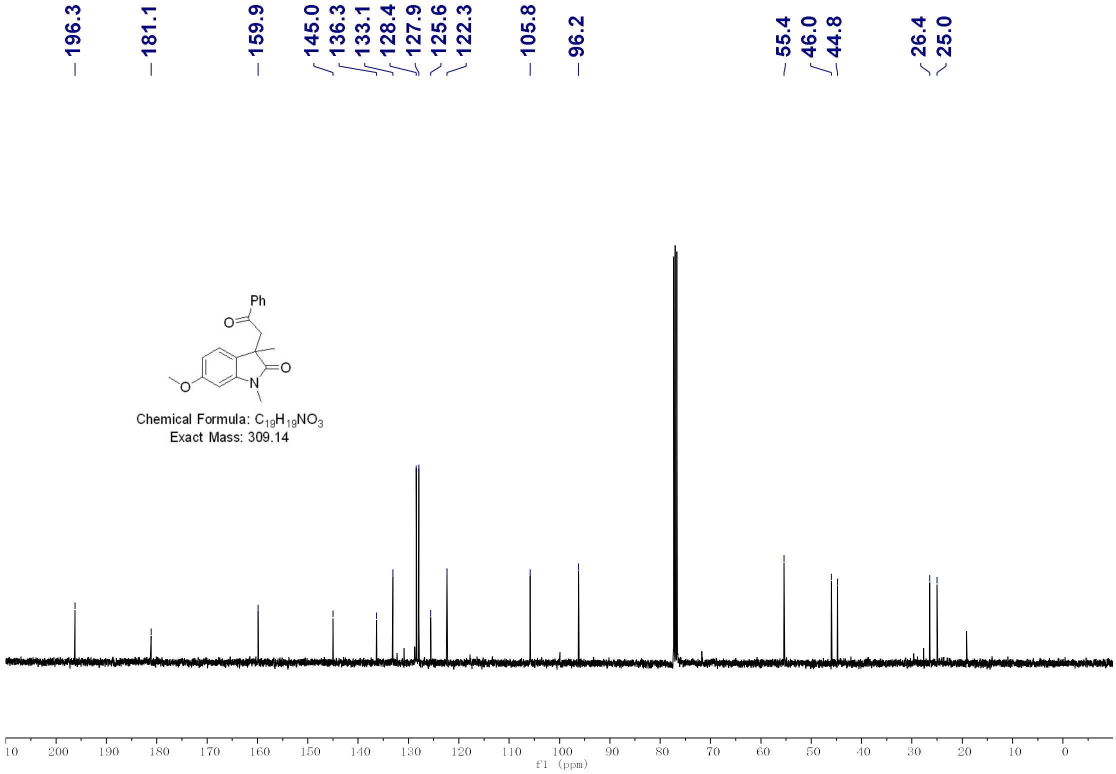

196.3 181.1 159.9 145.0 136.3 133.1 128.4 127.9 125.6 122.3 105.8 96.2 55.4 46.0 44.8 26.4 25.0
Ph
O
O
N
O
Chemical Formula: $C_{18}H_{19}NO_3$
Exact Mass: 309.14
10 200 190 180 170 160 150 140 130 120 110 100 90 80 70 60 50 40 30 20 10 0
f1 (ppm)

3g

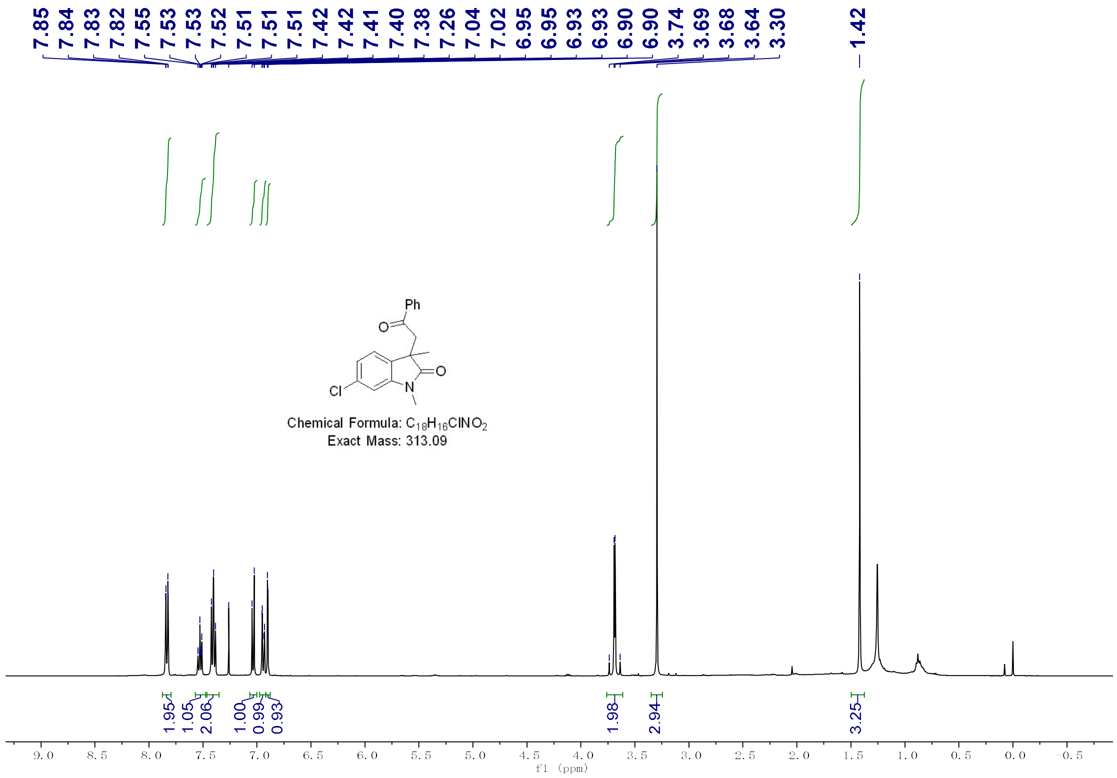
7.85
7.84
7.83
7.82
7.55
7.53
7.53
7.52
7.51
7.51
7.51
7.42
7.42
7.41
7.40
7.38
7.26
7.04
7.02
6.95
6.95
6.93
6.93
6.90
6.90
3.74
3.69
3.68
3.64
3.30
1.42
Ph
O
Cl
N
O
Chemical Formula: C18H16ClNO2
Exact Mass: 313.09
1.95
1.05
2.06
1.00
0.99
0.93
1.98
2.94
3.25
f1 (ppm)

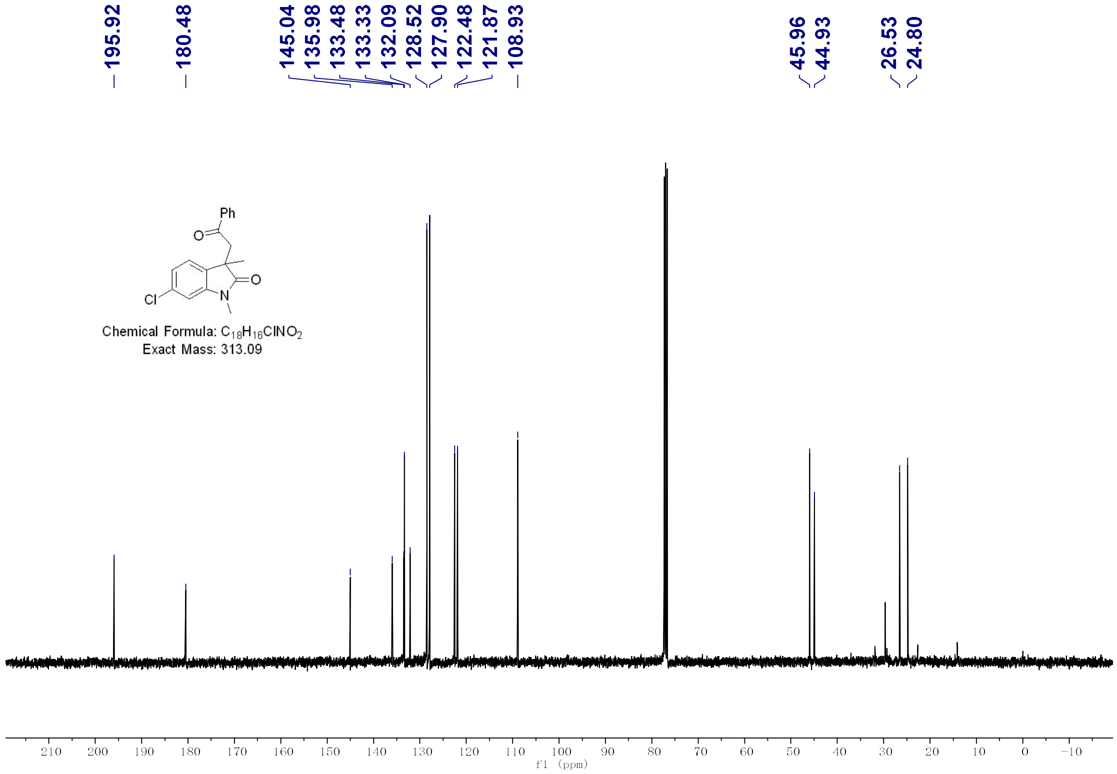
195.92
180.48
145.04
135.98
133.48
133.33
132.09
128.52
127.90
122.48
121.87
108.93
45.96
44.93
26.53
24.80
Ph
O
Cl
N
O
Chemical Formula: C18H16ClNO2
Exact Mass: 313.09
f1 (ppm)

3h

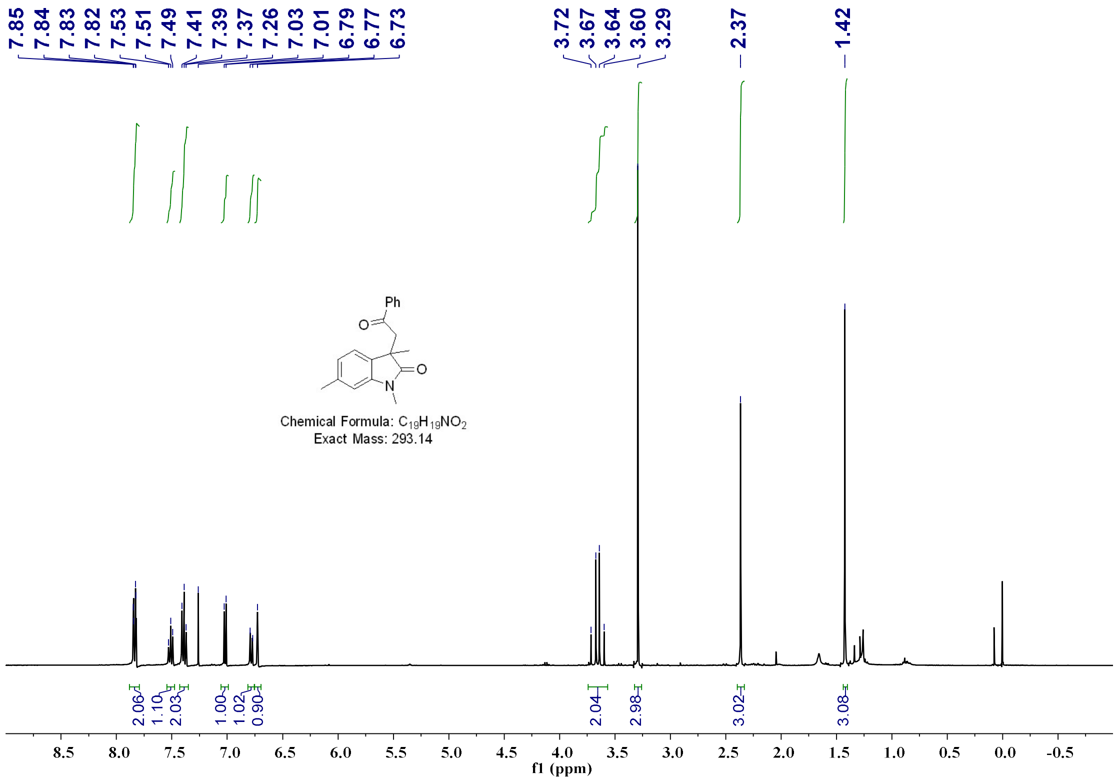
7.85
7.84
7.83
7.82
7.53
7.51
7.49
7.41
7.39
7.37
7.26
7.03
7.01
6.79
6.77
6.73
3.72
3.67
3.64
3.60
3.29
2.37
1.42
Ph
O
O
N
Chemical Formula: C19H19NO2
Exact Mass: 293.14
2.06
1.10
2.03
1.00
1.02
0.90
2.04
2.98
3.02
3.08
f1 (ppm)

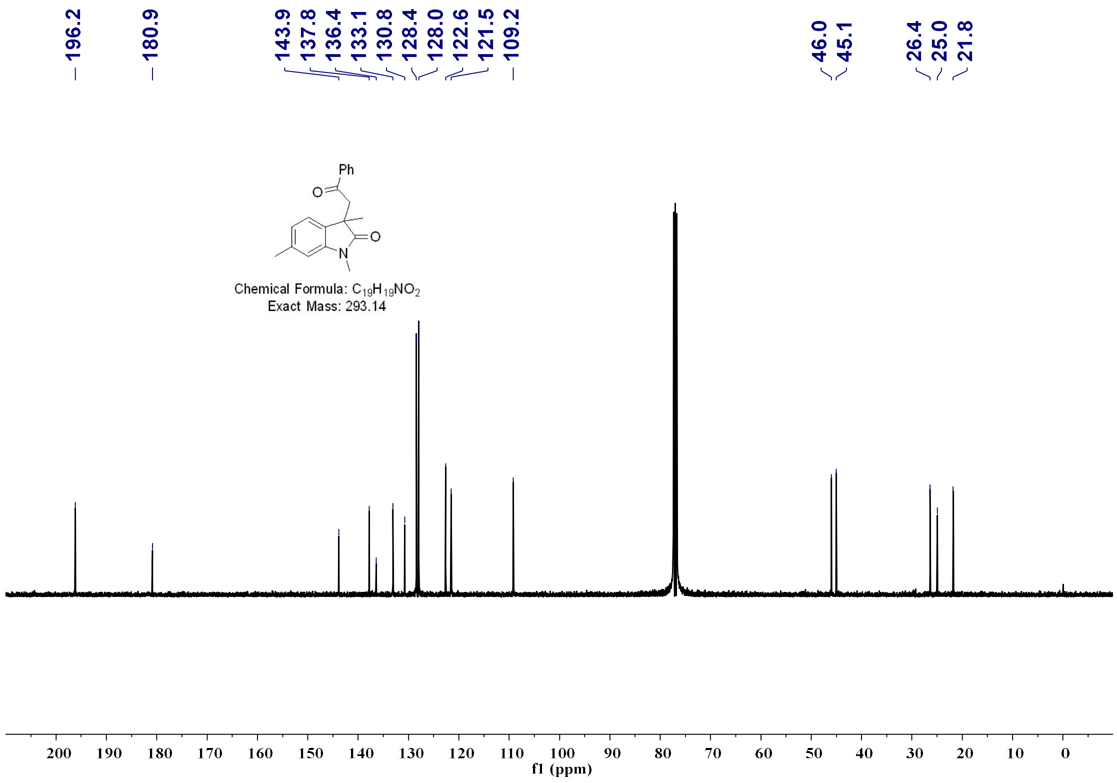
196.2
180.9
143.9
137.8
136.4
133.1
130.8
128.4
128.0
122.6
121.5
109.2
46.0
45.1
26.4
25.0
21.8
Ph
O
O
N
Chemical Formula: C19H19NO2
Exact Mass: 293.14
f1 (ppm)

3i

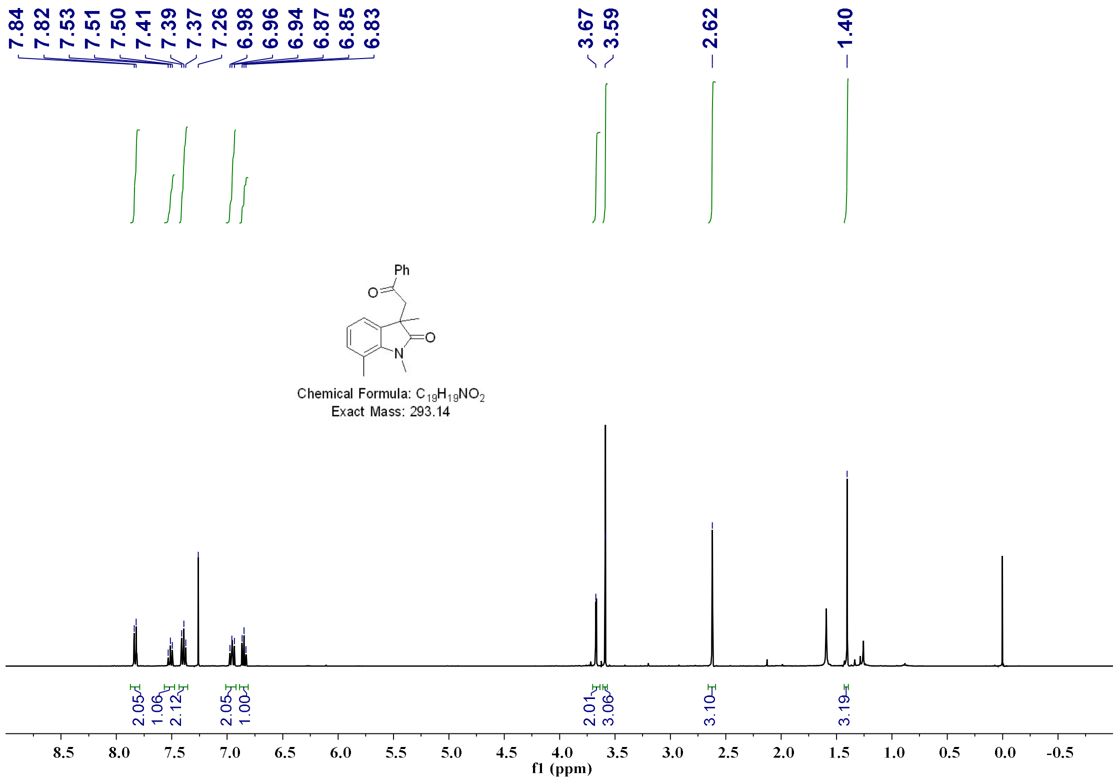
7.84
7.82
7.53
7.51
7.50
7.41
7.39
7.37
7.26
6.98
6.96
6.94
6.87
6.85
6.83
3.67
3.59
2.62
1.40
Ph
O
N
O
Chemical Formula: C19H19NO2
Exact Mass: 293.14
2.05
1.06
2.12
2.05
1.00
2.01
3.06
3.10
3.19
8.5
8.0
7.5
7.0
6.5
6.0
5.5
5.0
4.5
4.0
3.5
3.0
2.5
2.0
1.5
1.0
0.5
0.0
-0.5
f1 (ppm)

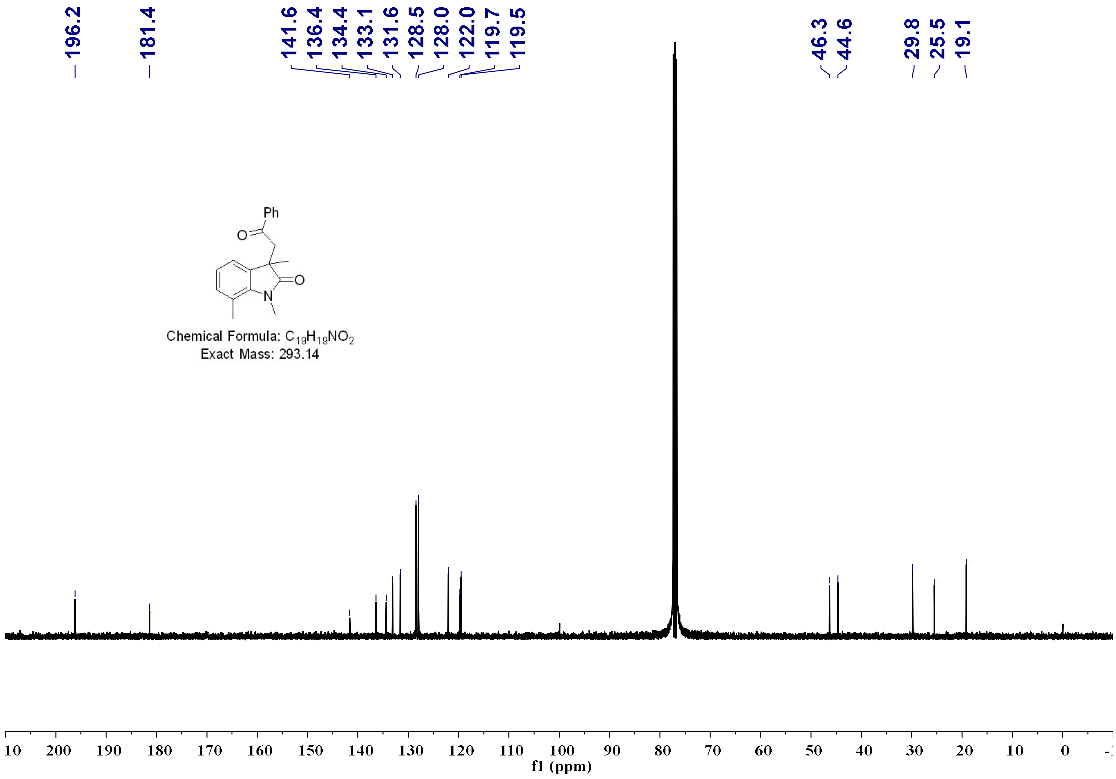
196.2
181.4
141.6
136.4
134.4
133.1
131.6
128.5
128.0
122.0
119.7
119.5
46.3
44.6
29.8
25.5
19.1
Ph
O
N
O
Chemical Formula: C19H19NO2
Exact Mass: 293.14
10
200
190
180
170
160
150
140
130
120
110
100
90
80
70
60
50
40
30
20
10
0
f1 (ppm)

3j

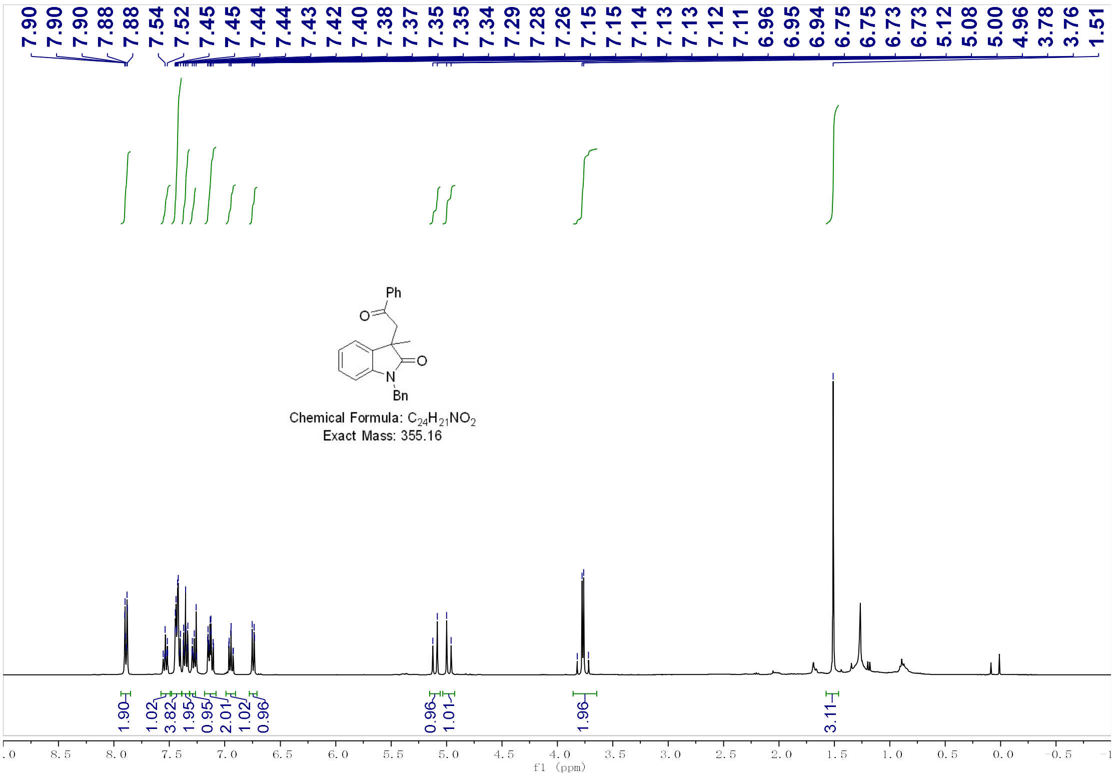

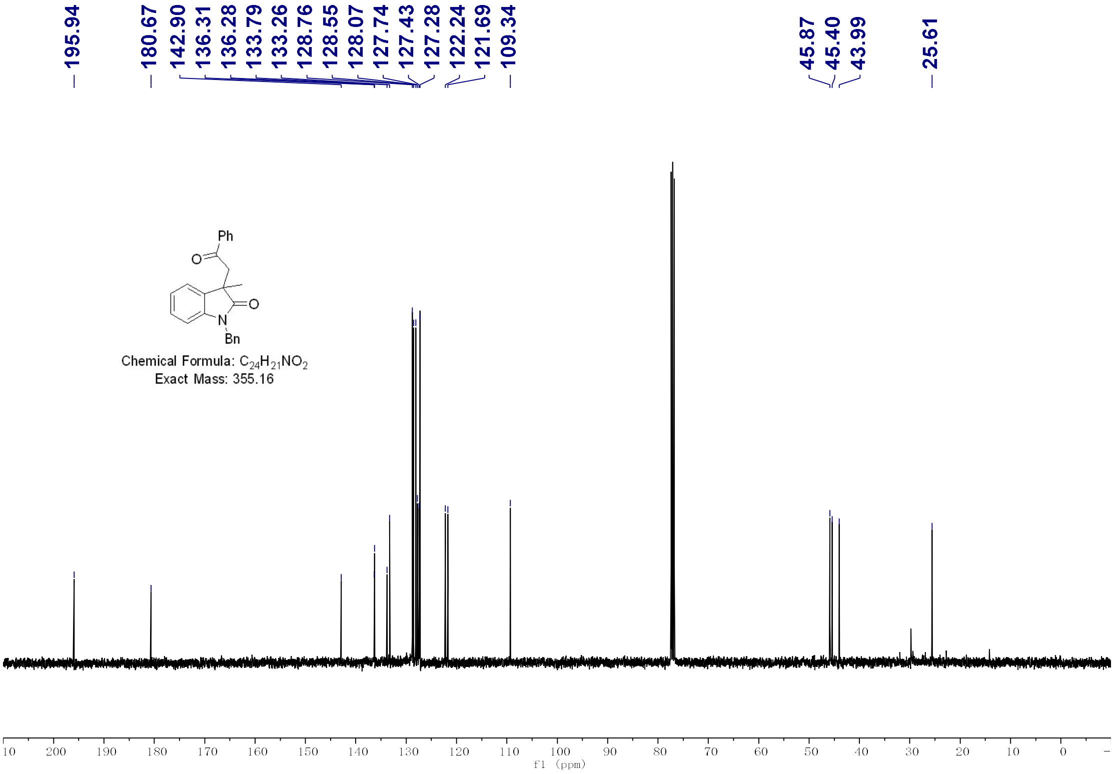

3k

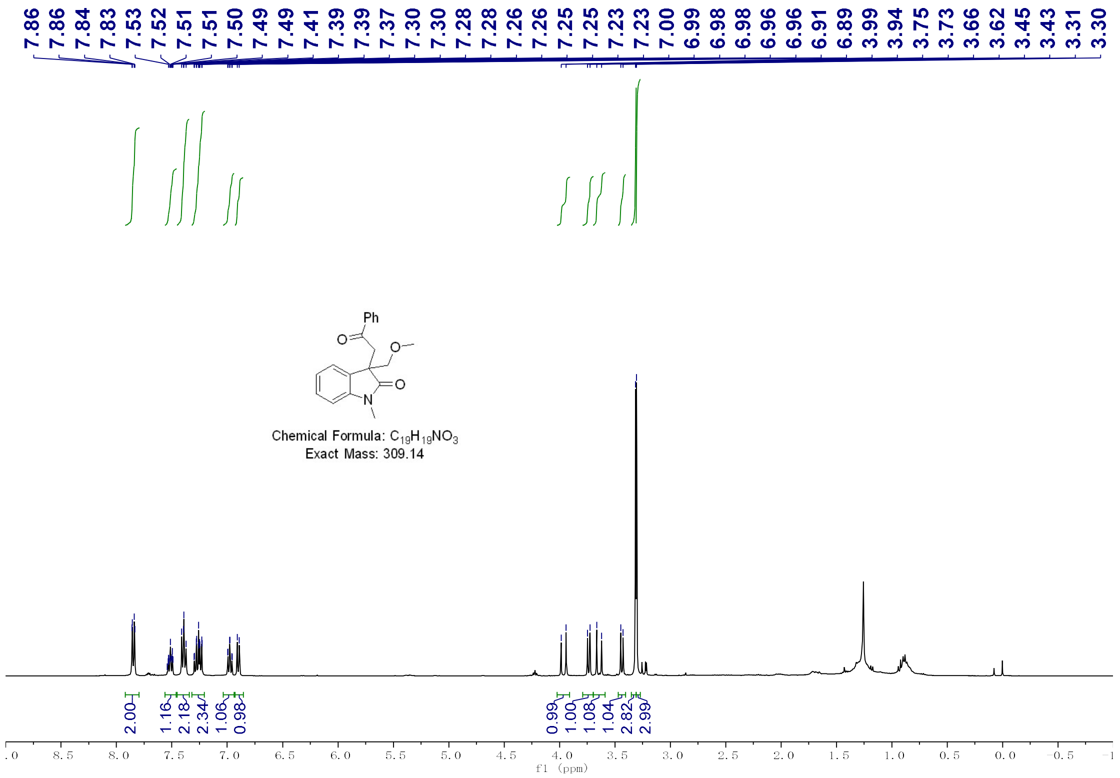

7.86 7.86 7.84 7.83 7.53 7.52 7.51 7.51 7.50 7.49 7.41 7.39 7.39 7.37 7.30 7.30 7.28 7.28 7.26 7.26 7.25 7.25 7.23 7.23 7.00 6.99 6.98 6.98 6.96 6.96 6.91 6.89 3.99 3.94 3.75 3.73 3.66 3.62 3.45 3.43 3.31 3.30
Ph
O
N
Chemical Formula: $C_{19}H_{19}NO_3$
Exact Mass: 309.14
2.00 1.16 2.18 2.34 1.06 0.98 0.99 1.00 1.08 1.04 2.82 2.99
f1 (ppm)

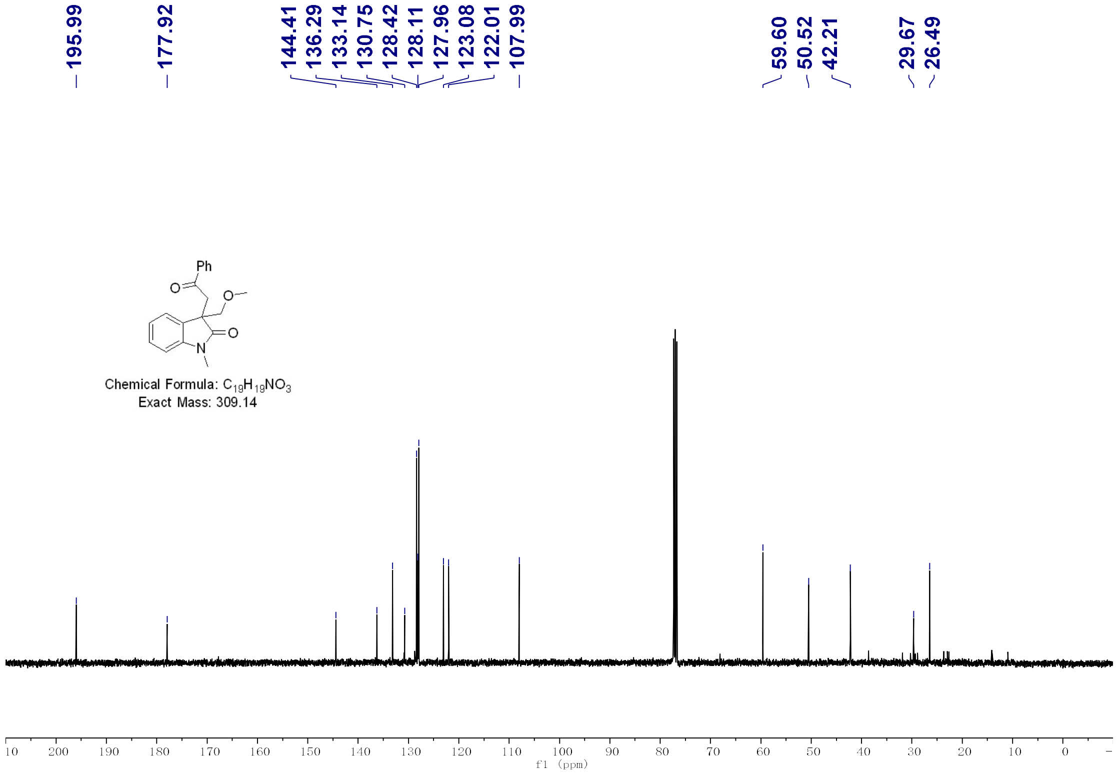

195.99 177.92 144.41 136.29 133.14 130.75 128.42 128.11 127.96 123.08 122.01 107.99 59.60 50.52 42.21 29.67 26.49
Ph
O
N
Chemical Formula: $C_{19}H_{19}NO_3$
Exact Mass: 309.14
f1 (ppm)

3l

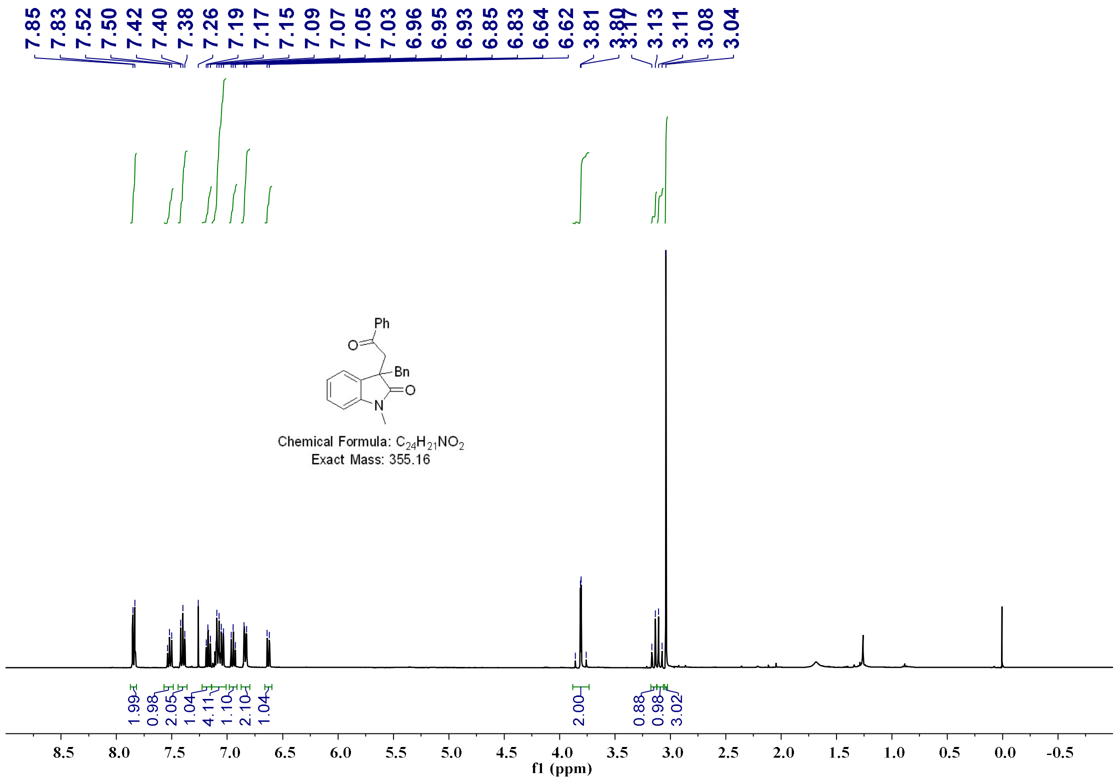

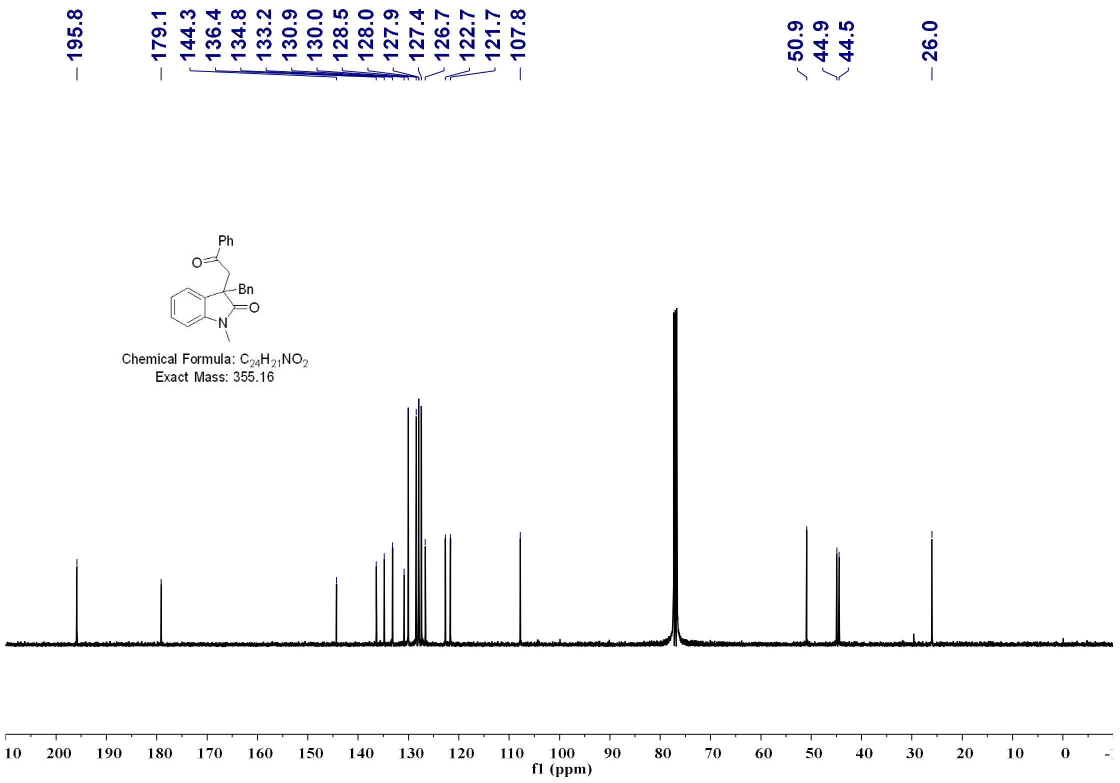

3m

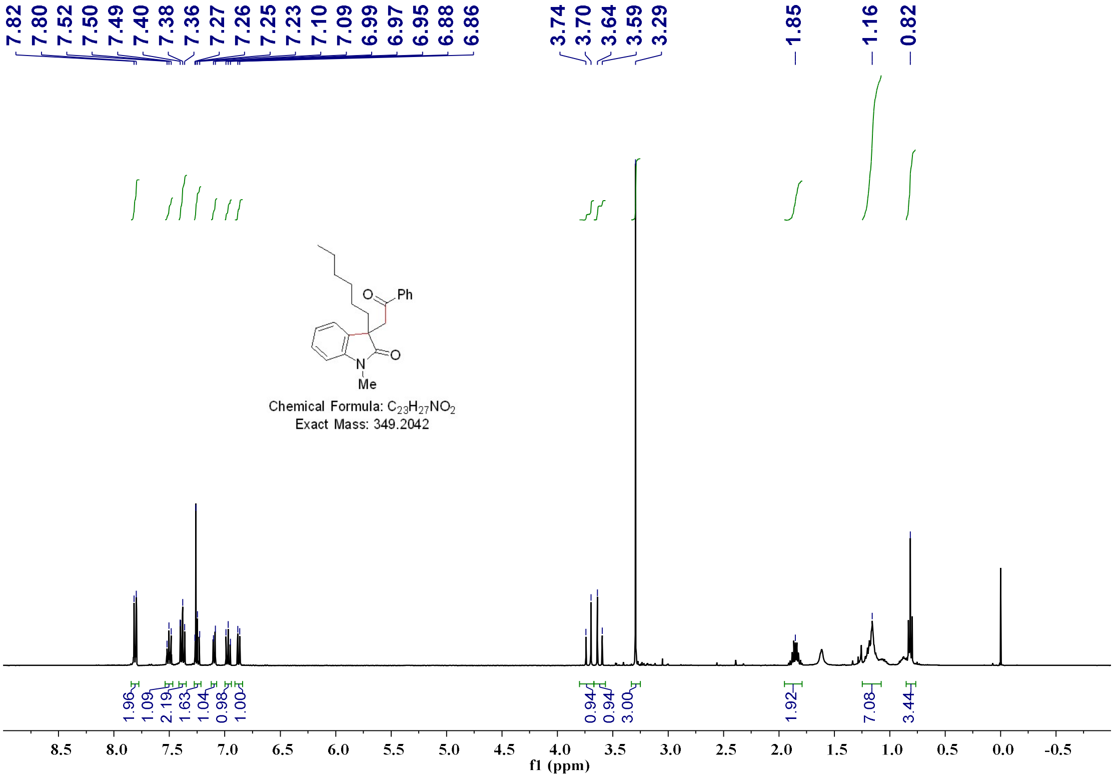
7.82 7.80 7.52 7.50 7.49 7.40 7.38 7.36 7.27 7.26 7.25 7.23 7.10 7.09 6.99 6.97 6.95 6.88 6.86
3.74 3.70 3.64 3.59 3.29
1.85
1.16
0.82
O
Ph
N
O
Me
Chemical Formula: C23H27NO2
Exact Mass: 349.2042
1.96 1.09 2.19 1.63 1.04 0.98 1.00
0.94 0.94 3.00
1.92
7.08
3.44
8.5 8.0 7.5 7.0 6.5 6.0 5.5 5.0 4.5 4.0 3.5 3.0 2.5 2.0 1.5 1.0 0.5 0.0 -0.5
f1 (ppm)

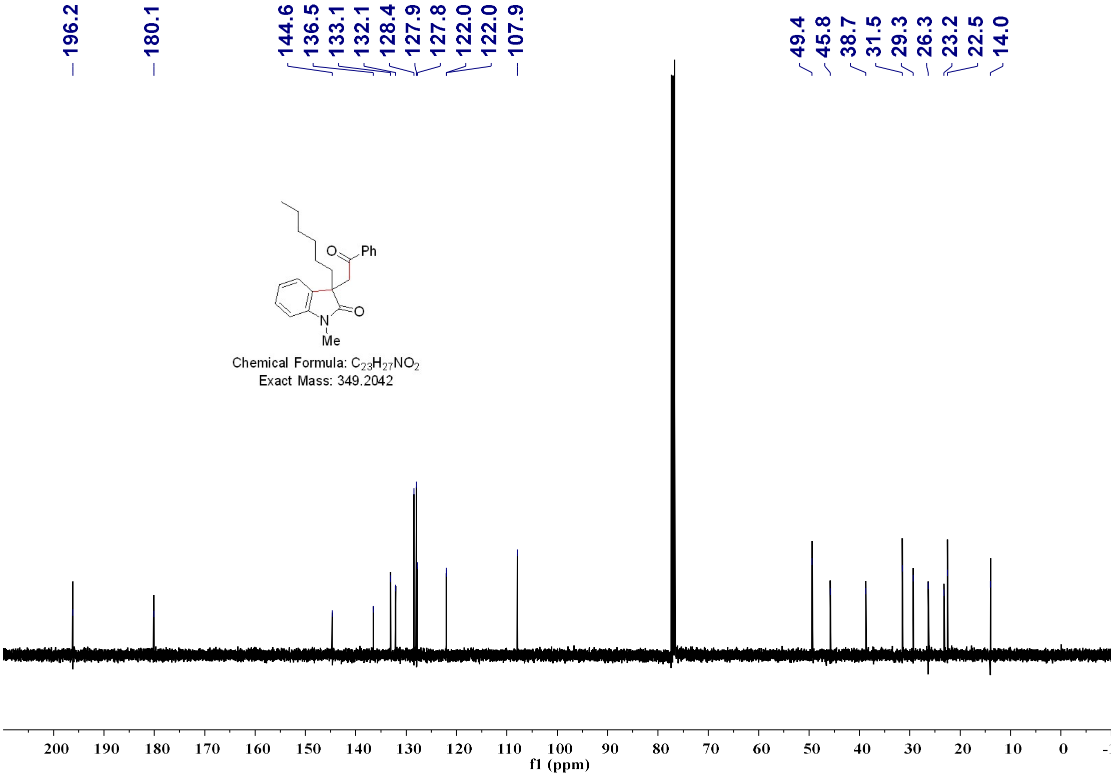
196.2
180.1
144.6 136.5 133.1 132.1 128.4 127.9 127.8 122.0 122.0 107.9
49.4 45.8 38.7 31.5 29.3 26.3 23.2 22.5 14.0
O
Ph
N
O
Me
Chemical Formula: C23H27NO2
Exact Mass: 349.2042
200 190 180 170 160 150 140 130 120 110 100 90 80 70 60 50 40 30 20 10 0
f1 (ppm)

3n

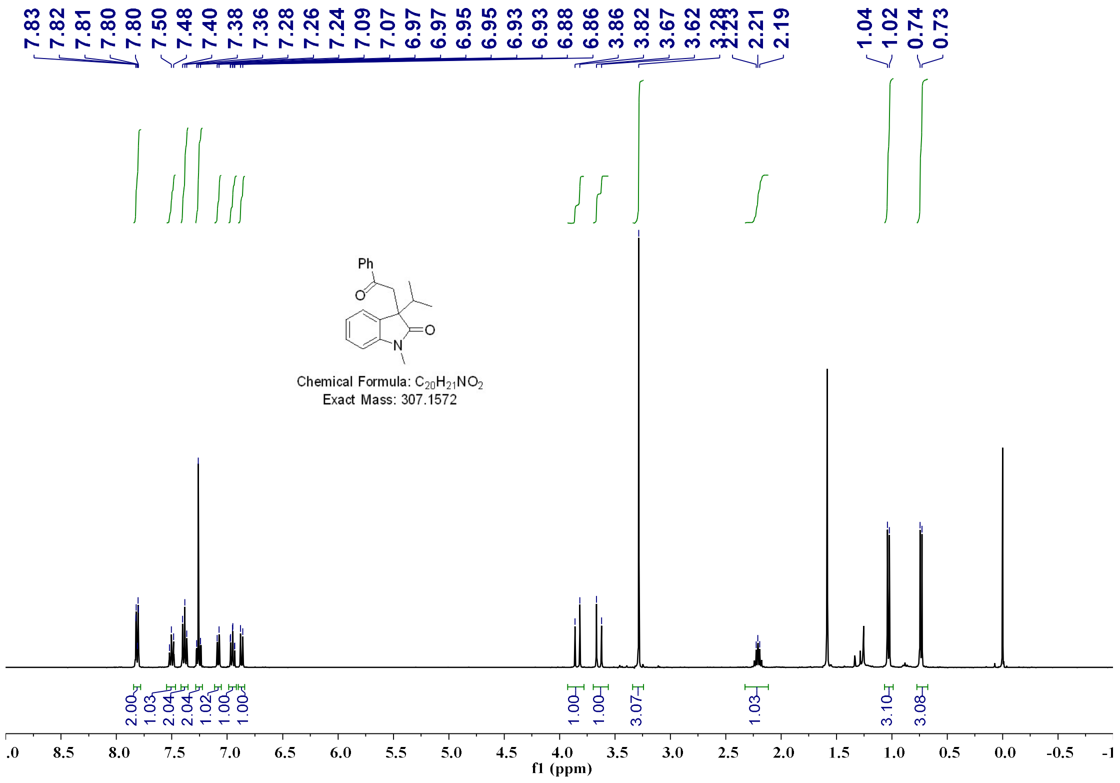
Chemical Formula: $C_{20}H_{21}NO_2$
Exact Mass: 307.1572
f1 (ppm)

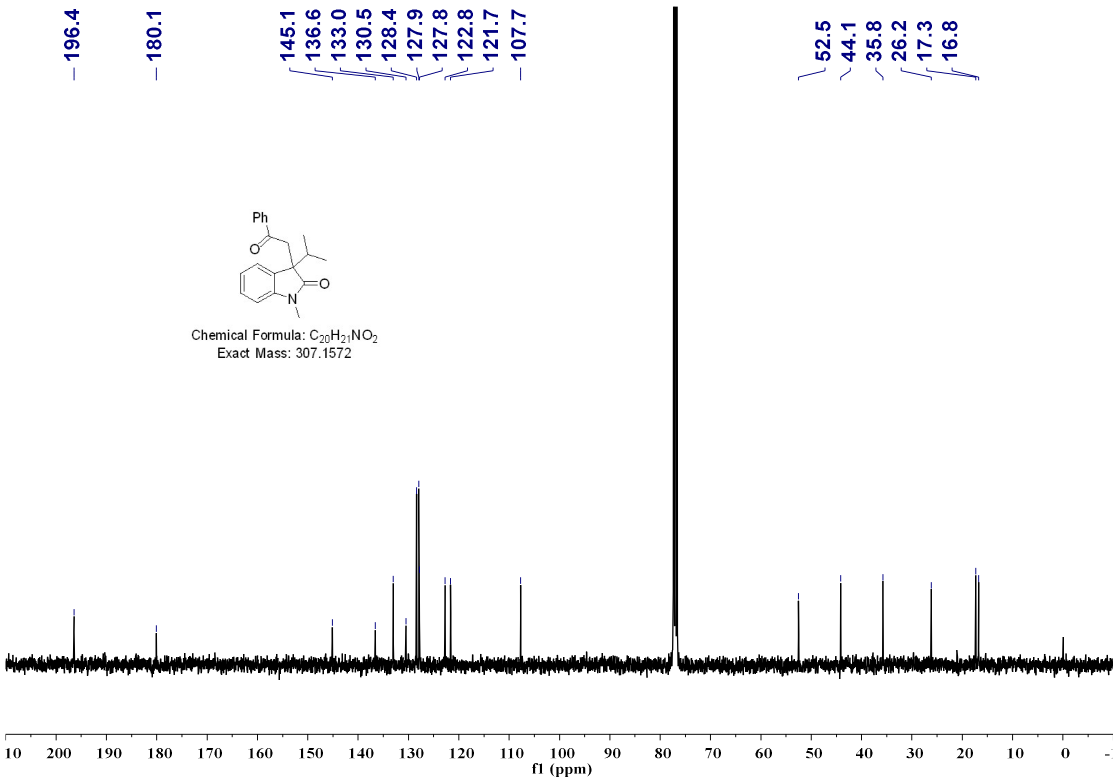
Chemical Formula: $C_{20}H_{21}NO_2$
Exact Mass: 307.1572
f1 (ppm)

3o

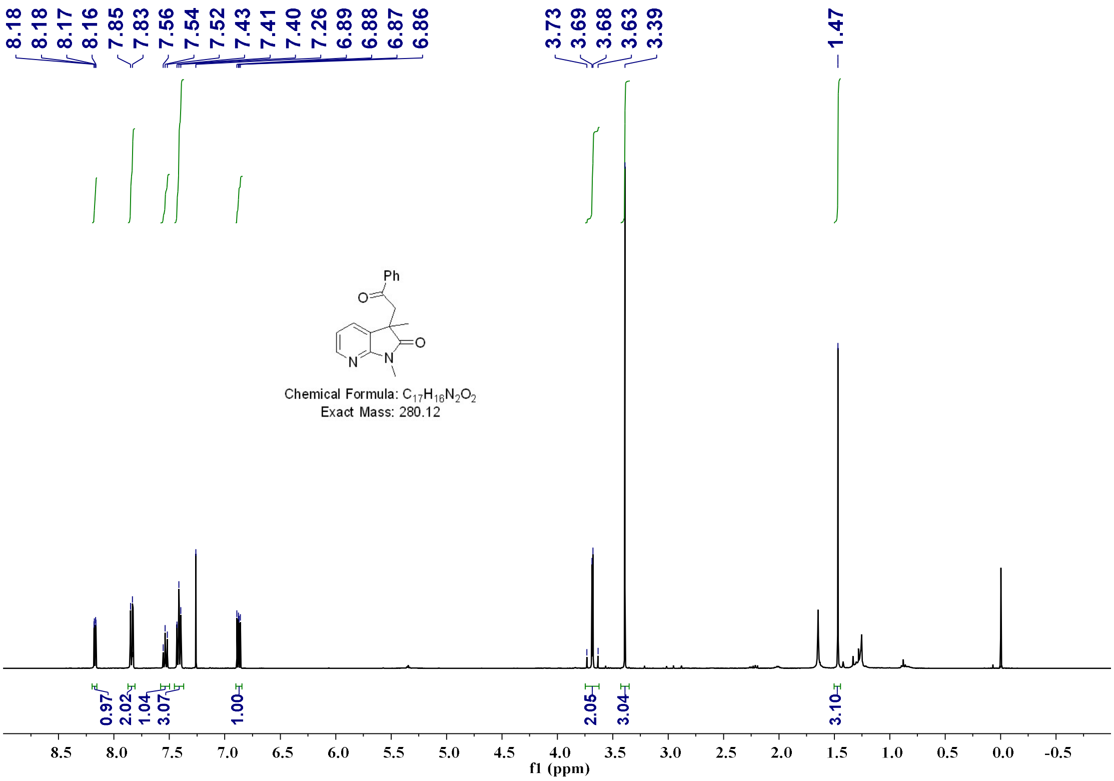

8.18
8.18
8.17
8.16
7.85
7.83
7.56
7.54
7.52
7.43
7.41
7.40
7.26
6.89
6.88
6.87
6.86
3.73
3.69
3.68
3.63
3.39
1.47
Ph
O
N
N
O
Chemical Formula: C17H16N2O2
Exact Mass: 280.12
0.97
2.02
1.04
3.07
1.00
2.05
3.04
3.10
f1 (ppm)

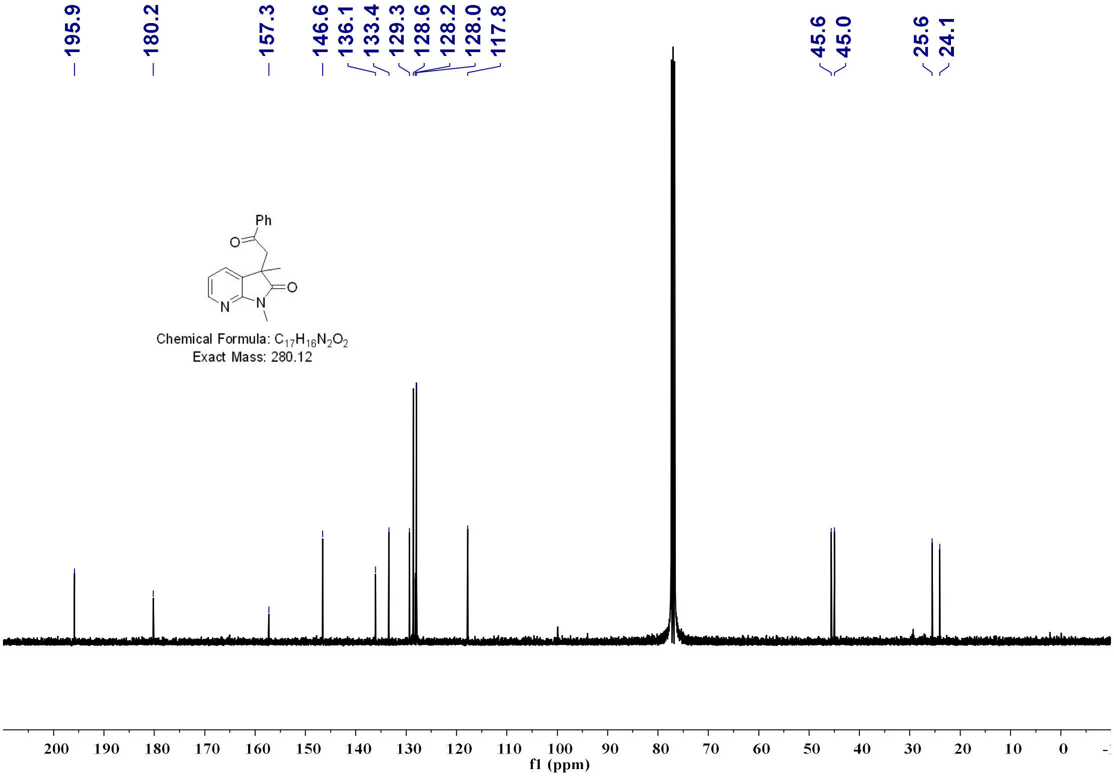

195.9
180.2
157.3
146.6
136.1
133.4
129.3
128.6
128.2
128.0
117.8
45.6
45.0
25.6
24.1
Ph
O
N
N
O
Chemical Formula: C17H16N2O2
Exact Mass: 280.12
f1 (ppm)

3p

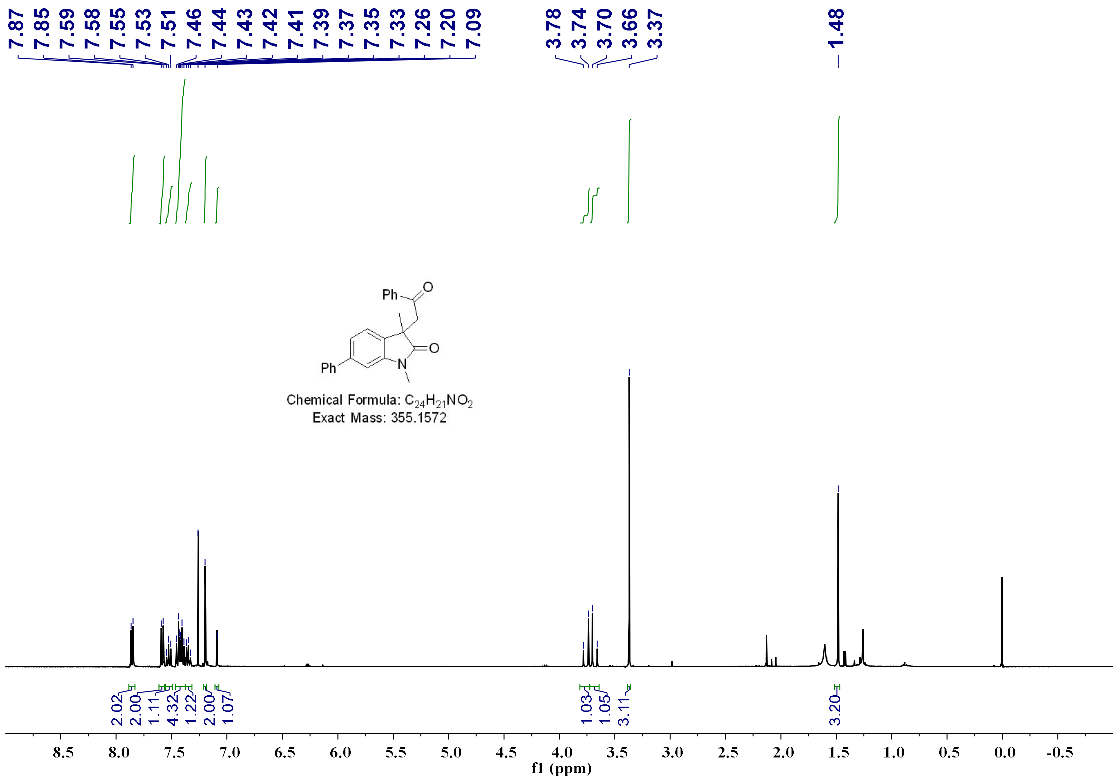
7.87
7.85
7.59
7.58
7.55
7.53
7.51
7.46
7.44
7.43
7.42
7.41
7.39
7.37
7.35
7.33
7.26
7.20
7.09
3.78
3.74
3.70
3.66
3.37
1.48
Ph
O
O
N
Ph
Chemical Formula: $C_{24}H_{21}NO_2$
Exact Mass: 355.1572
2.02
2.00
1.11
4.32
1.22
2.00
1.07
1.03
1.05
3.11
3.20
8.5 8.0 7.5 7.0 6.5 6.0 5.5 5.0 4.5 4.0 3.5 3.0 2.5 2.0 1.5 1.0 0.5 0.0 -0.5
f1 (ppm)

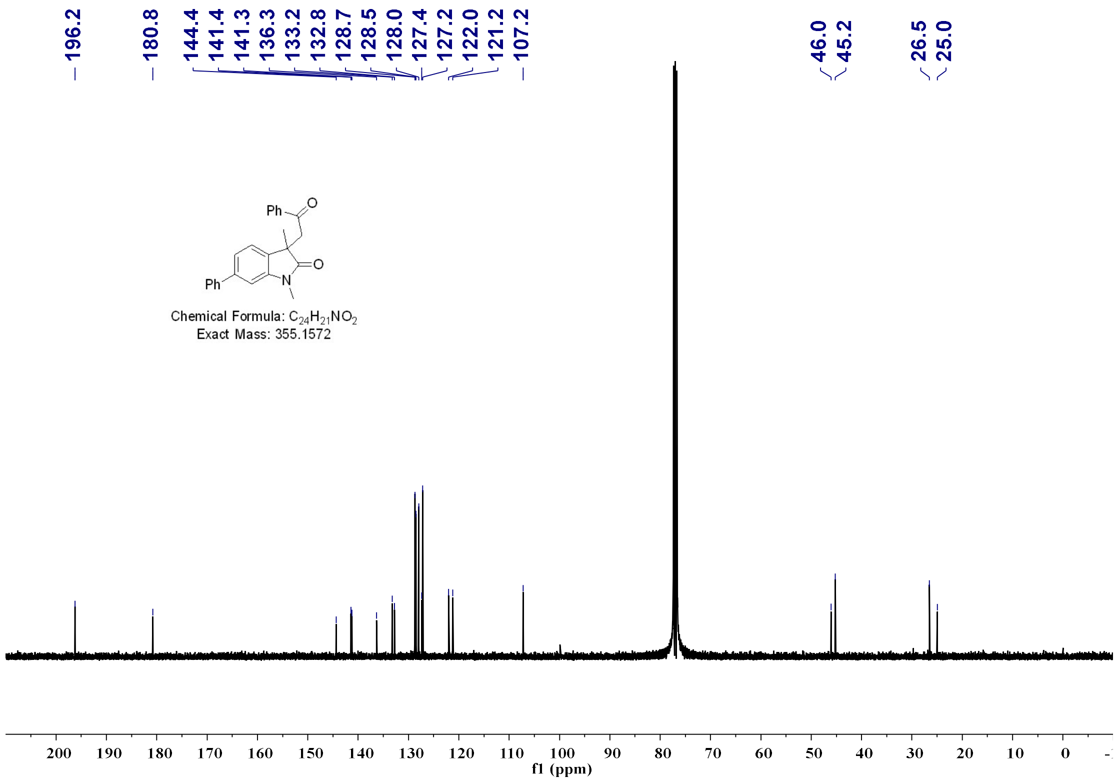
196.2
180.8
144.4
141.4
141.3
136.3
133.2
132.8
128.7
128.5
128.0
127.4
127.2
122.0
121.2
107.2
46.0
45.2
26.5
25.0
Ph
O
O
N
Ph
Chemical Formula: $C_{24}H_{21}NO_2$
Exact Mass: 355.1572
200 190 180 170 160 150 140 130 120 110 100 90 80 70 60 50 40 30 20 10 0
f1 (ppm)

3q

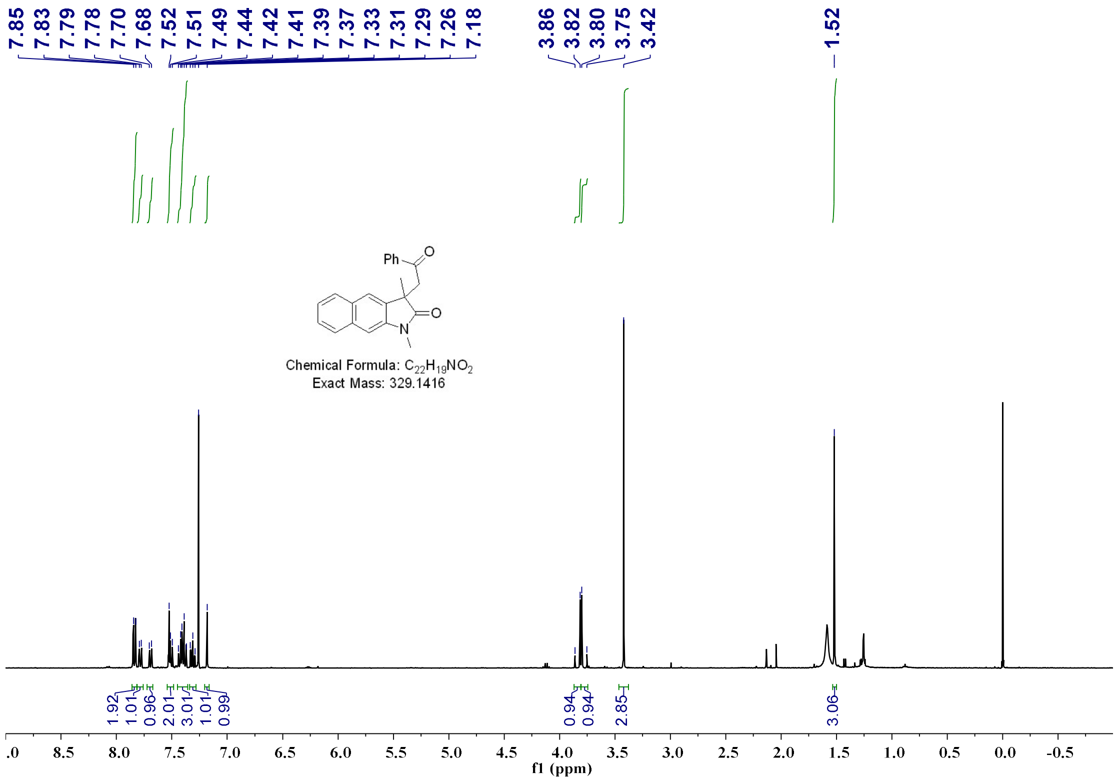

7.85
7.83
7.79
7.78
7.70
7.68
7.52
7.51
7.49
7.44
7.42
7.41
7.39
7.37
7.33
7.31
7.29
7.26
7.18
3.86
3.82
3.80
3.75
3.42
1.52
Ph
O
O
N
Chemical Formula: $C_{22}H_{19}NO_2$
Exact Mass: 329.1416
1.92
1.01
0.96
2.01
3.01
1.01
0.99
0.94
0.94
2.85
3.06
f1 (ppm)

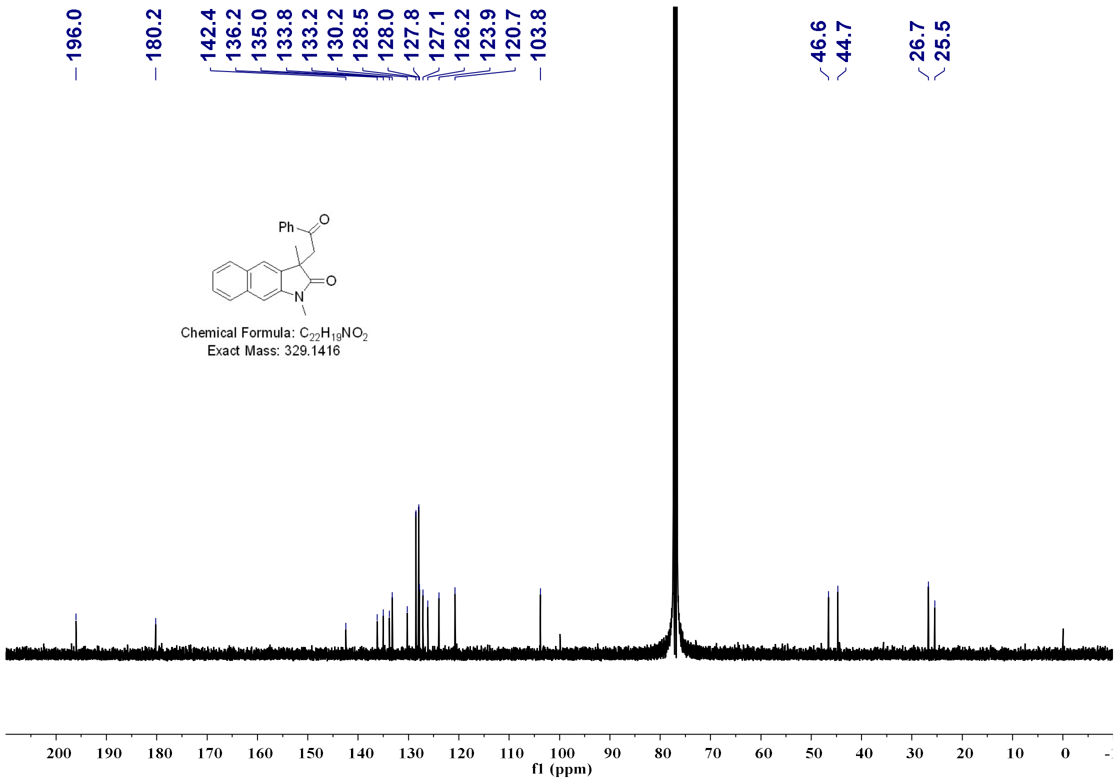

196.0
180.2
142.4
136.2
135.0
133.8
133.2
130.2
128.5
128.0
127.8
127.1
126.2
123.9
120.7
103.8
46.6
44.7
26.7
25.5
Ph
O
O
N
Chemical Formula: $C_{22}H_{19}NO_2$
Exact Mass: 329.1416
f1 (ppm)

3r

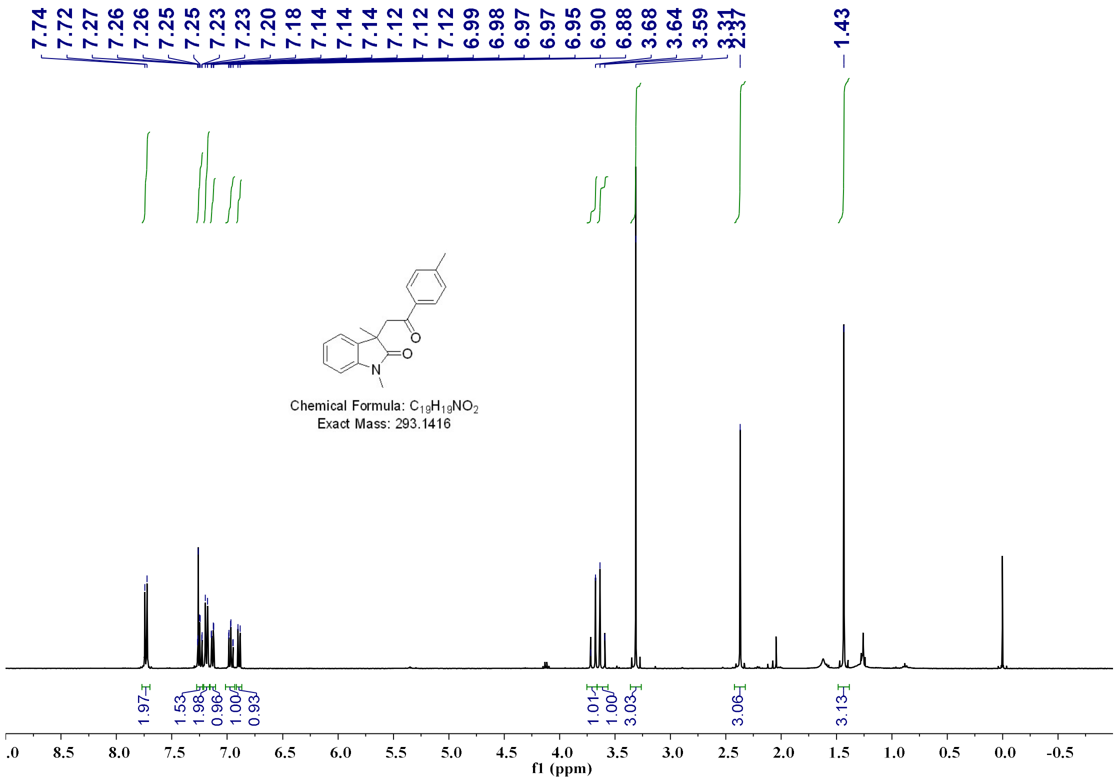
7.74
7.72
7.27
7.26
7.25
7.25
7.23
7.23
7.20
7.18
7.14
7.14
7.14
7.12
7.12
7.12
6.99
6.98
6.97
6.97
6.95
6.90
6.88
3.68
3.64
3.59
3.31
2.37
1.43
Chemical Formula: $C_{19}H_{19}NO_2$
Exact Mass: 293.1416
1.97
1.53
1.98
0.96
1.00
0.93
1.01
1.00
3.03
3.06
3.13
f1 (ppm)

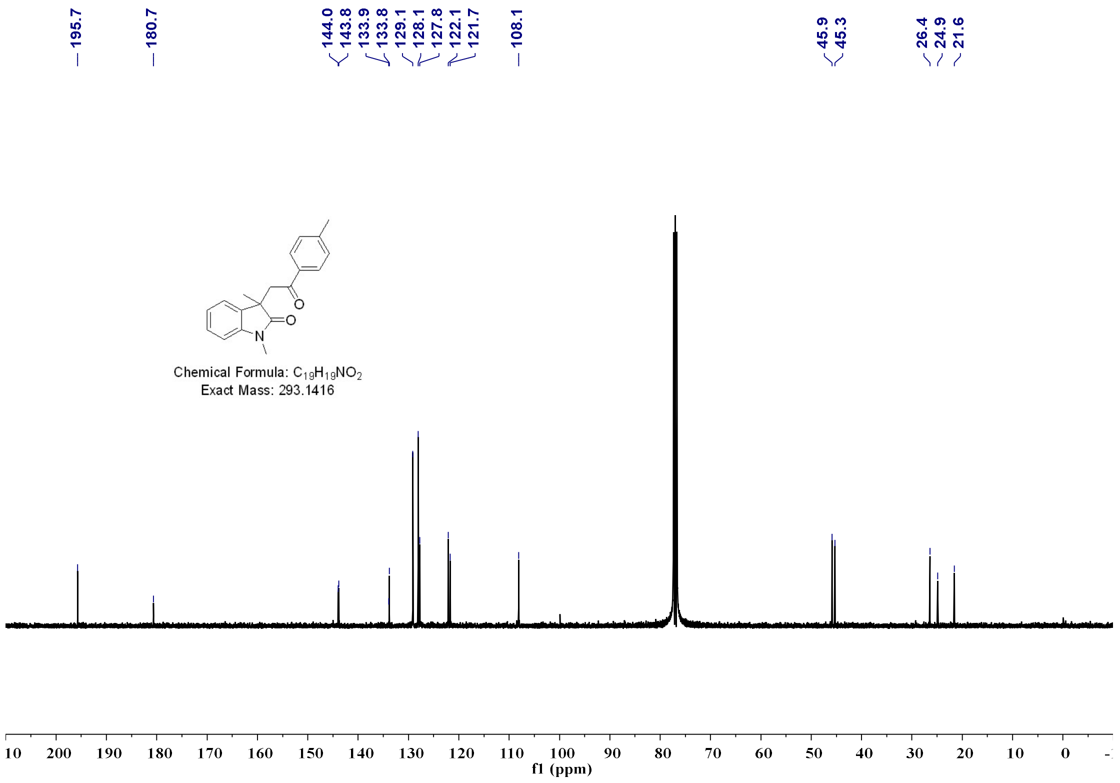
195.7
180.7
144.0
143.8
133.9
133.8
129.1
128.1
127.8
122.1
121.7
108.1
45.9
45.3
26.4
24.9
21.6
Chemical Formula: $C_{19}H_{19}NO_2$
Exact Mass: 293.1416
f1 (ppm)

3s

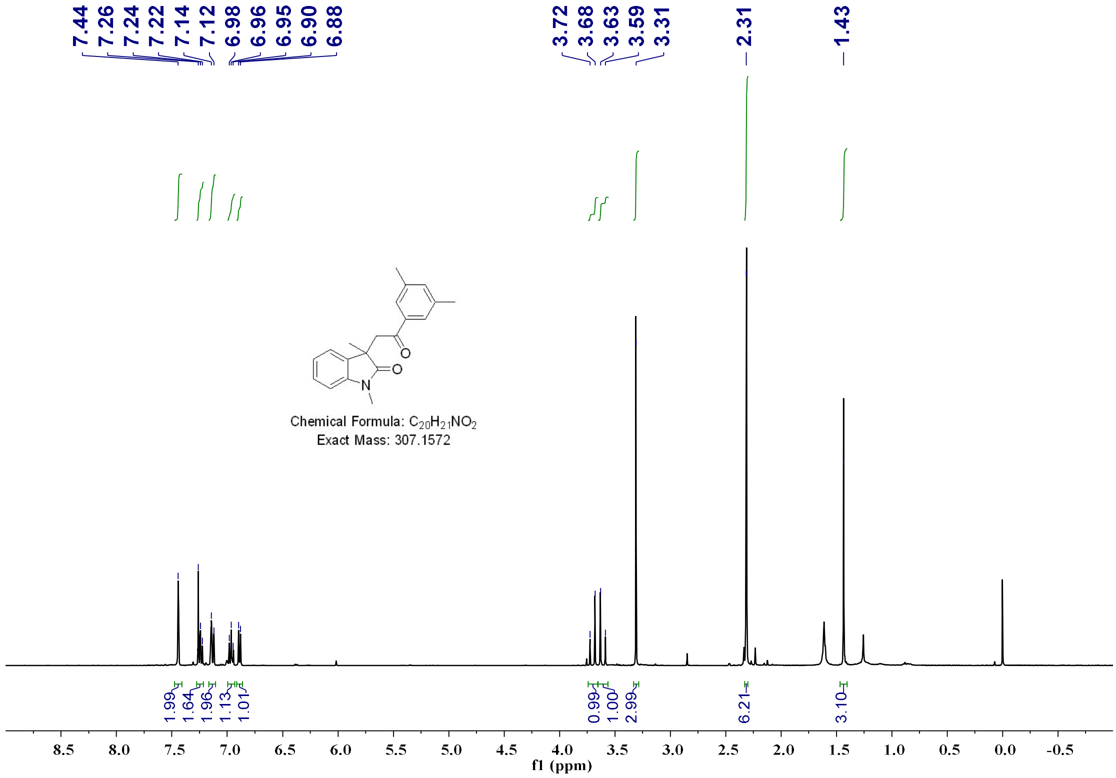

7.44
7.26
7.24
7.22
7.14
7.12
6.98
6.96
6.95
6.90
6.88
3.72
3.68
3.63
3.59
3.31
2.31
1.43
Chemical Formula: $C_{20}H_{21}NO_2$
Exact Mass: 307.1572
1.99
1.64
1.96
1.13
1.01
0.99
1.00
2.99
6.21
3.10
f1 (ppm)

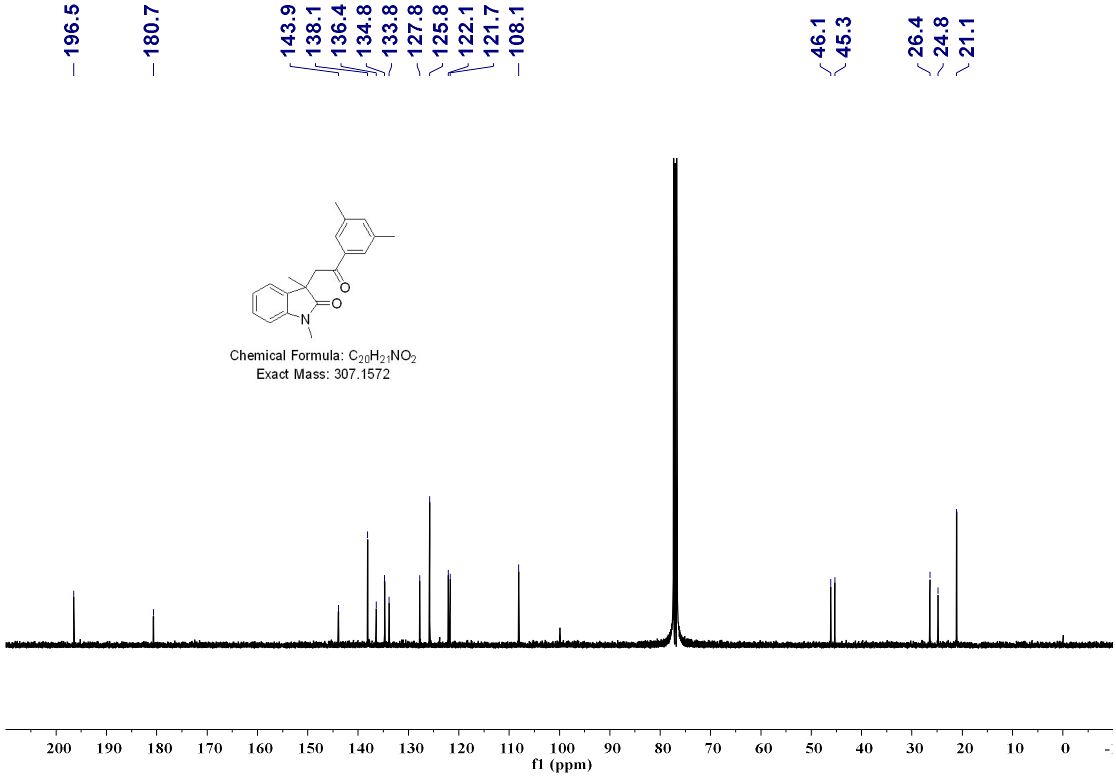

196.5
180.7
143.9
138.1
136.4
134.8
133.8
127.8
125.8
122.1
121.7
108.1
46.1
45.3
26.4
24.8
21.1
Chemical Formula: $C_{20}H_{21}NO_2$
Exact Mass: 307.1572
f1 (ppm)

3t

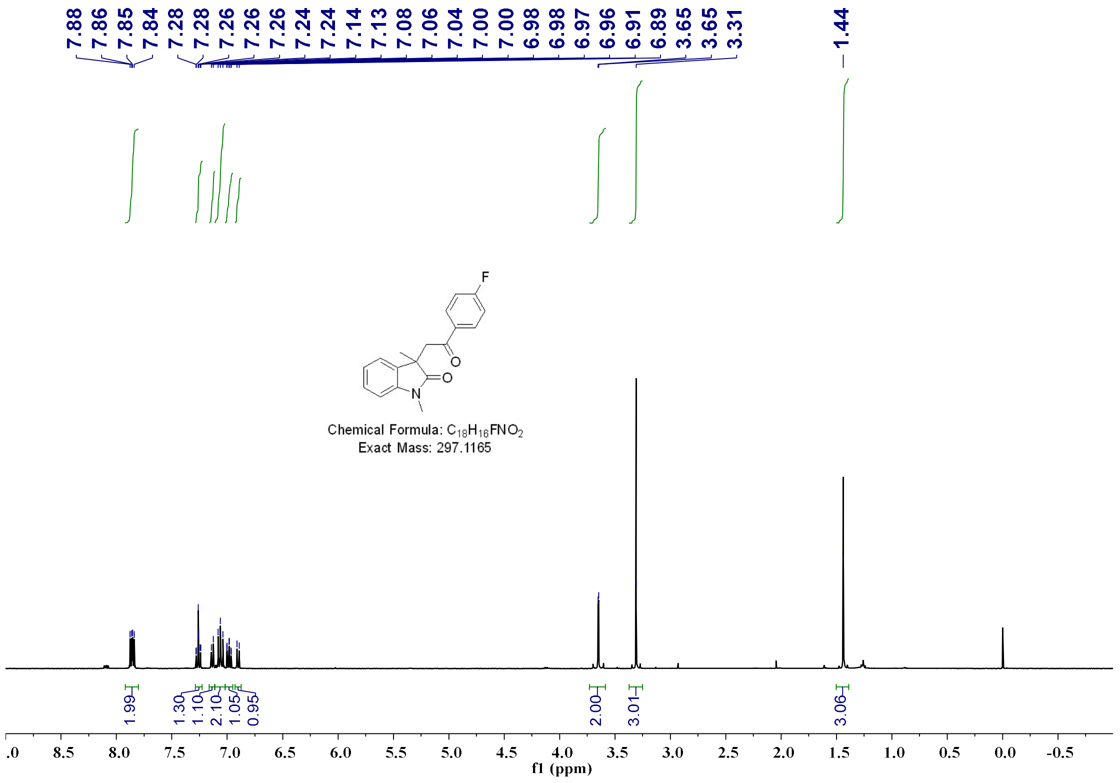

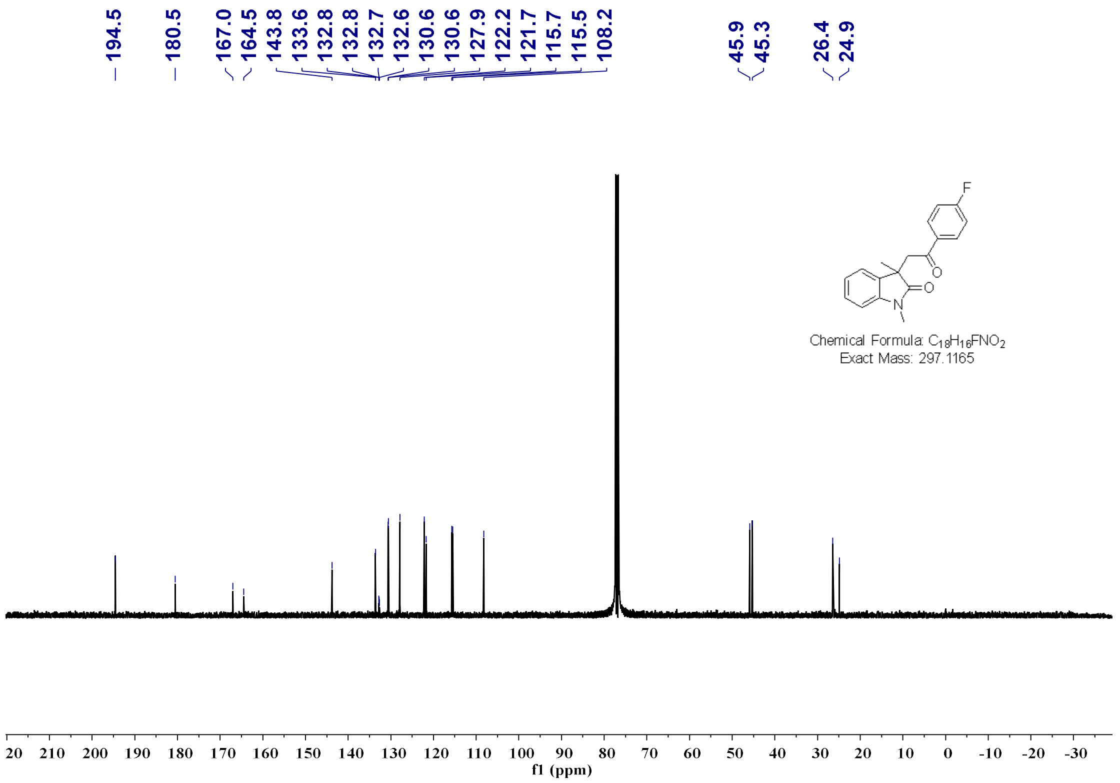

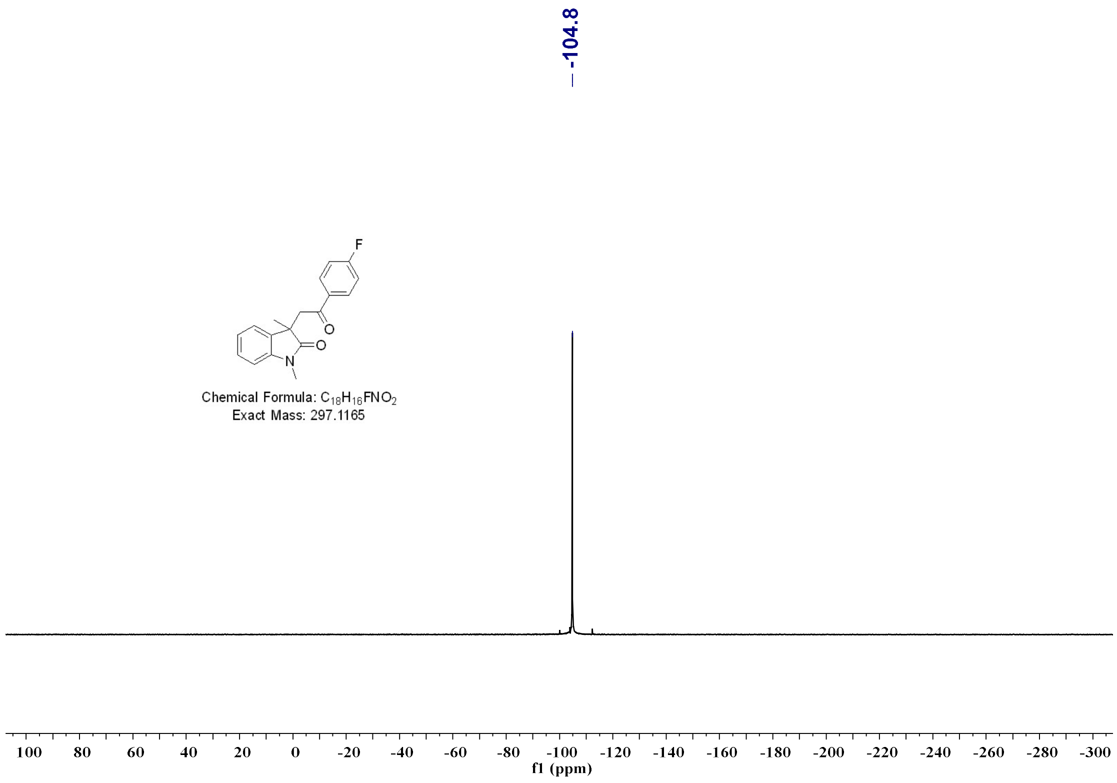

-104.8
Chemical Formula: $C_{18}H_{16}FNO_2$
Exact Mass: 297.1165
100 80 60 40 20 0 -20 -40 -60 -80 -100 -120 -140 -160 -180 -200 -220 -240 -260 -280 -300
f1 (ppm)

3u

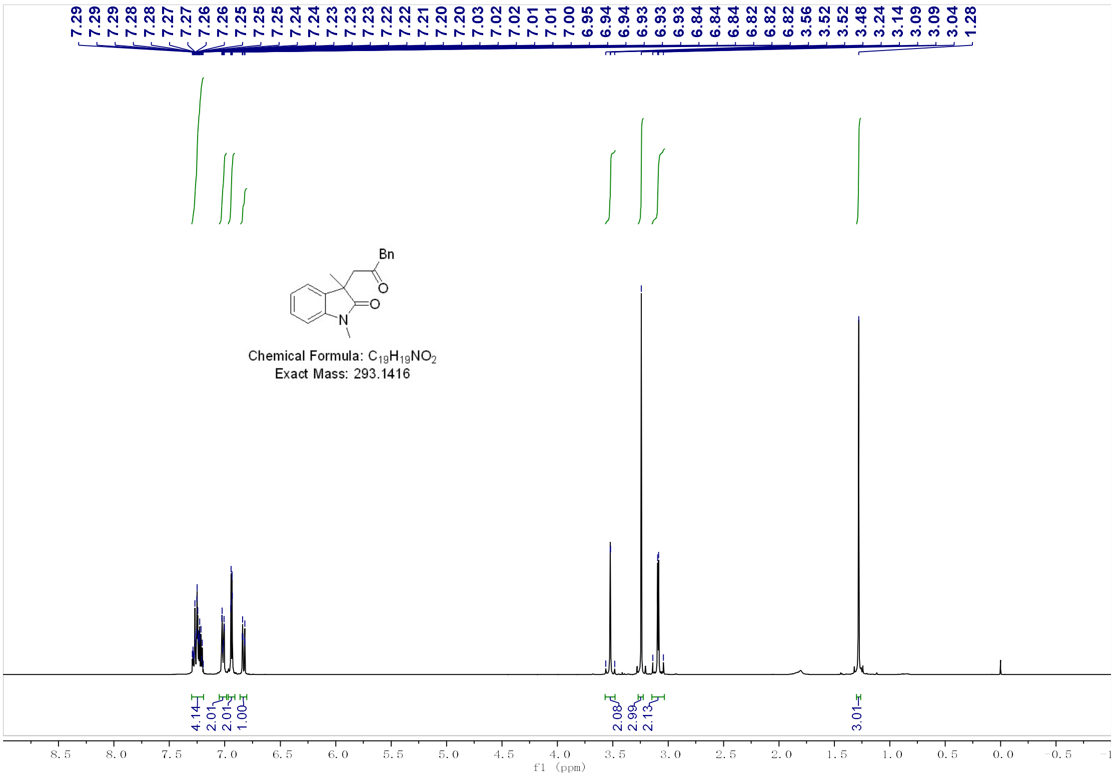
7.29
7.29
7.28
7.28
7.27
7.27
7.26
7.26
7.25
7.25
7.24
7.24
7.23
7.23
7.23
7.22
7.22
7.21
7.20
7.20
7.03
7.02
7.02
7.01
7.01
7.00
6.95
6.94
6.94
6.93
6.93
6.93
6.84
6.84
6.84
6.82
6.82
6.82
3.56
3.52
3.52
3.48
3.24
3.14
3.09
3.09
3.04
1.28
Bn
O
N
O
Chemical Formula: $C_{19}H_{19}NO_2$
Exact Mass: 293.1416
4.14
2.01
2.01
1.00
2.08
2.99
2.13
3.01
f1 (ppm)

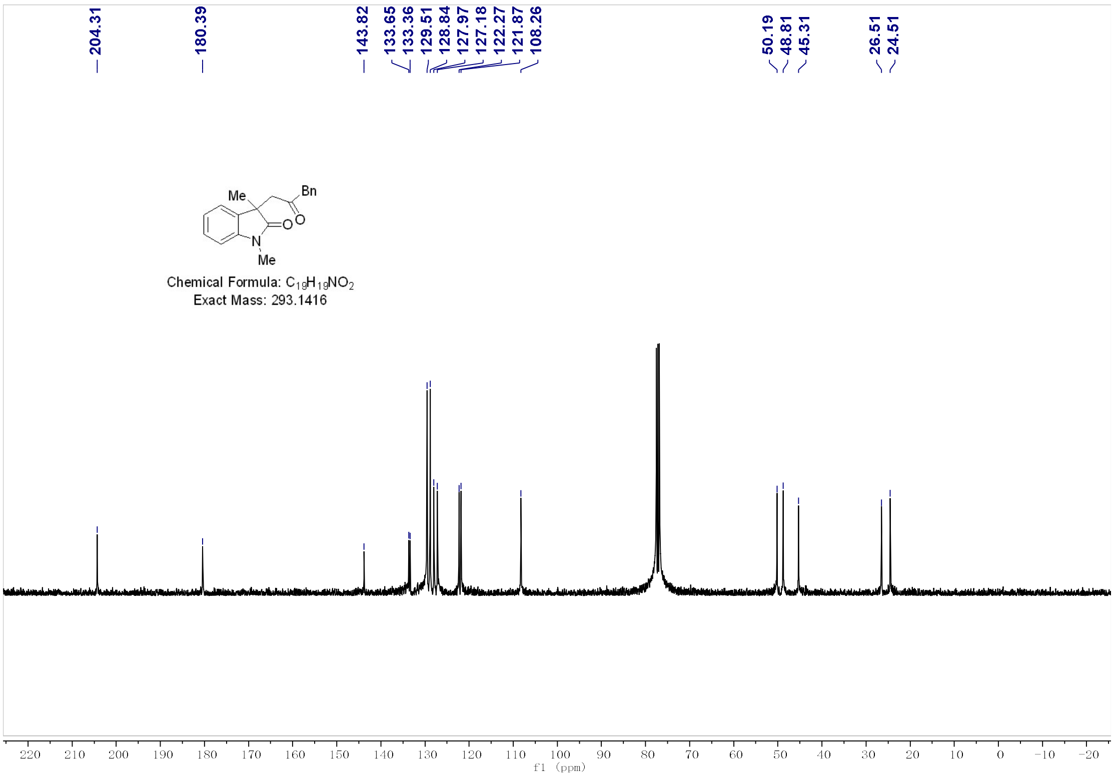
204.31
180.39
143.82
133.65
133.36
129.51
128.84
127.97
127.18
122.27
121.87
108.26
50.19
48.81
45.31
26.51
24.51
Me
Bn
O
N
O
Me
Chemical Formula: $C_{19}H_{19}NO_2$
Exact Mass: 293.1416
f1 (ppm)

6a

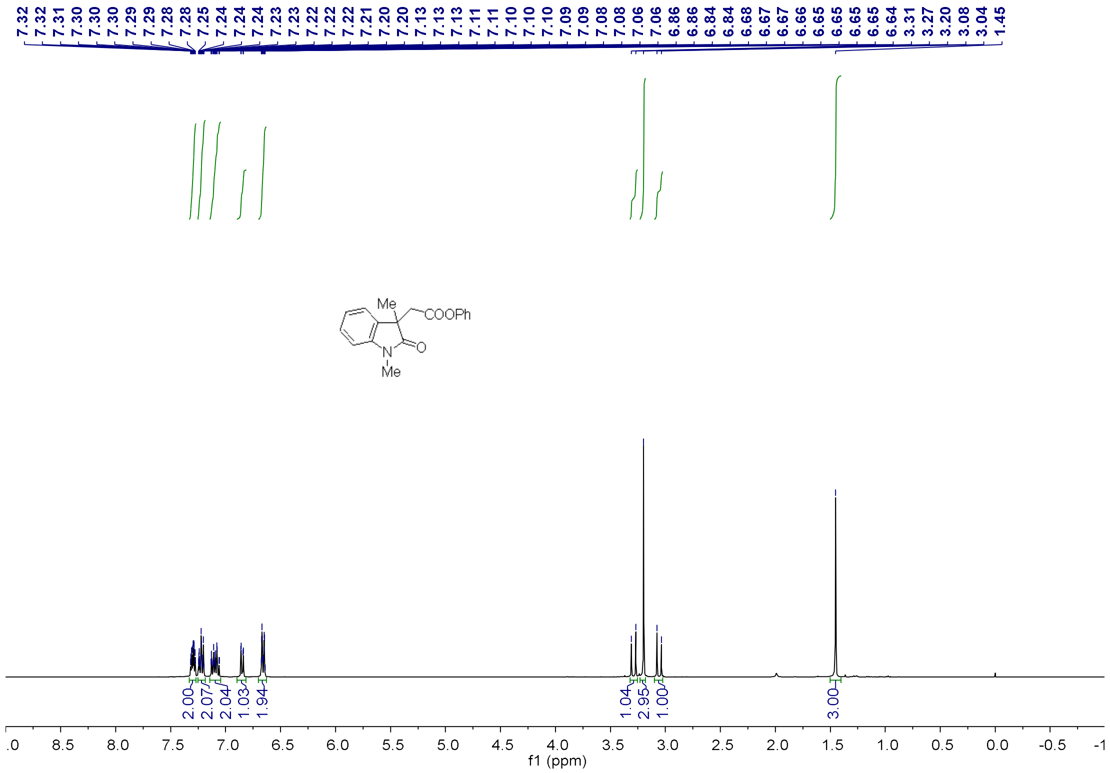
7.32
7.32
7.31
7.30
7.30
7.30
7.29
7.29
7.28
7.28
7.25
7.24
7.24
7.24
7.23
7.23
7.22
7.22
7.22
7.21
7.20
7.20
7.13
7.13
7.13
7.11
7.11
7.10
7.10
7.10
7.09
7.09
7.08
7.08
7.06
7.06
6.86
6.86
6.84
6.84
6.68
6.67
6.67
6.66
6.66
6.65
6.65
6.65
6.65
6.64
3.31
3.27
3.20
3.08
3.04
1.45
Me
COOPh
N
O
Me
2.00
2.07
2.04
1.03
1.94
1.04
2.95
1.00
3.00
f1 (ppm)

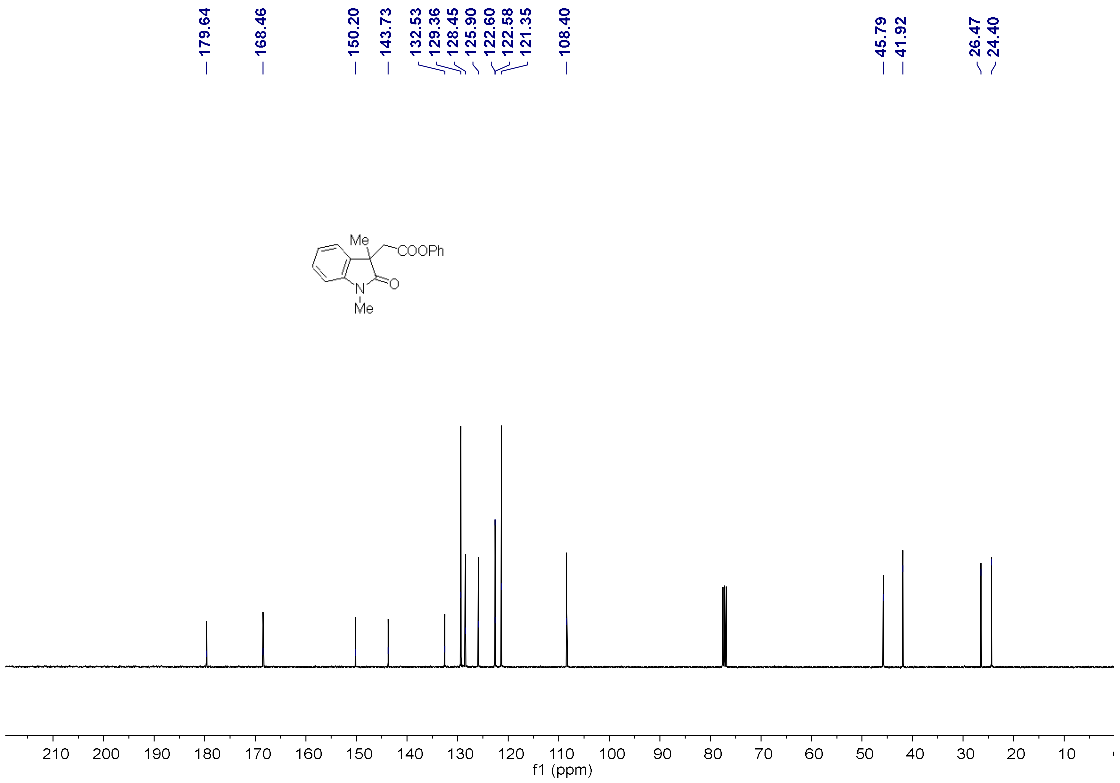
179.64
168.46
150.20
143.73
132.53
129.36
128.45
125.90
122.60
122.58
121.35
108.40
45.79
41.92
26.47
24.40
Me
COOPh
N
O
Me
f1 (ppm)

6b

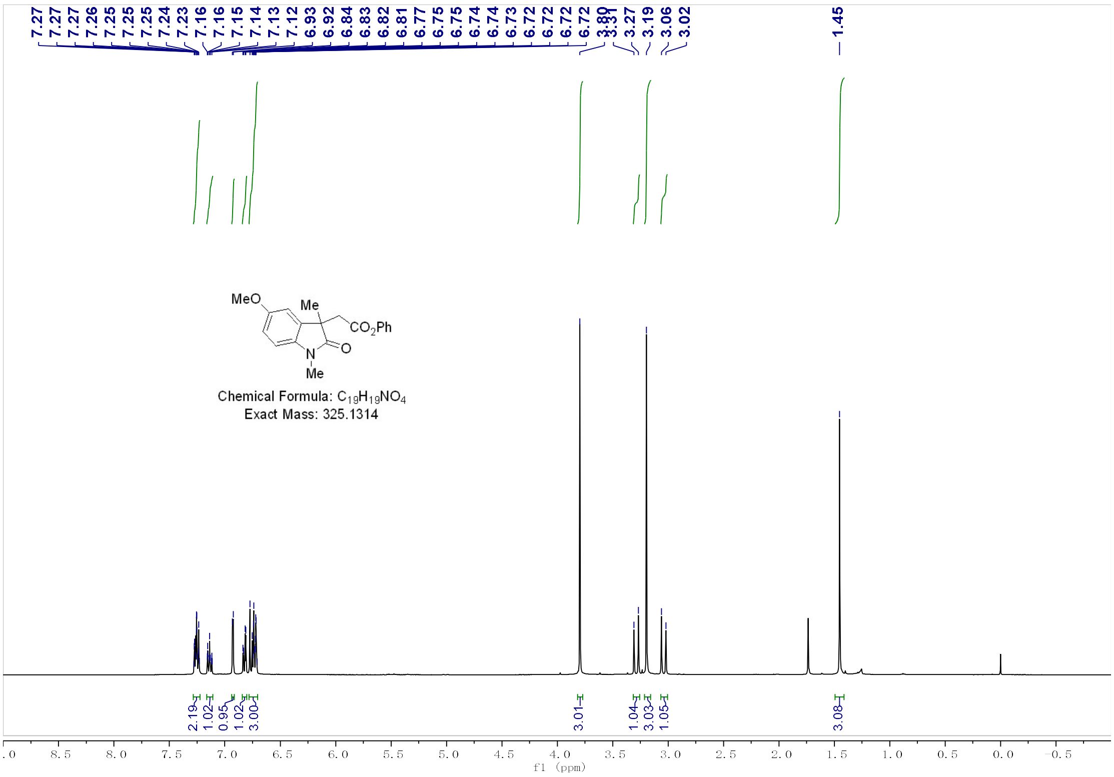

7.27
7.27
7.27
7.26
7.25
7.25
7.25
7.24
7.23
7.16
7.16
7.15
7.14
7.13
7.12
6.93
6.92
6.84
6.83
6.82
6.81
6.77
6.75
6.75
6.74
6.74
6.73
6.72
6.72
6.72
6.72
3.80
3.31
3.27
3.19
3.06
3.02
1.45
MeO
Me
CO_2Ph
N
O
Me
Chemical Formula: $C_{19}H_{19}NO_4$
Exact Mass: 325.1314
2.19
1.02
0.95
1.02
3.00
3.01
1.04
3.03
1.05
3.08
f1 (ppm)

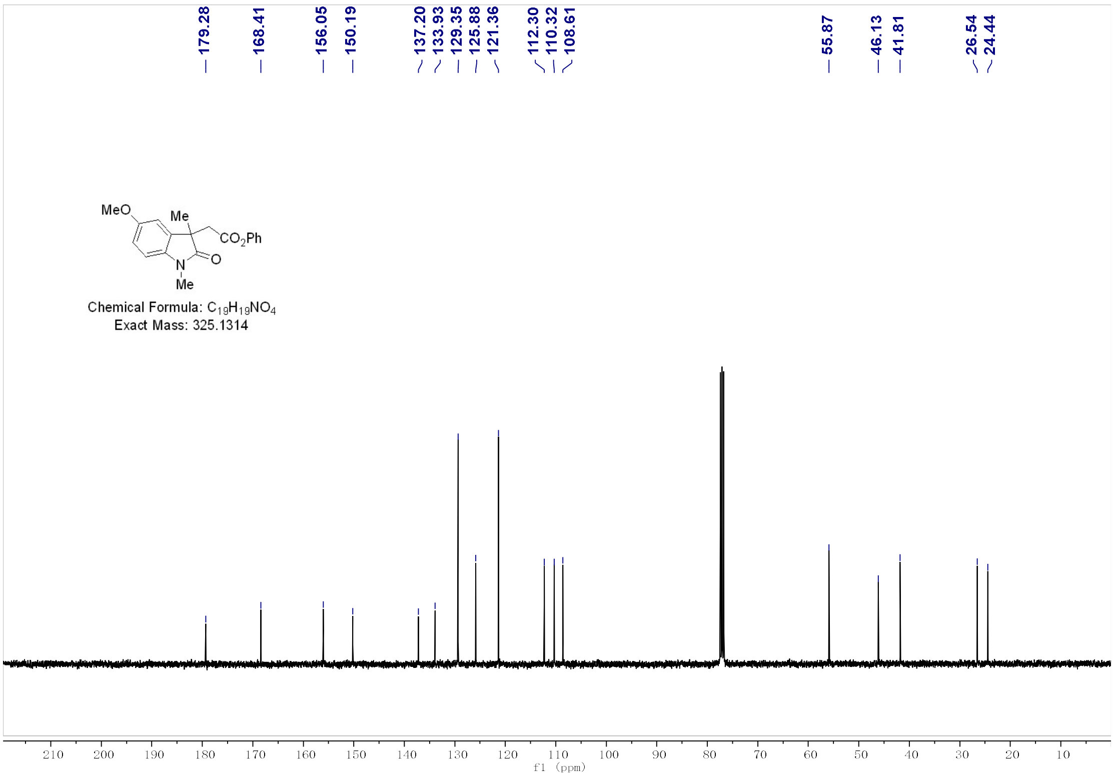

179.28
168.41
156.05
150.19
137.20
133.93
129.35
125.88
121.36
112.30
110.32
108.61
55.87
46.13
41.81
26.54
24.44
MeO
Me
CO_2Ph
N
O
Me
Chemical Formula: $C_{19}H_{19}NO_4$
Exact Mass: 325.1314
f1 (ppm)

6c

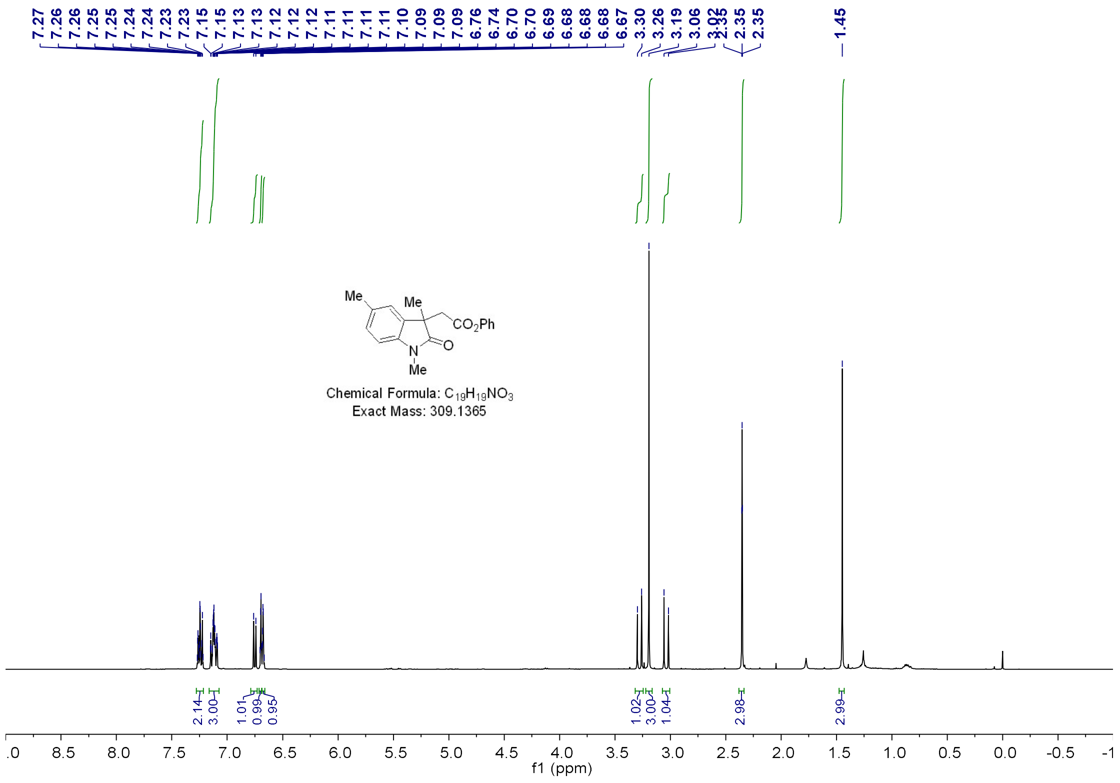

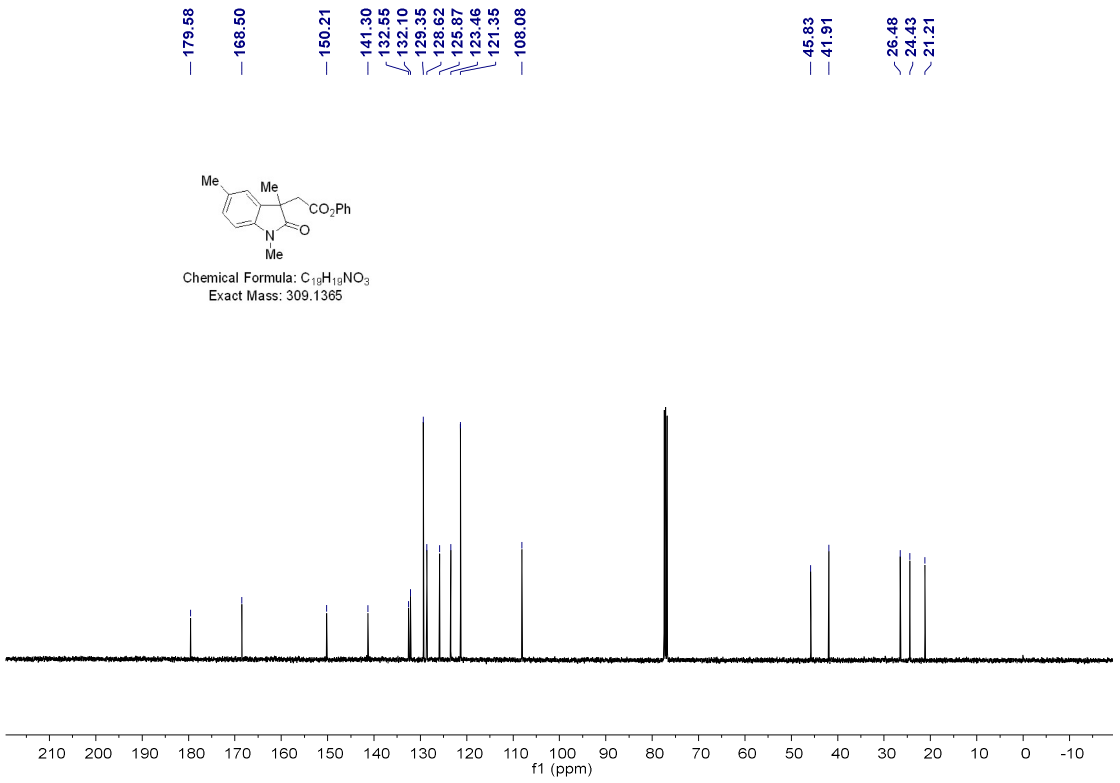

6d

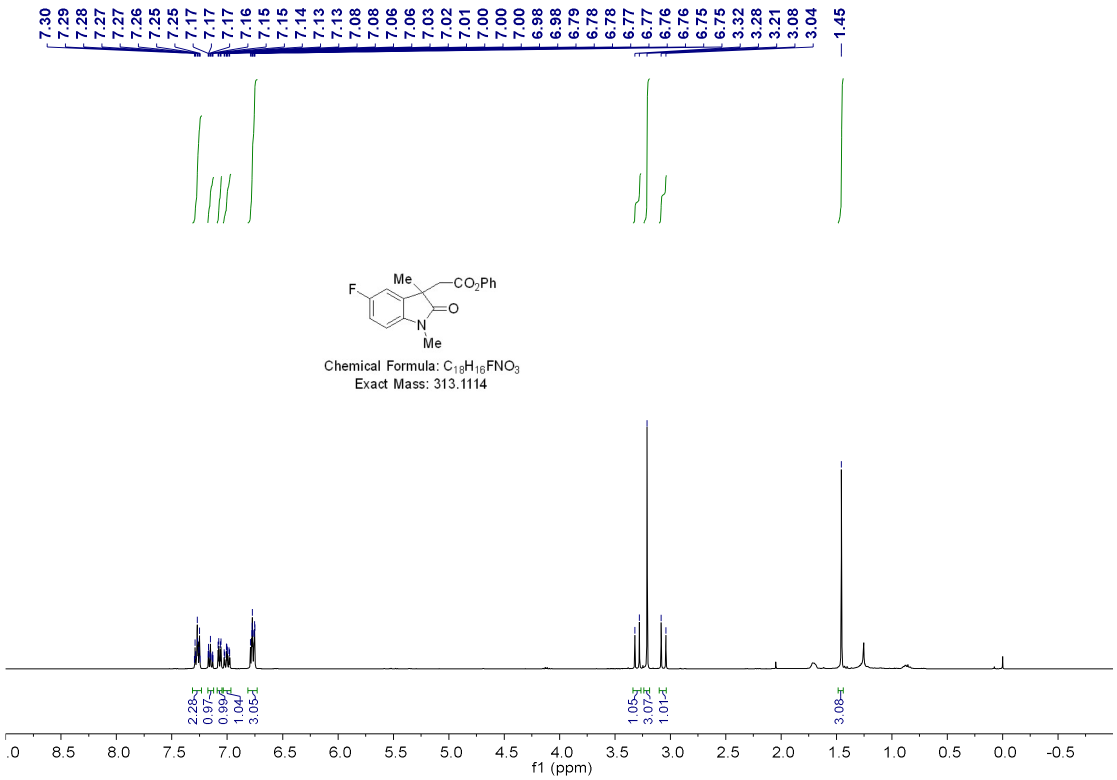

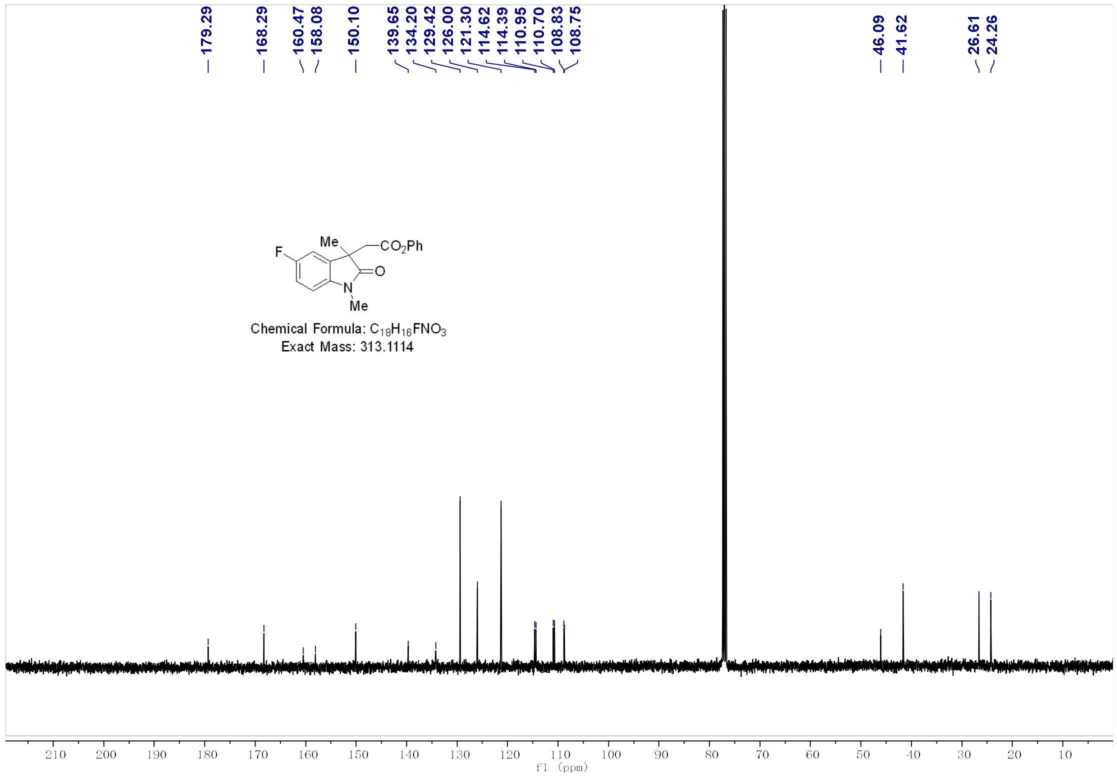

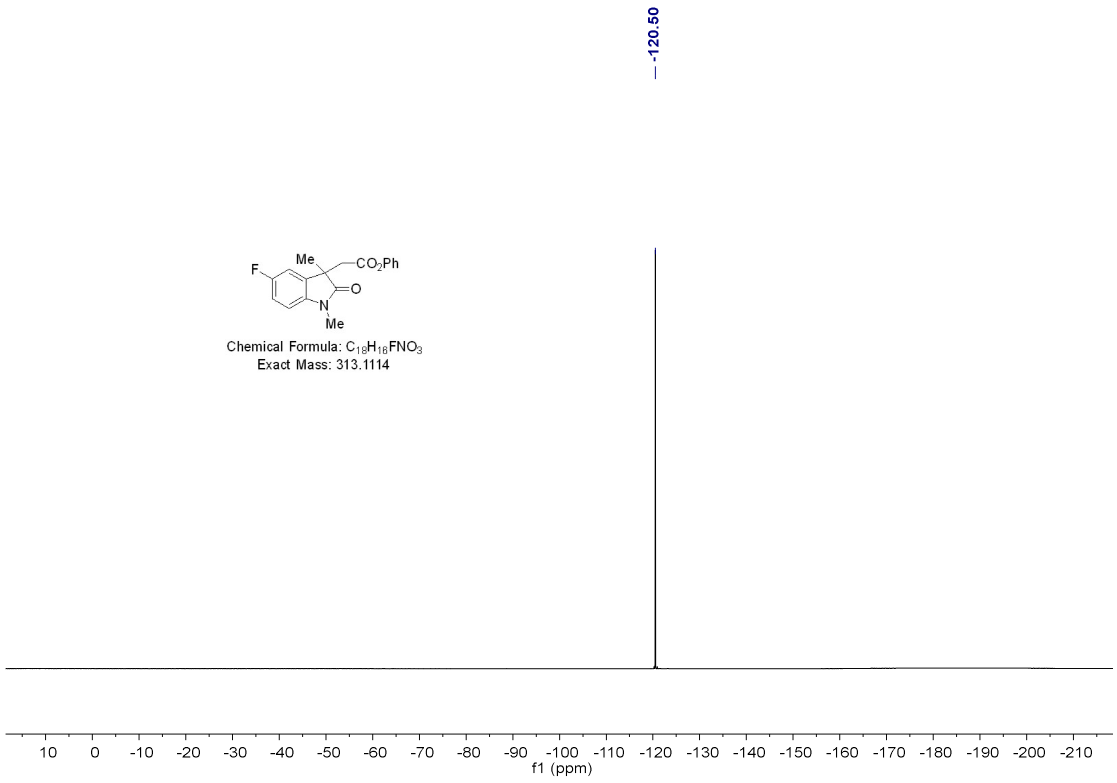
-120.50
F
Me
CO2Ph
O
N
Me
Chemical Formula: C18H16FNO3
Exact Mass: 313.1114
10 0 -10 -20 -30 -40 -50 -60 -70 -80 -90 -100 -110 -120 -130 -140 -150 -160 -170 -180 -190 -200 -210
f1 (ppm)

6e

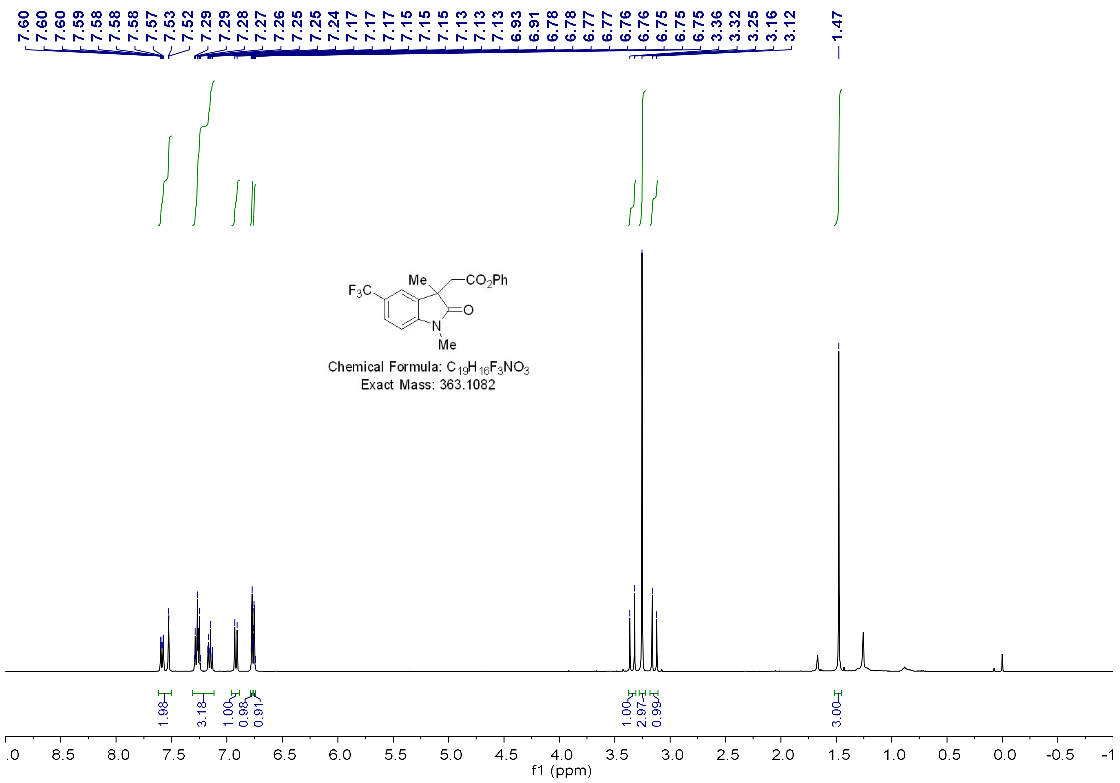

7.60
7.60
7.59
7.58
7.58
7.58
7.57
7.53
7.52
7.29
7.29
7.28
7.27
7.26
7.25
7.25
7.24
7.17
7.17
7.17
7.15
7.15
7.15
7.13
7.13
7.13
6.93
6.91
6.78
6.78
6.77
6.76
6.76
6.75
6.75
6.75
3.36
3.32
3.25
3.16
3.12
1.47
F3C
Me
CO2Ph
O
N
Me
Chemical Formula: C19H16F3NO3
Exact Mass: 363.1082
1.98
3.18
1.00
0.98
0.91
1.00
2.97
0.99
3.00
f1 (ppm)

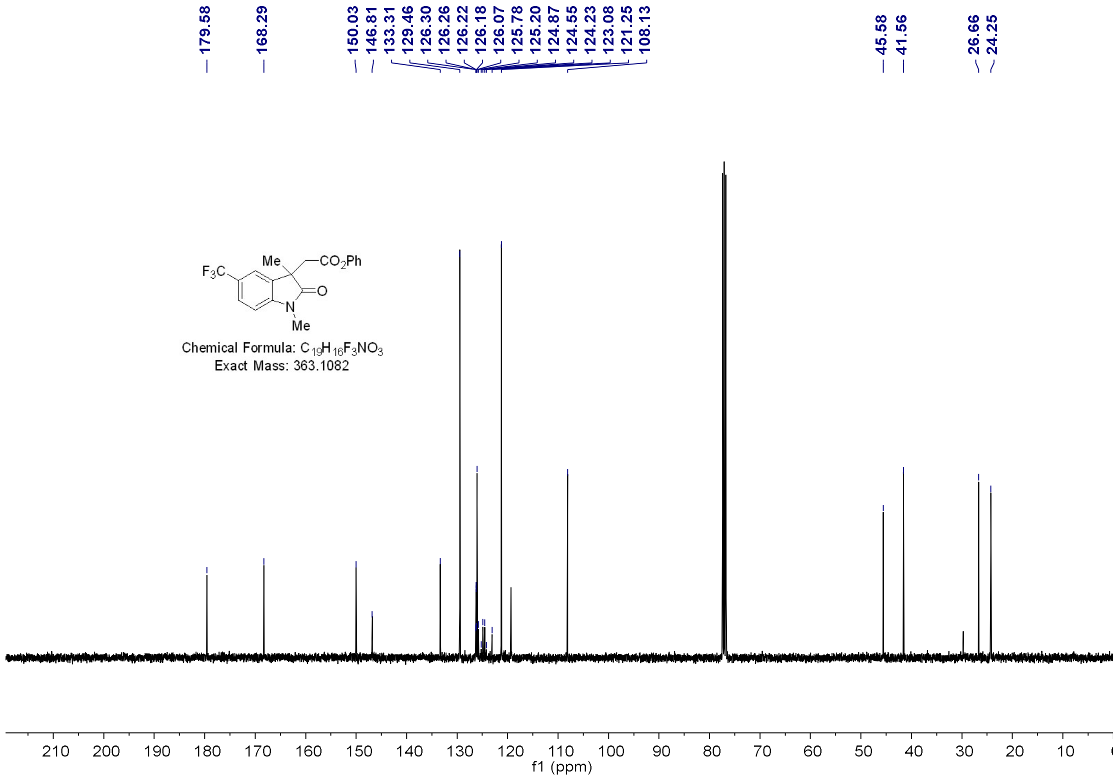

179.58
168.29
150.03
146.81
133.31
129.46
126.30
126.26
126.22
126.18
126.07
125.78
125.20
124.87
124.55
124.23
123.08
121.25
108.13
45.58
41.56
26.66
24.25
F3C
Me
CO2Ph
O
N
Me
Chemical Formula: C19H16F3NO3
Exact Mass: 363.1082
f1 (ppm)

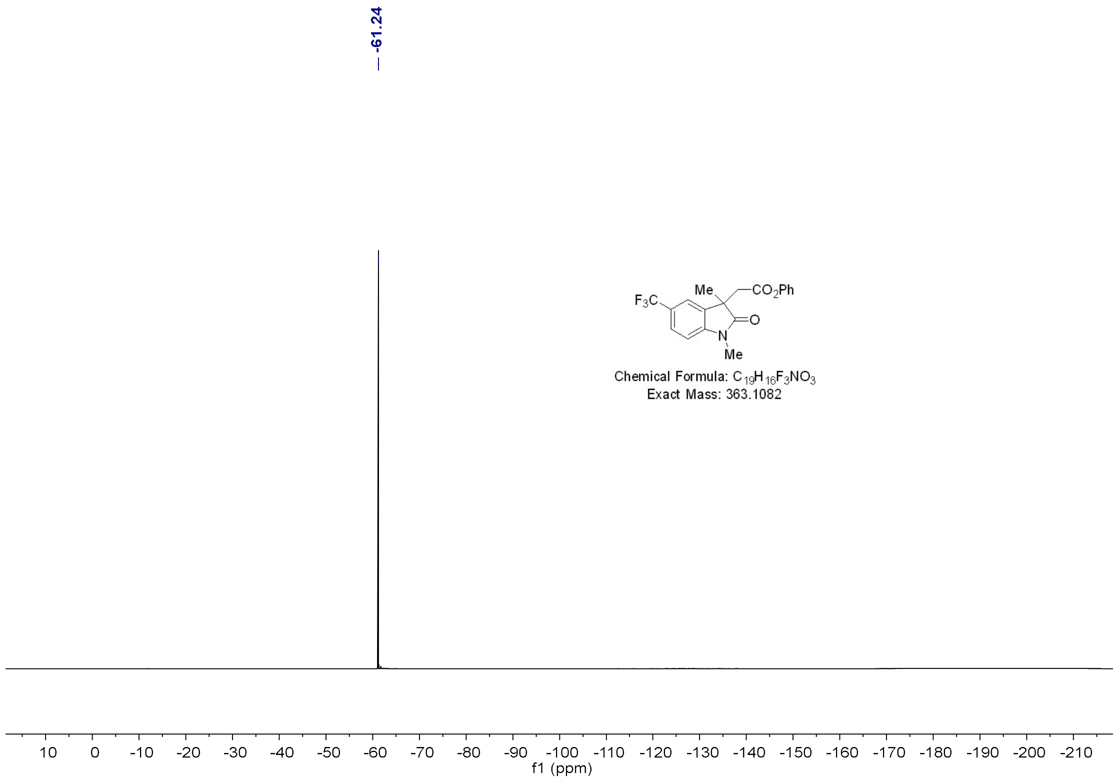
-61.24
Me
CO_2Ph
F_3C
O
N
Me
Chemical Formula: $C_{19}H_{16}F_3NO_3$
Exact Mass: 363.1082
10 0 -10 -20 -30 -40 -50 -60 -70 -80 -90 -100 -110 -120 -130 -140 -150 -160 -170 -180 -190 -200 -210
f1 (ppm)

6f

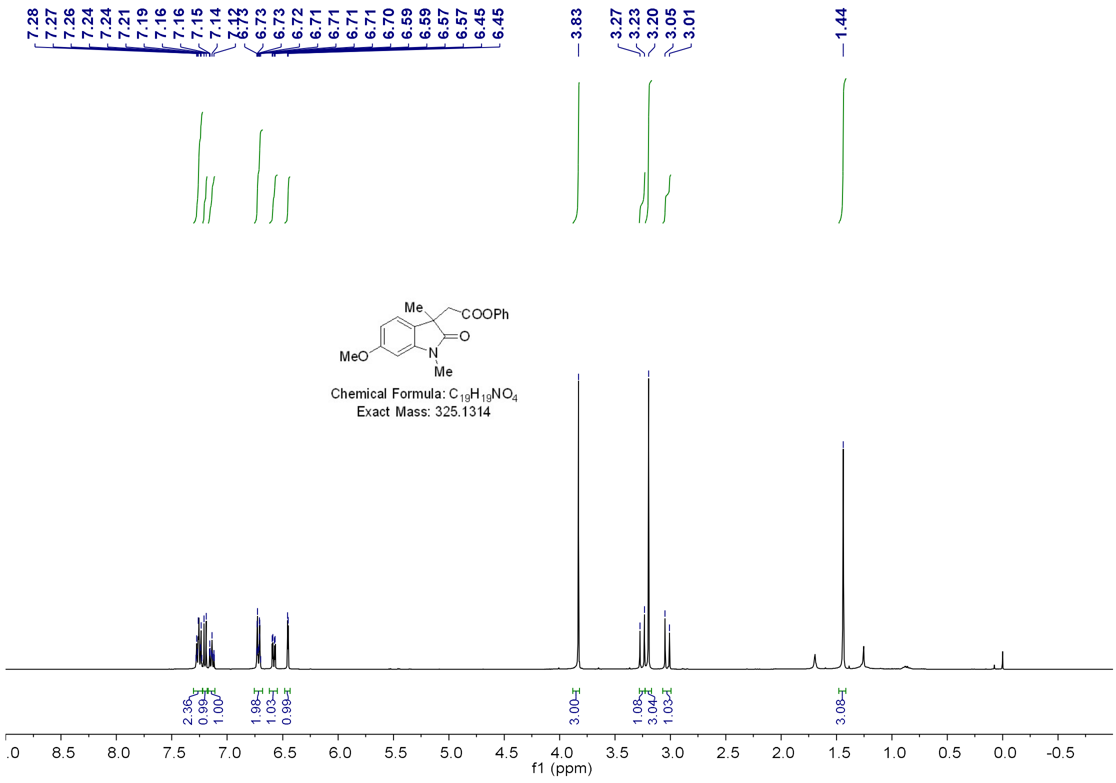
7.28
7.27
7.26
7.24
7.24
7.21
7.19
7.16
7.16
7.15
7.14
7.12
6.73
6.73
6.72
6.71
6.71
6.71
6.70
6.59
6.59
6.57
6.57
6.45
6.45
3.83
3.27
3.23
3.20
3.05
3.01
1.44
Me
COOPh
O
MeO
N
Me
Chemical Formula: $C_{19}H_{19}NO_4$
Exact Mass: 325.1314
2.36
0.99
1.00
1.98
1.03
0.99
3.00
1.08
3.04
1.03
3.08
f1 (ppm)

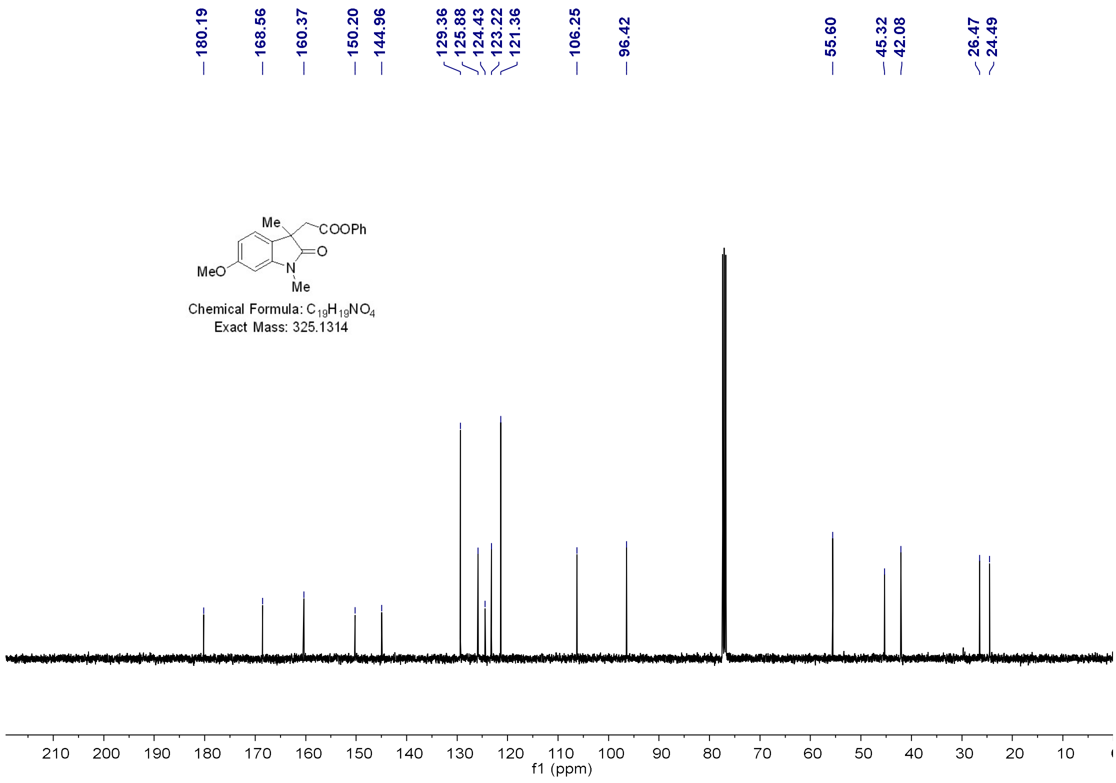
180.19
168.56
160.37
150.20
144.96
129.36
125.88
124.43
123.22
121.36
106.25
96.42
55.60
45.32
42.08
26.47
24.49
Me
COOPh
O
MeO
N
Me
Chemical Formula: $C_{19}H_{19}NO_4$
Exact Mass: 325.1314
f1 (ppm)

6g

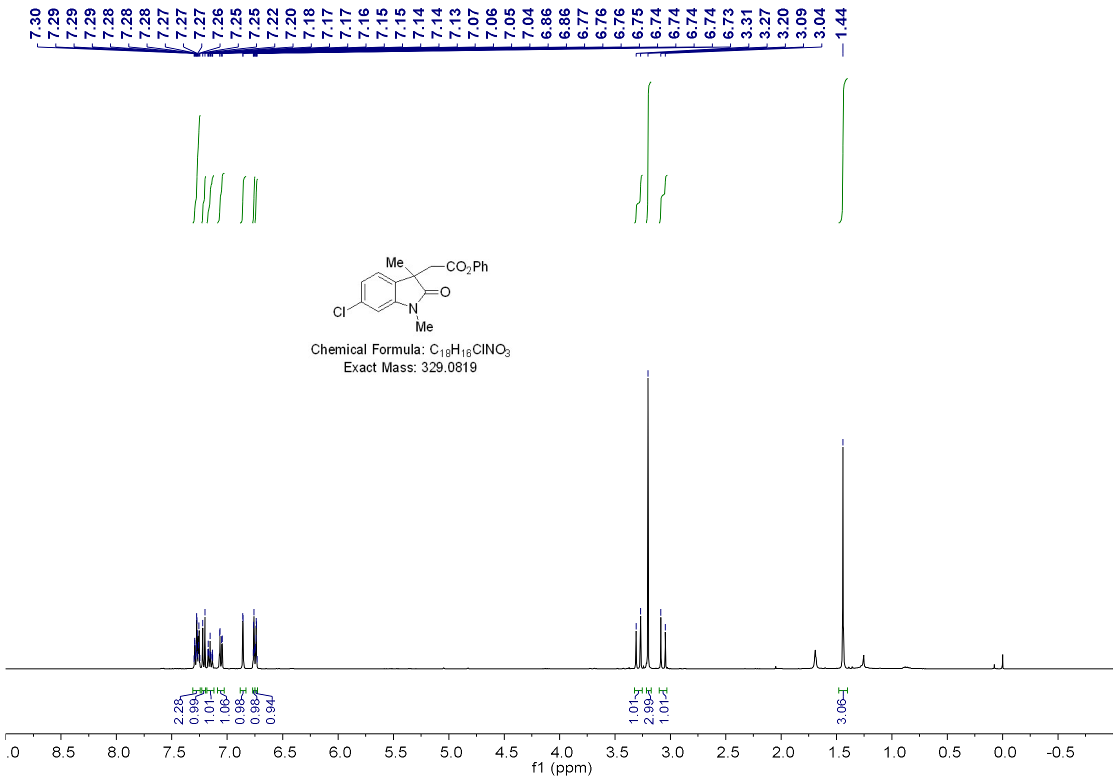

7.30
7.29
7.29
7.28
7.28
7.28
7.27
7.27
7.26
7.25
7.25
7.22
7.20
7.18
7.17
7.17
7.16
7.15
7.15
7.14
7.14
7.13
7.07
7.06
7.05
7.04
6.86
6.86
6.77
6.76
6.76
6.75
6.74
6.74
6.74
6.74
6.73
3.31
3.27
3.20
3.09
3.04
1.44
Me
CO2Ph
O
Cl
N
Me
Chemical Formula: C18H16ClNO3
Exact Mass: 329.0819
2.28
0.99
1.01
1.06
0.98
0.98
0.94
1.01
2.99
1.01
3.06
f1 (ppm)

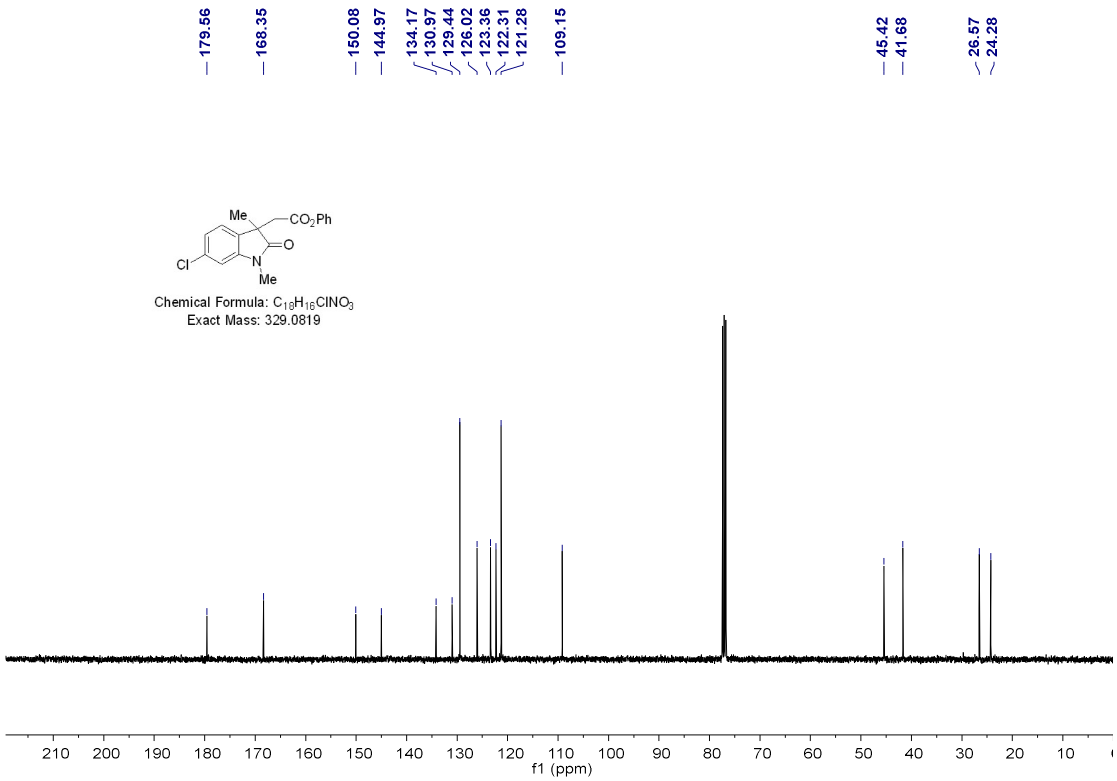

179.56
168.35
150.08
144.97
134.17
130.97
129.44
126.02
123.36
122.31
121.28
109.15
45.42
41.68
26.57
24.28
Me
CO2Ph
O
Cl
N
Me
Chemical Formula: C18H16ClNO3
Exact Mass: 329.0819
f1 (ppm)

6h

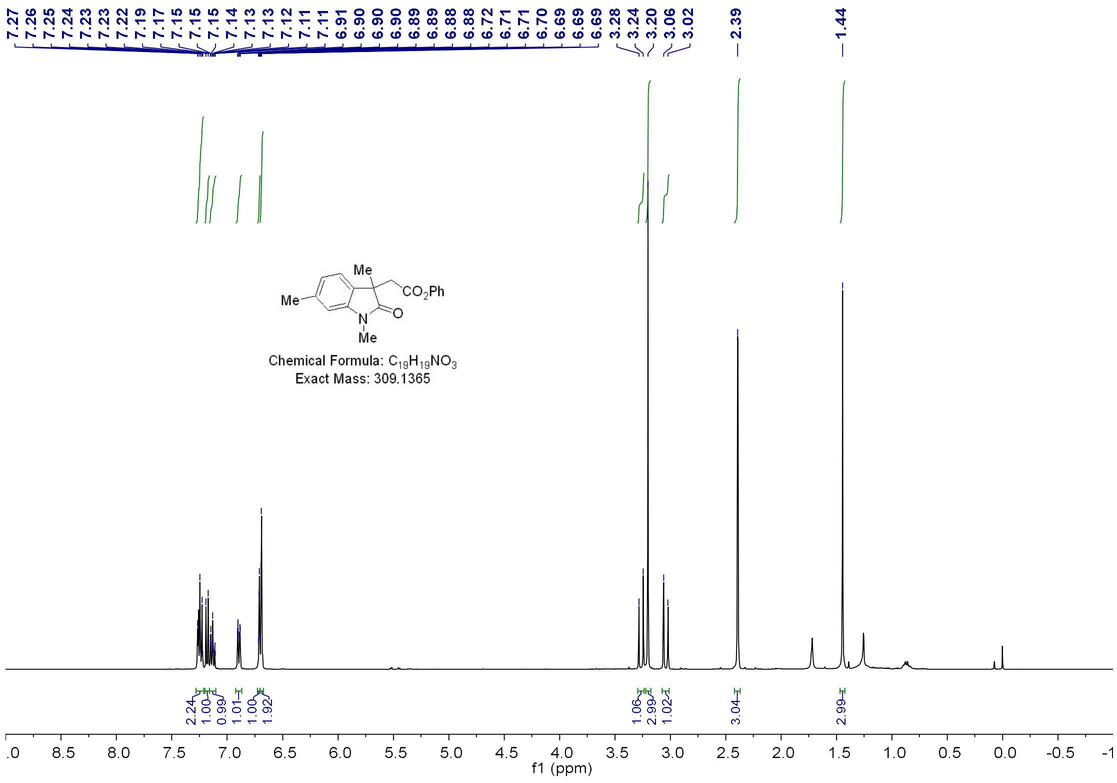

7.27
7.26
7.25
7.24
7.23
7.23
7.22
7.19
7.17
7.15
7.15
7.14
7.13
7.13
7.12
7.11
7.11
6.91
6.90
6.90
6.89
6.89
6.88
6.88
6.72
6.71
6.71
6.70
6.69
6.69
6.69
3.28
3.24
3.20
3.06
3.02
2.39
1.44
Me
CO2Ph
Me
O
N
Me
Chemical Formula: C19H19NO3
Exact Mass: 309.1365
2.24
1.00
0.99
1.01
1.00
1.92
1.06
2.99
1.02
3.04
2.99
f1 (ppm)

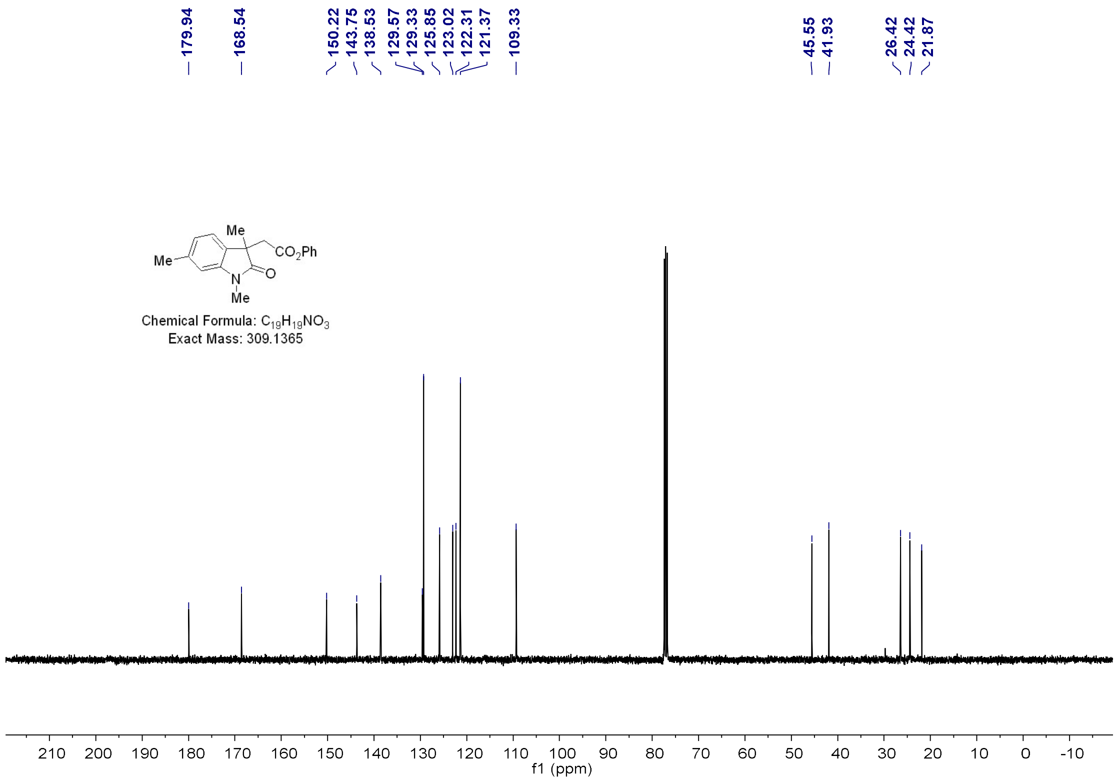

179.94
168.54
150.22
143.75
138.53
129.57
129.33
125.85
123.02
122.31
121.37
109.33
45.55
41.93
26.42
24.42
21.87
Me
CO2Ph
Me
O
N
Me
Chemical Formula: C19H19NO3
Exact Mass: 309.1365
f1 (ppm)

6i

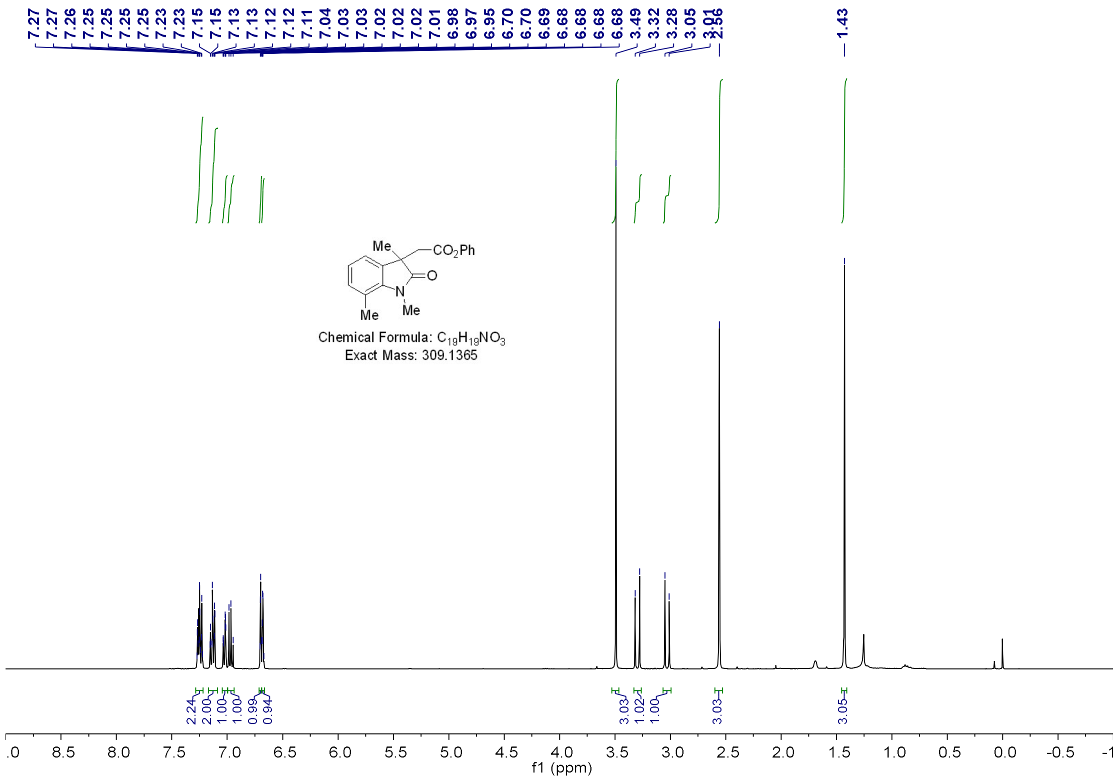
7.27
7.27
7.26
7.25
7.25
7.25
7.23
7.23
7.15
7.15
7.13
7.13
7.12
7.12
7.11
7.04
7.03
7.03
7.02
7.02
7.02
7.01
6.98
6.97
6.95
6.70
6.69
6.68
6.68
6.68
6.68
3.49
3.32
3.28
3.05
3.01
2.56
1.43
Me
CO2Ph
N
O
Me
Me
Chemical Formula: C19H19NO3
Exact Mass: 309.1365
2.24
2.00
1.00
1.00
0.99
0.94
3.03
1.02
1.00
3.03
3.05
f1 (ppm)

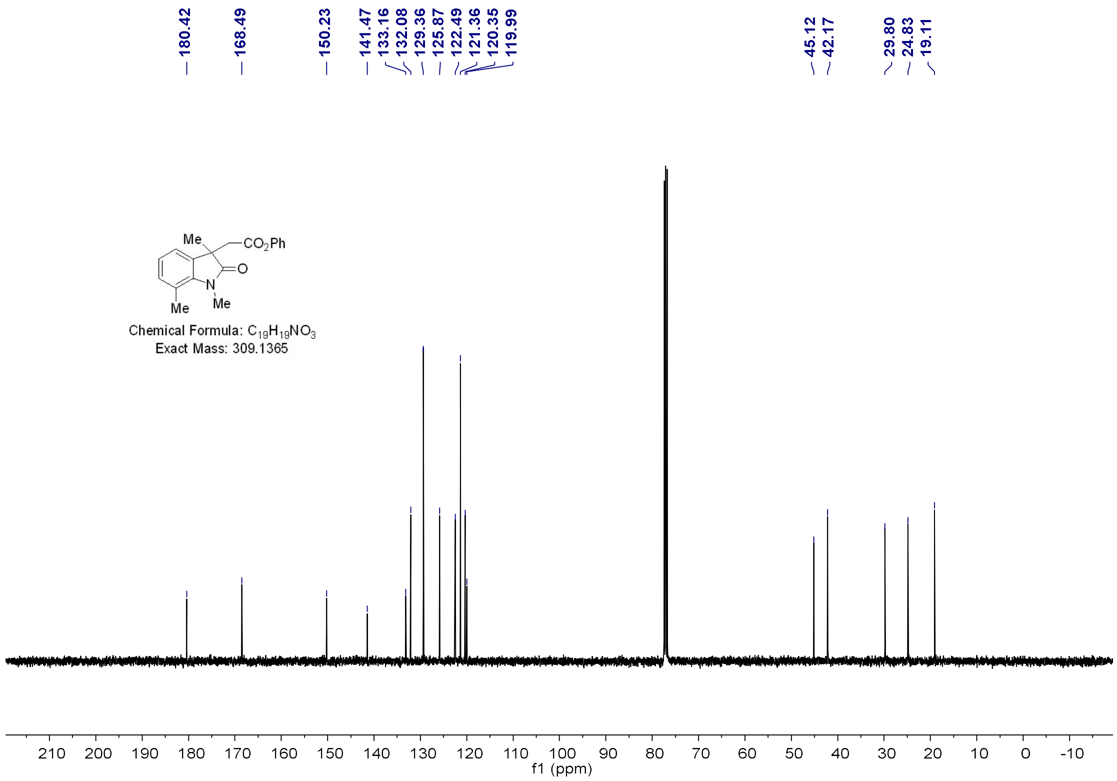
180.42
168.49
150.23
141.47
133.16
132.08
129.36
125.87
122.49
121.36
120.35
119.99
45.12
42.17
29.80
24.83
19.11
Me
CO2Ph
N
O
Me
Me
Chemical Formula: C19H19NO3
Exact Mass: 309.1365
f1 (ppm)

6j

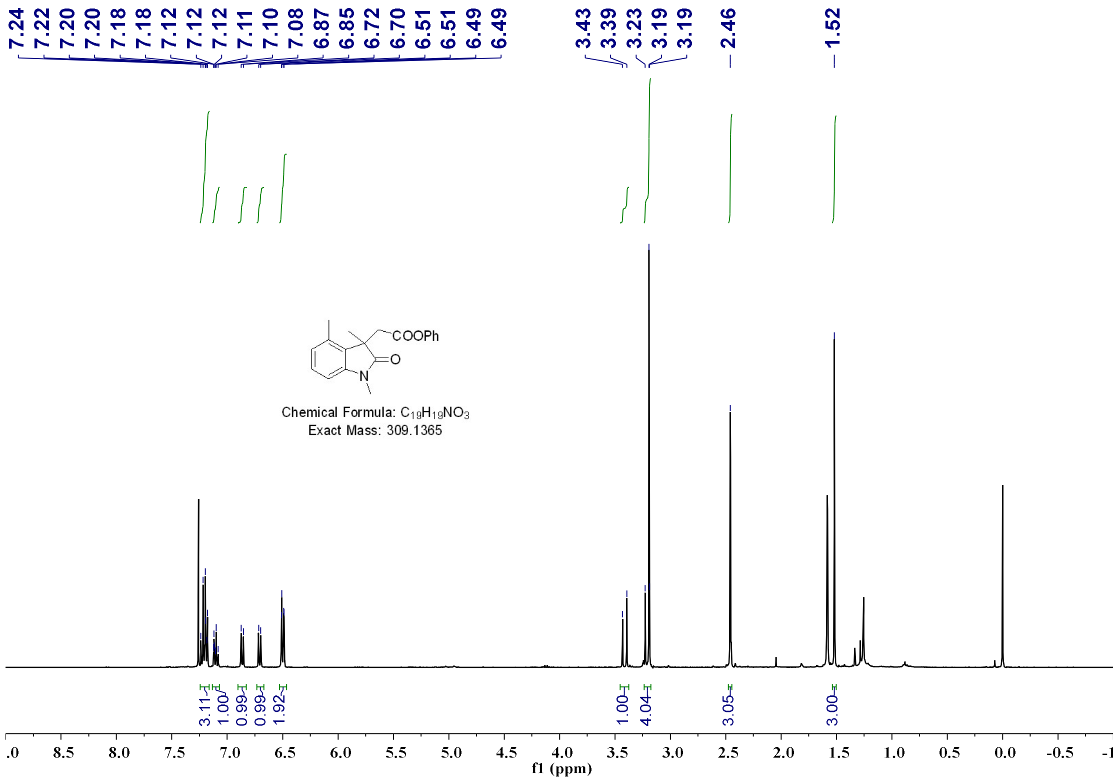
7.24
7.22
7.20
7.20
7.18
7.18
7.12
7.12
7.12
7.11
7.10
7.08
6.87
6.85
6.72
6.70
6.51
6.51
6.49
6.49
3.43
3.39
3.23
3.19
3.19
2.46
1.52
COOPh
O
N
Chemical Formula: $C_{19}H_{19}NO_3$
Exact Mass: 309.1365
3.11
1.00
0.99
0.99
1.92
1.00
4.04
3.05
3.00
f1 (ppm)

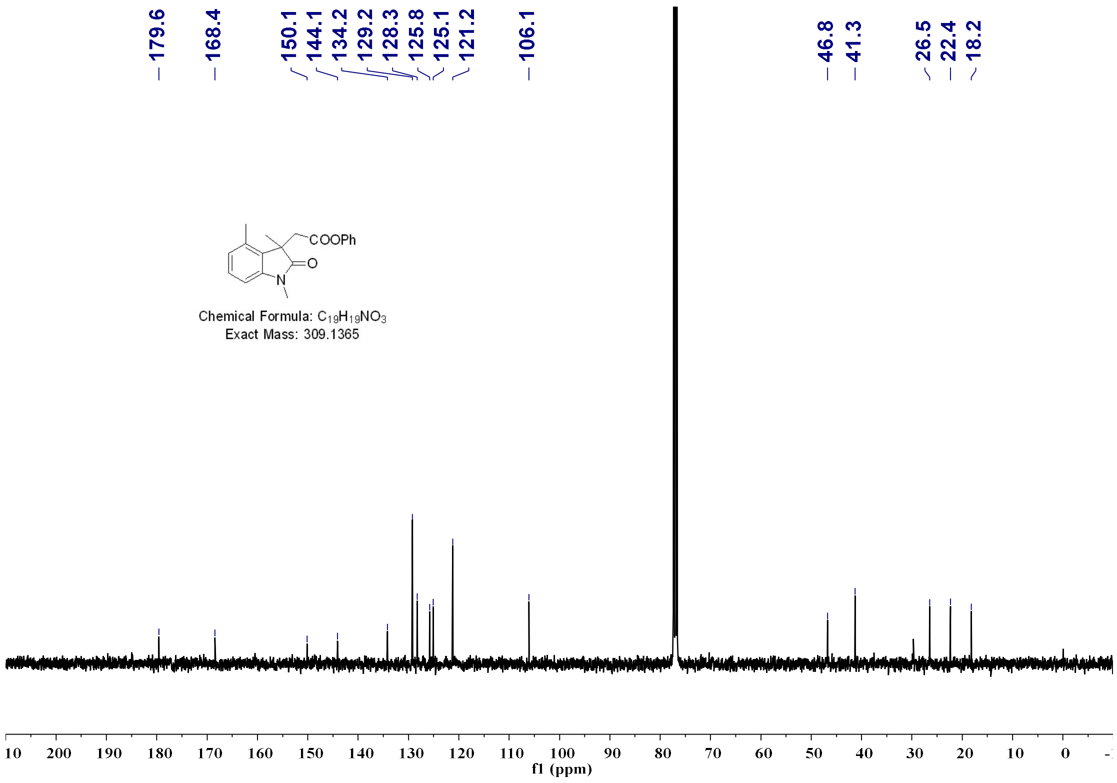
179.6
168.4
150.1
144.1
134.2
129.2
128.3
125.8
125.1
121.2
106.1
46.8
41.3
26.5
22.4
18.2
COOPh
O
N
Chemical Formula: $C_{19}H_{19}NO_3$
Exact Mass: 309.1365
f1 (ppm)

6k

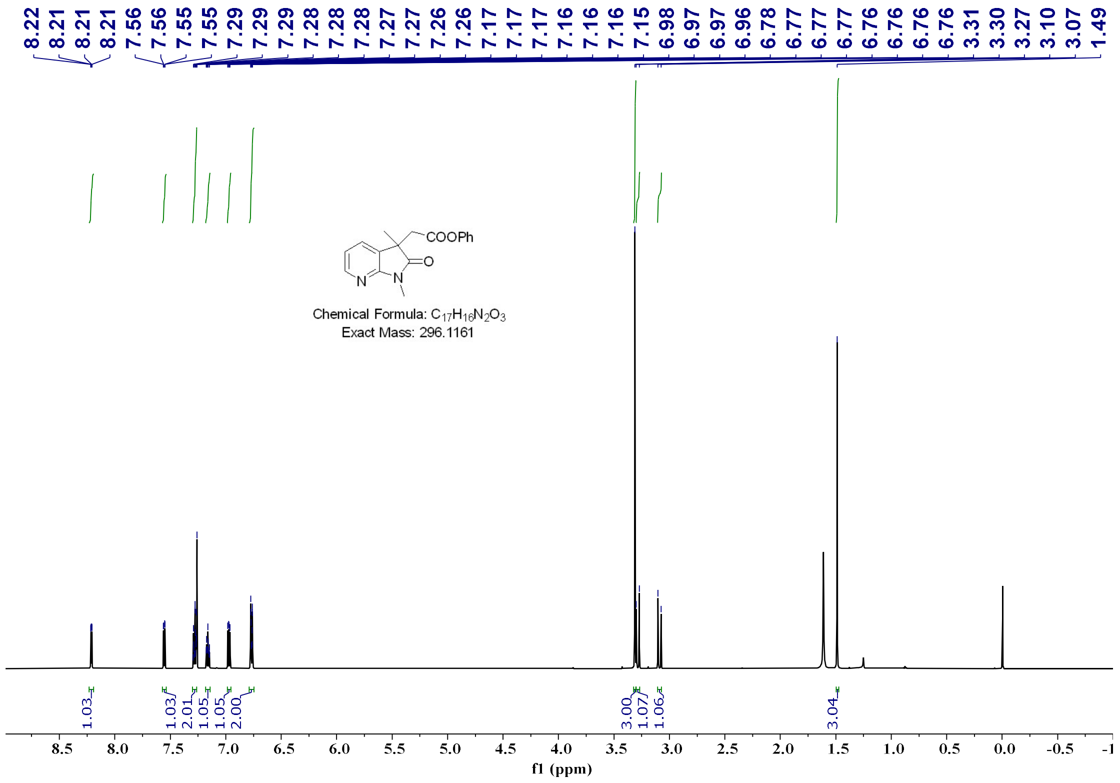

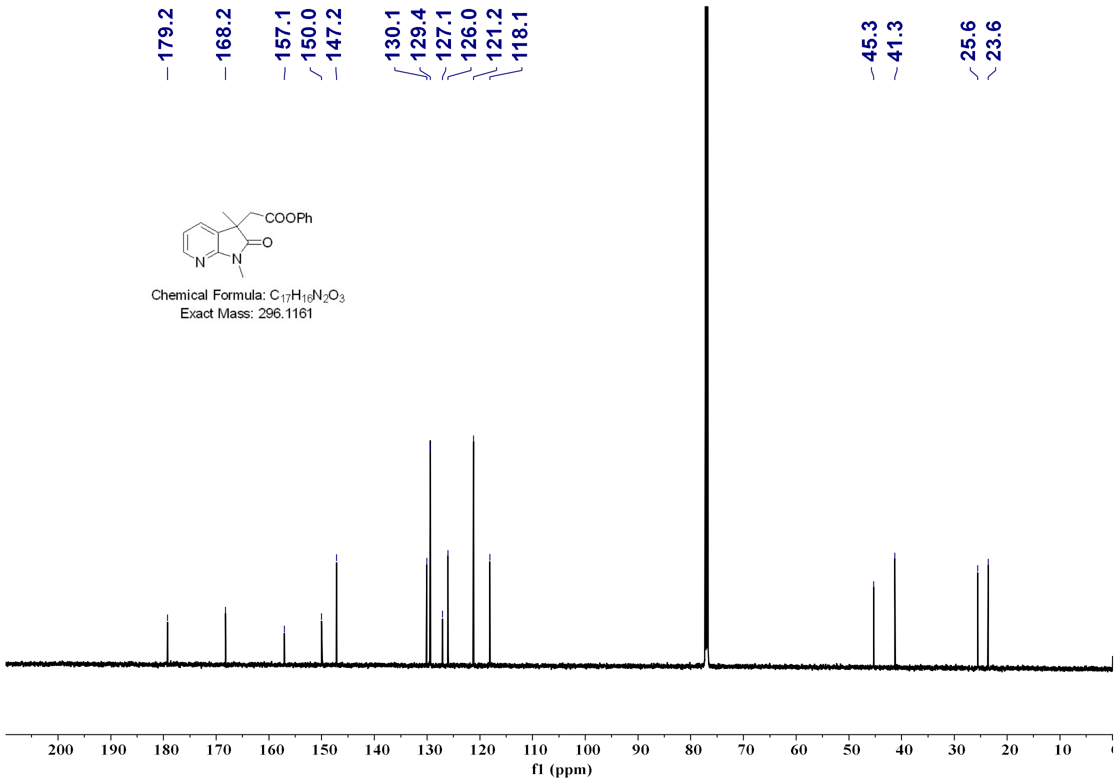

6l

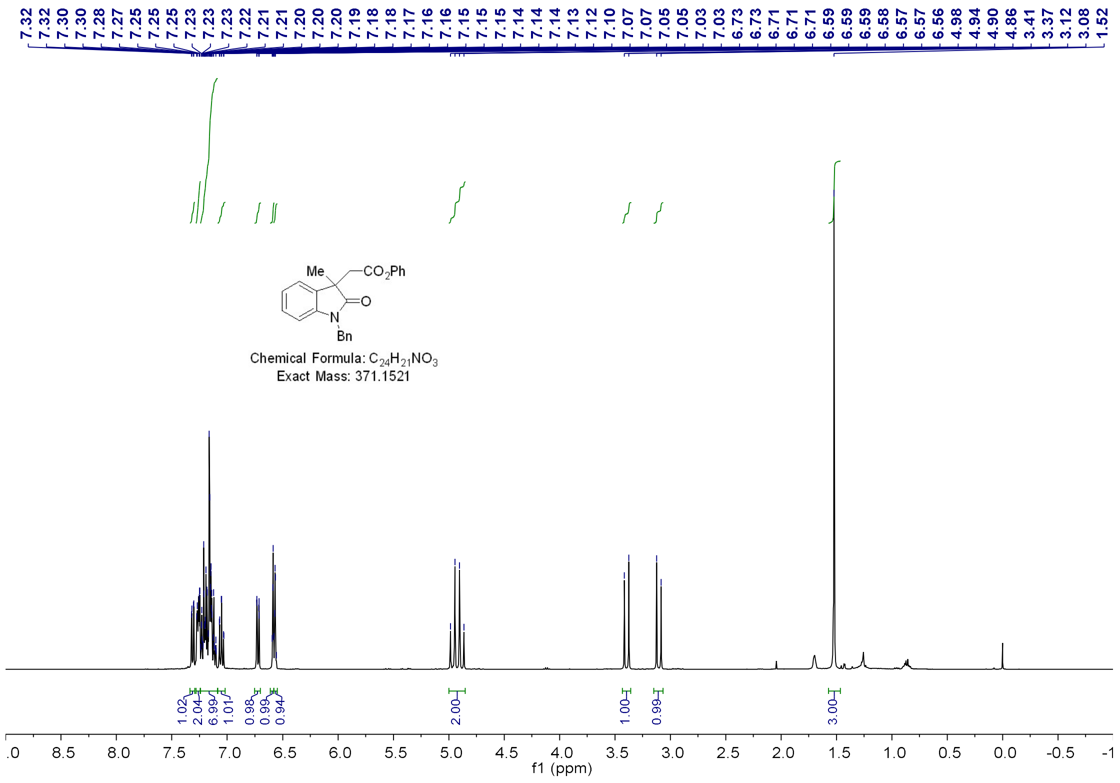

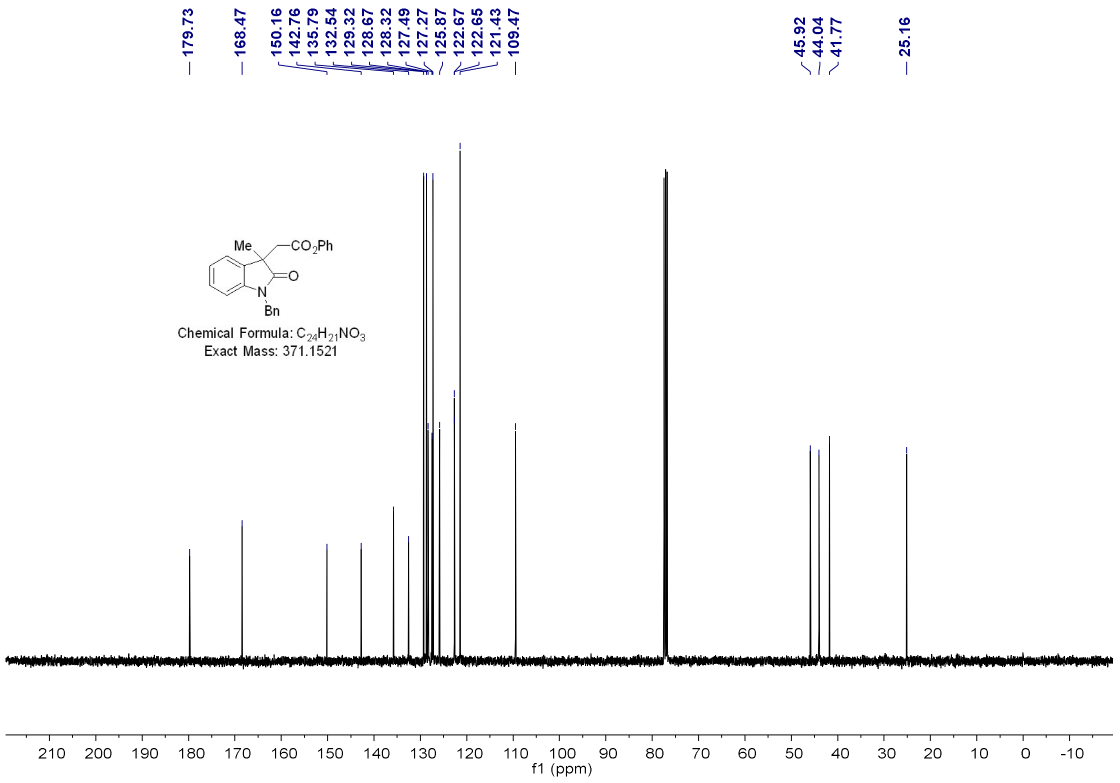

6m

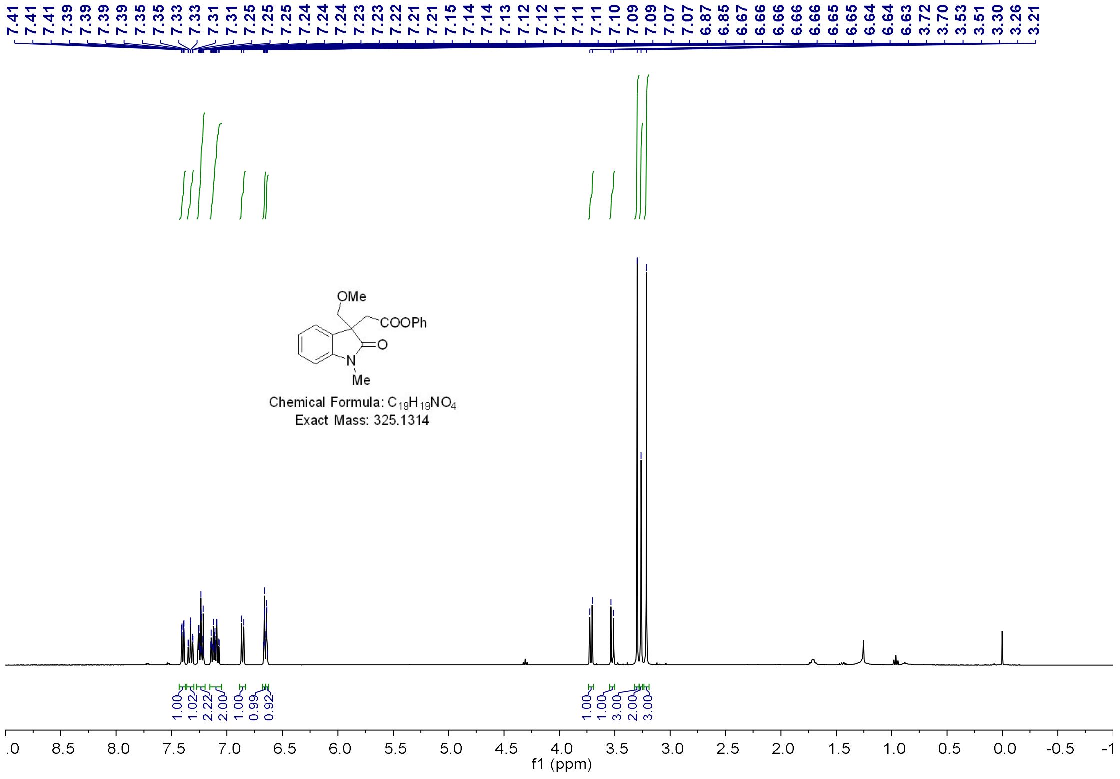

OMe
COOPh
O
N
Me
Chemical Formula: C19H19NO4
Exact Mass: 325.1314
f1 (ppm)

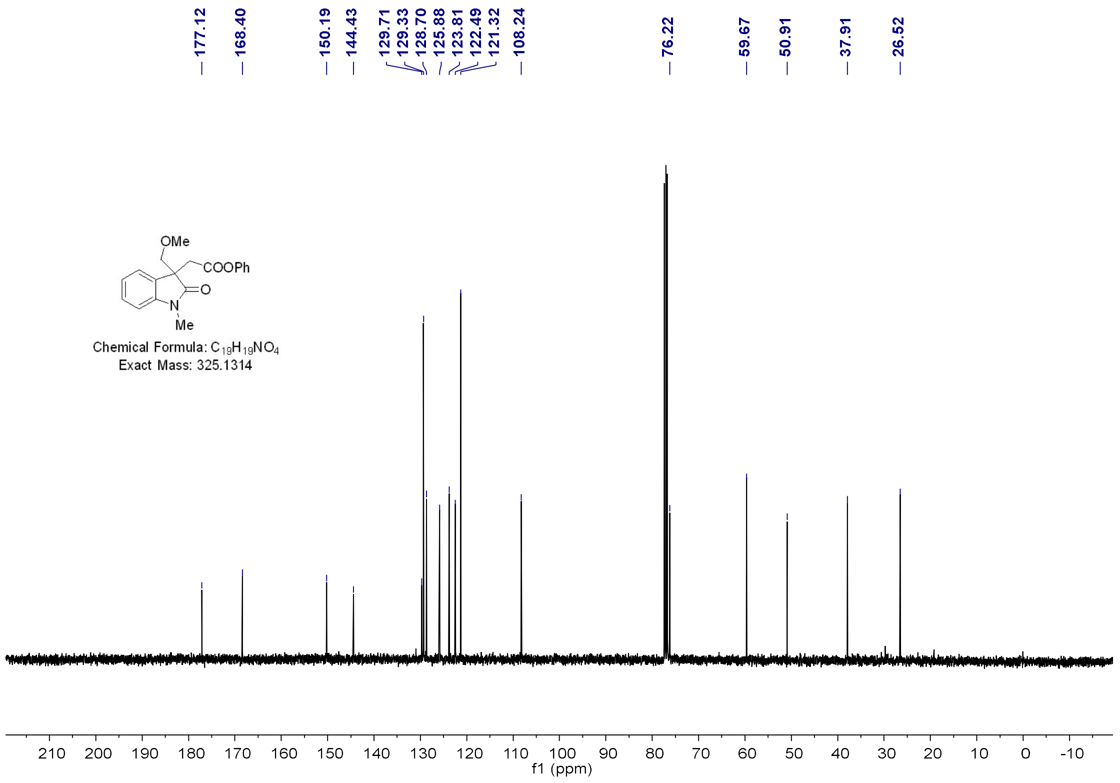

OMe
COOPh
O
N
Me
Chemical Formula: C19H19NO4
Exact Mass: 325.1314
f1 (ppm)

6n

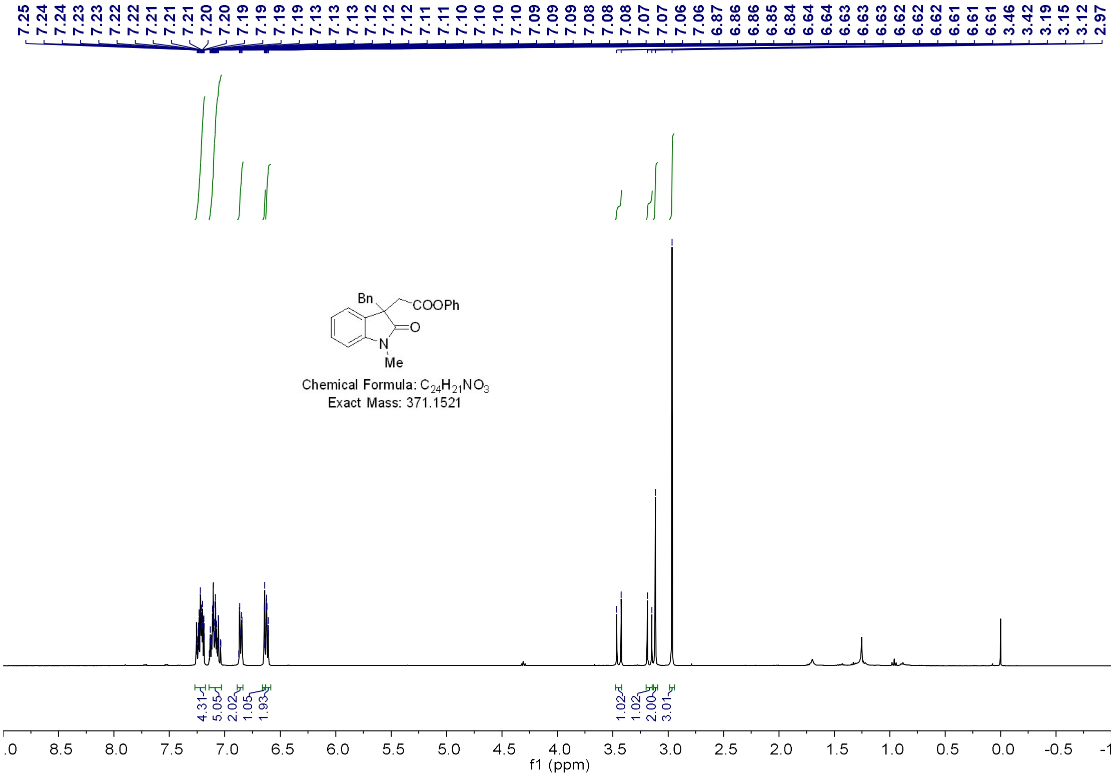
Bn
COOPh
N
Me
Chemical Formula: $C_{24}H_{21}NO_3$
Exact Mass: 371.1521
f1 (ppm)

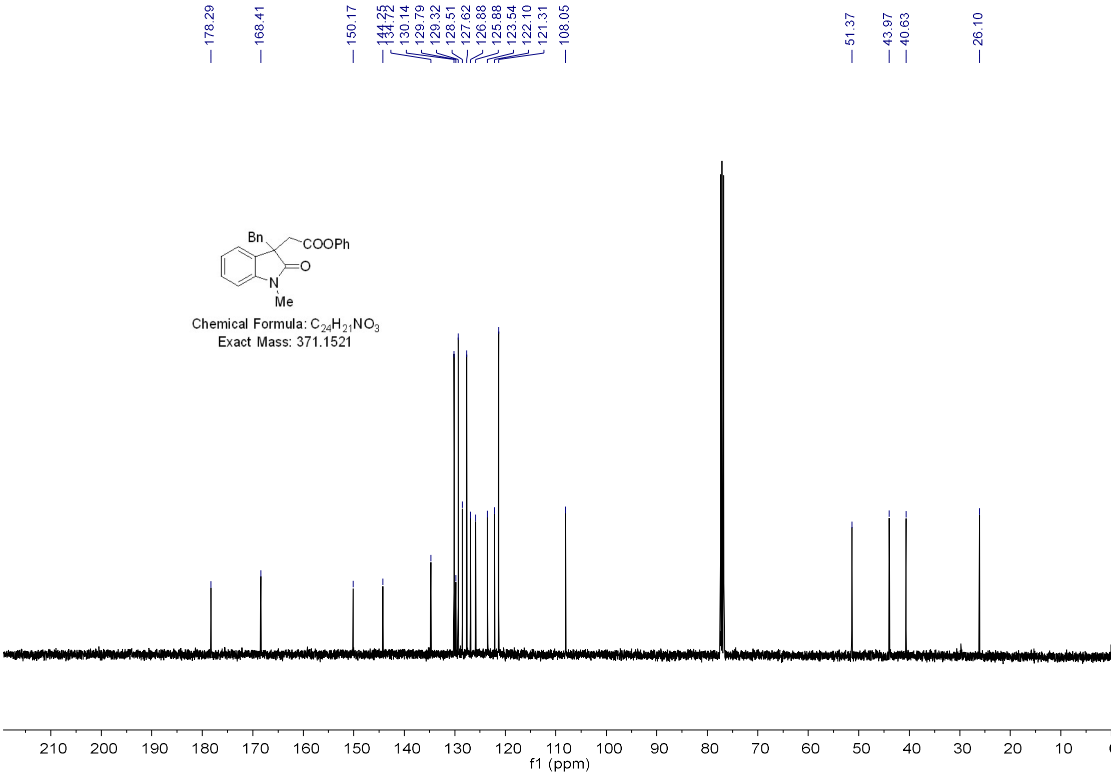
178.29
168.41
150.17
130.14
129.79
129.32
128.51
127.62
126.88
125.88
123.54
122.10
121.31
108.05
51.37
43.97
40.63
26.10
Bn
COOPh
N
Me
Chemical Formula: $C_{24}H_{21}NO_3$
Exact Mass: 371.1521
f1 (ppm)

6o

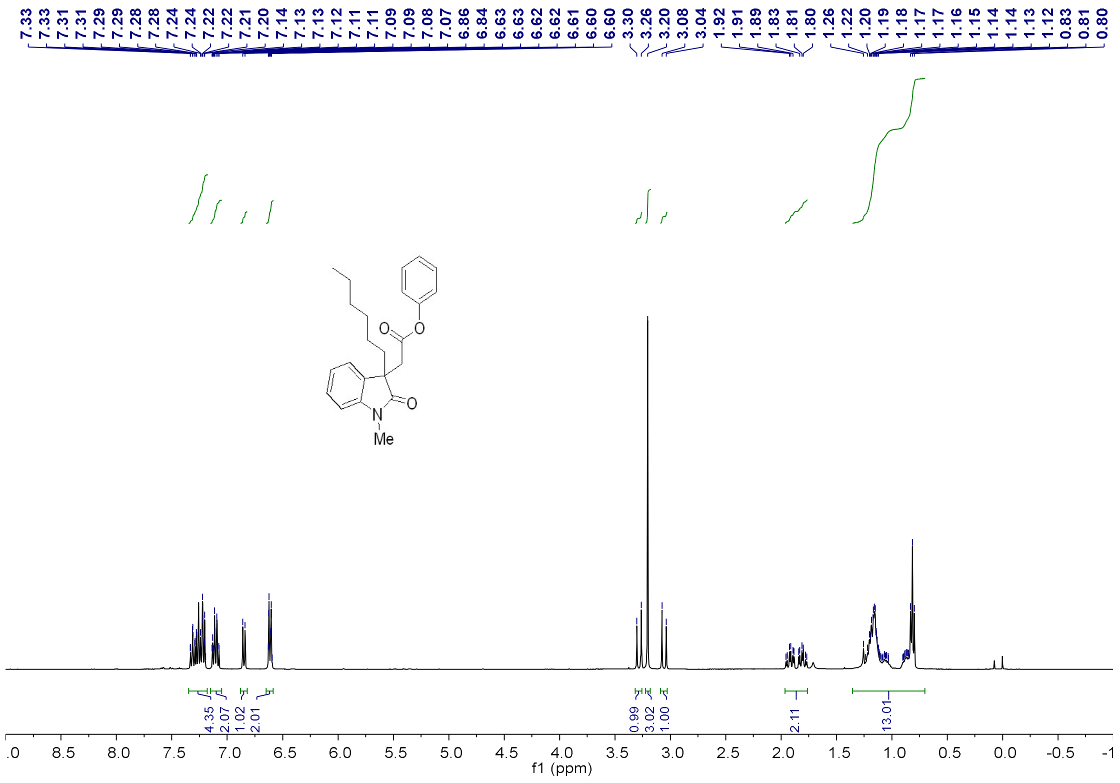

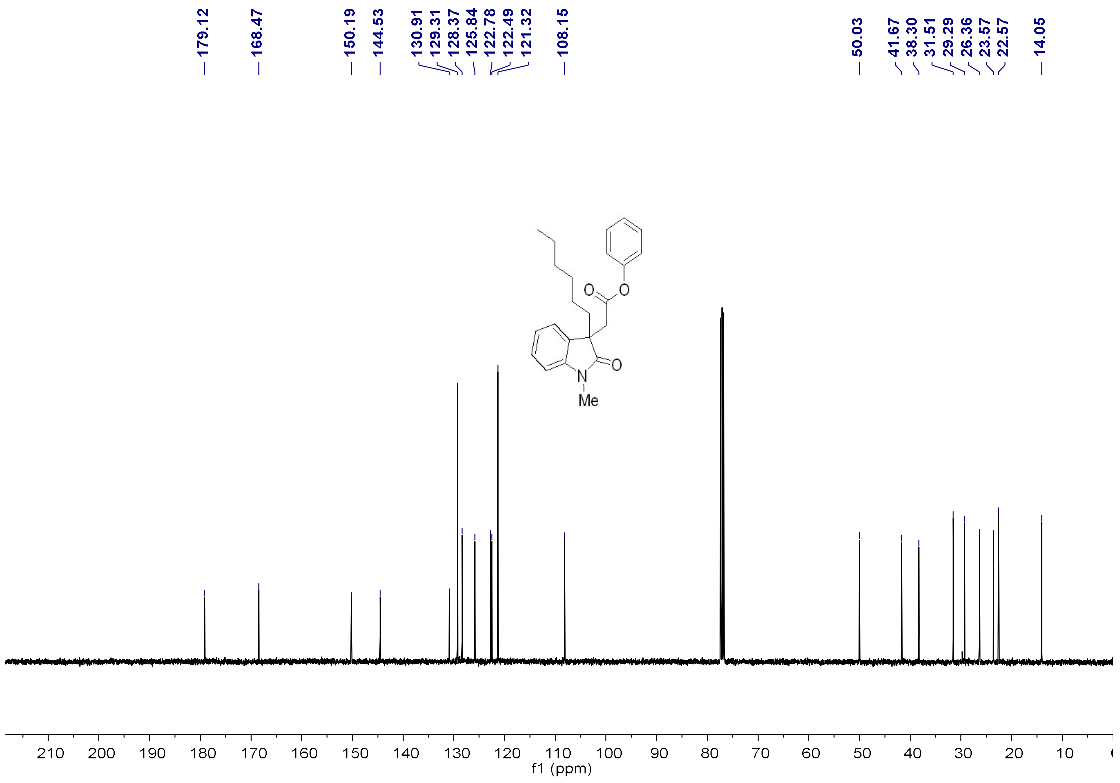

6p

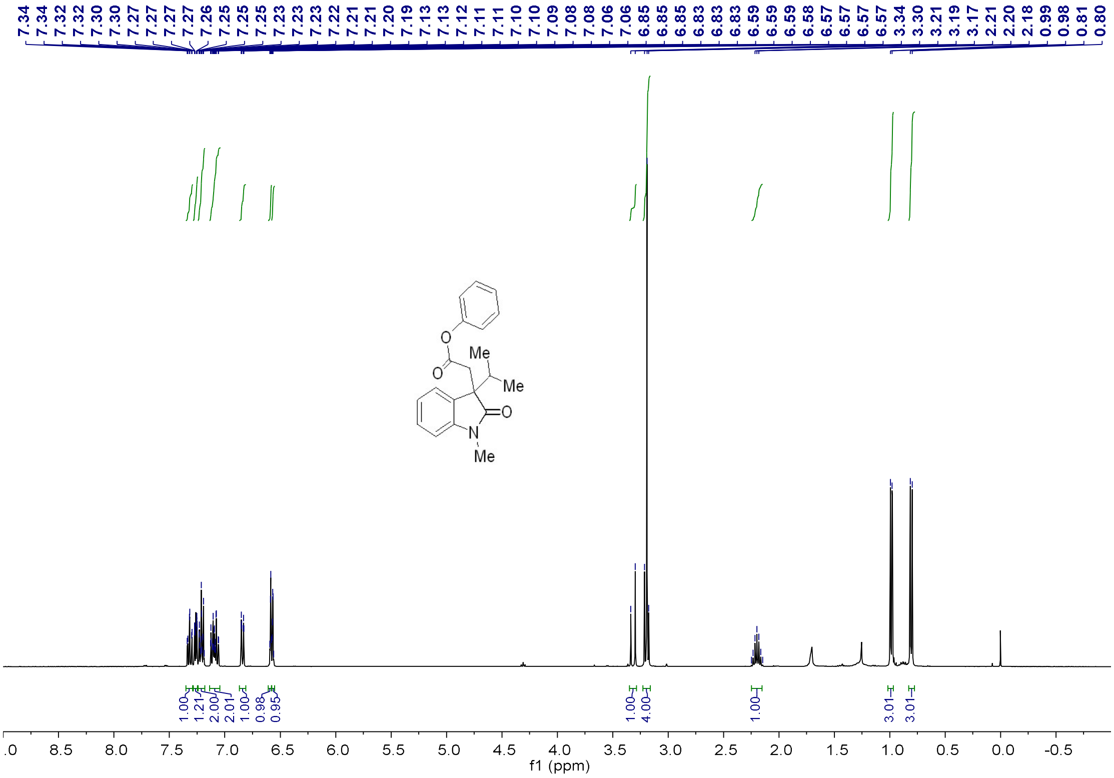
7.34
7.34
7.32
7.30
7.30
7.27
7.27
7.27
7.27
7.26
7.25
7.25
7.25
7.23
7.23
7.23
7.22
7.21
7.21
7.20
7.19
7.13
7.13
7.12
7.11
7.11
7.10
7.10
7.09
7.08
7.08
7.06
7.06
6.85
6.85
6.85
6.83
6.83
6.83
6.59
6.59
6.59
6.58
6.57
6.57
6.57
6.57
3.34
3.30
3.21
3.19
3.17
2.21
2.20
2.18
0.99
0.98
0.81
0.80
Me
Me
O
O
N
O
Me
1.00
1.21
2.00
2.01
1.00
0.98
0.95
1.00
4.00
1.00
3.01
3.01
f1 (ppm)

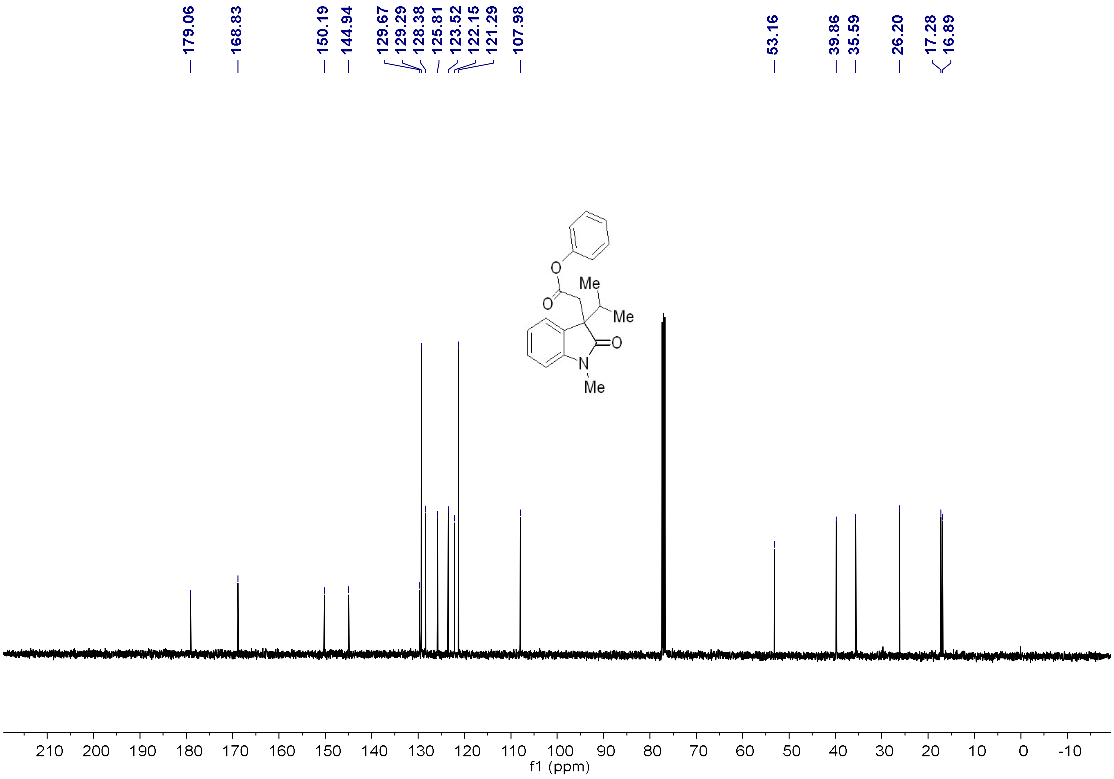
179.06
168.83
150.19
144.94
129.67
129.29
128.38
125.81
123.52
122.15
121.29
107.98
53.16
39.86
35.59
26.20
17.28
16.89
Me
Me
O
O
N
O
Me
f1 (ppm)

6q

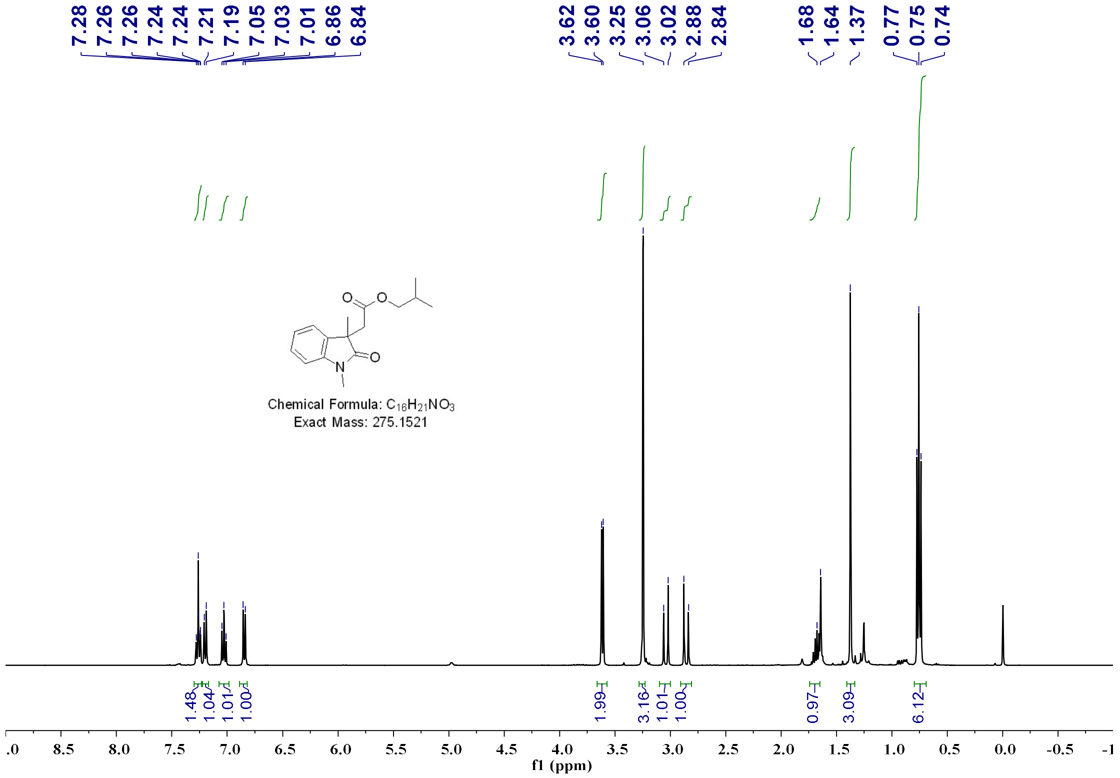
7.28
7.26
7.26
7.24
7.24
7.21
7.19
7.05
7.03
7.01
6.86
6.84
3.62
3.60
3.25
3.06
3.02
2.88
2.84
1.68
1.64
1.37
0.77
0.75
0.74
Chemical Formula: C16H21NO3
Exact Mass: 275.1521
1.48
1.04
1.01
1.00
1.99
3.16
1.01
1.00
0.97
3.09
6.12
f1 (ppm)

dzt-4-110

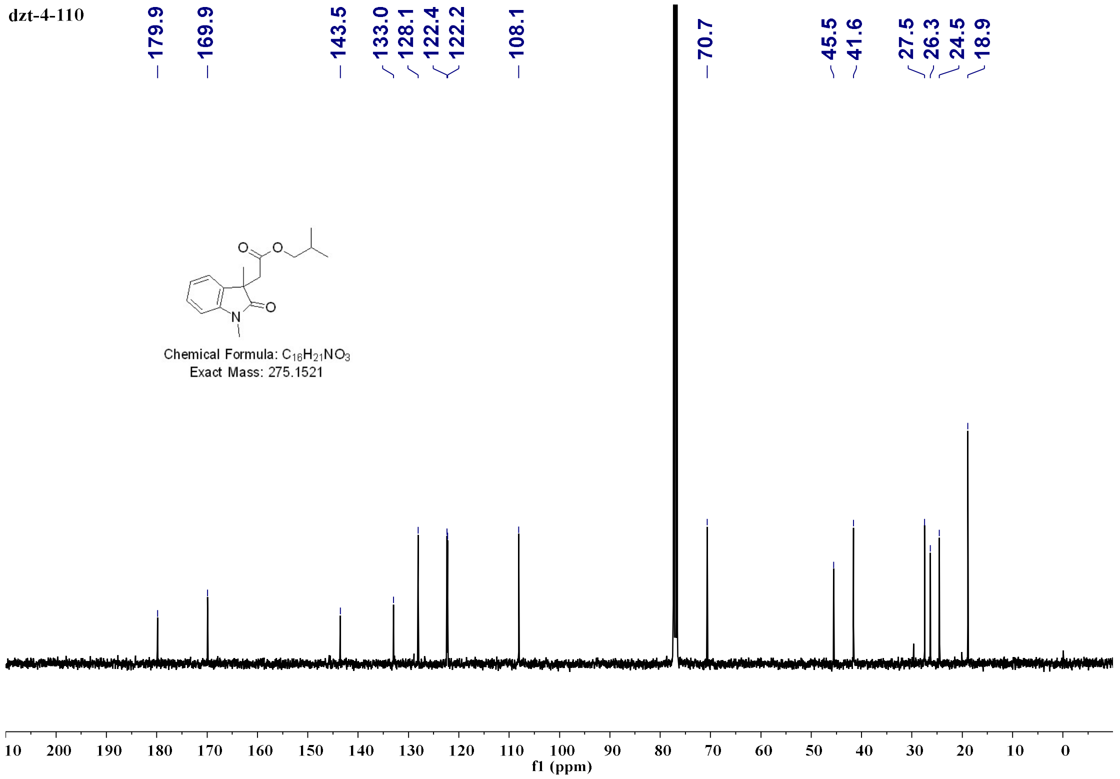
179.9
169.9
143.5
133.0
128.1
122.4
122.2
108.1
70.7
45.5
41.6
27.5
26.3
24.5
18.9
Chemical Formula: C16H21NO3
Exact Mass: 275.1521
f1 (ppm)

6r

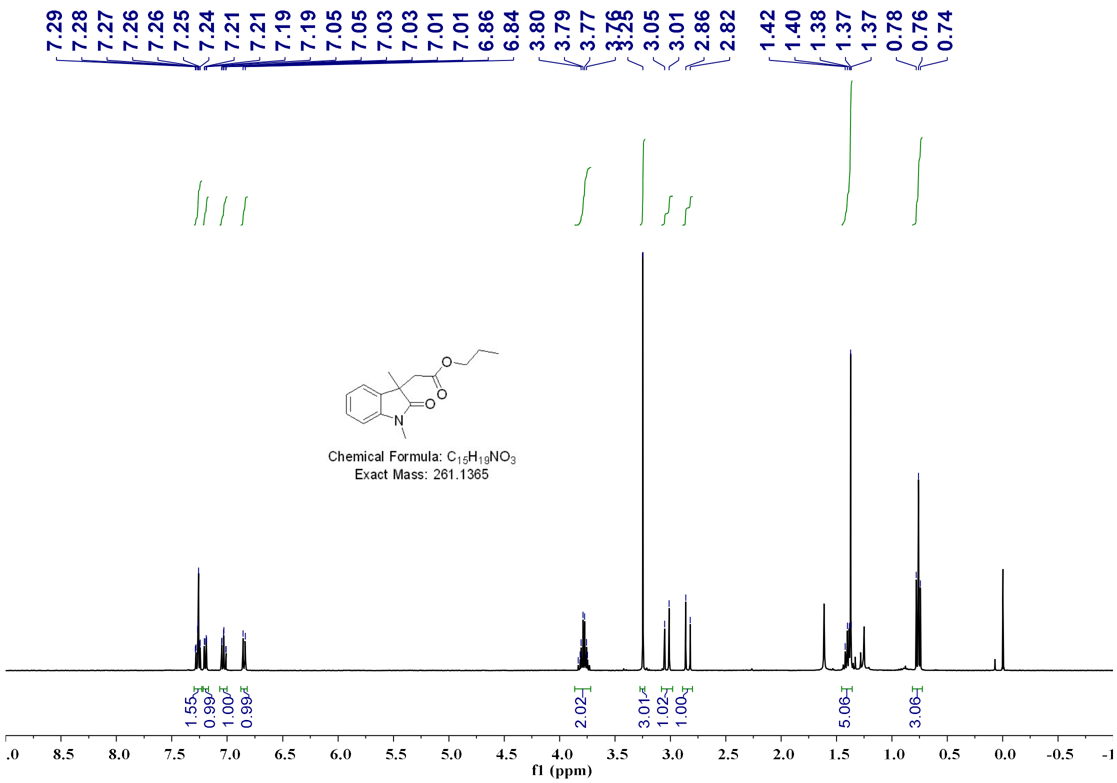
7.29 7.28 7.27 7.26 7.26 7.25 7.24 7.21 7.21 7.19 7.05 7.05 7.03 7.03 7.01 7.01 6.86 6.84 3.80 3.79 3.77 3.76 3.25 3.05 3.01 2.86 2.82 1.42 1.40 1.38 1.37 1.37 0.78 0.76 0.74
Chemical Formula: $C_{15}H_{19}NO_3$
Exact Mass: 261.1365
1.55 0.99 1.00 0.99 2.02 3.01 1.02 1.00 5.06 3.06
f1 (ppm)

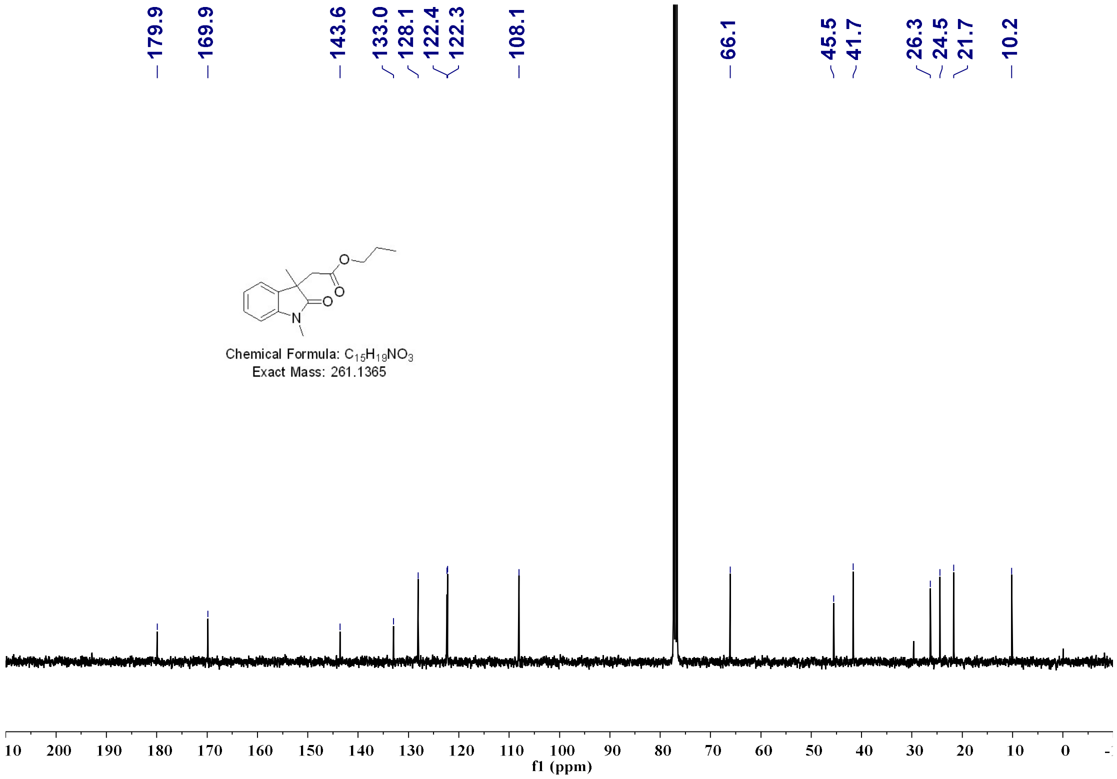
179.9 169.9 143.6 133.0 128.1 122.4 122.3 108.1 66.1 45.5 41.7 26.3 24.5 21.7 10.2
Chemical Formula: $C_{15}H_{19}NO_3$
Exact Mass: 261.1365
f1 (ppm)

6s

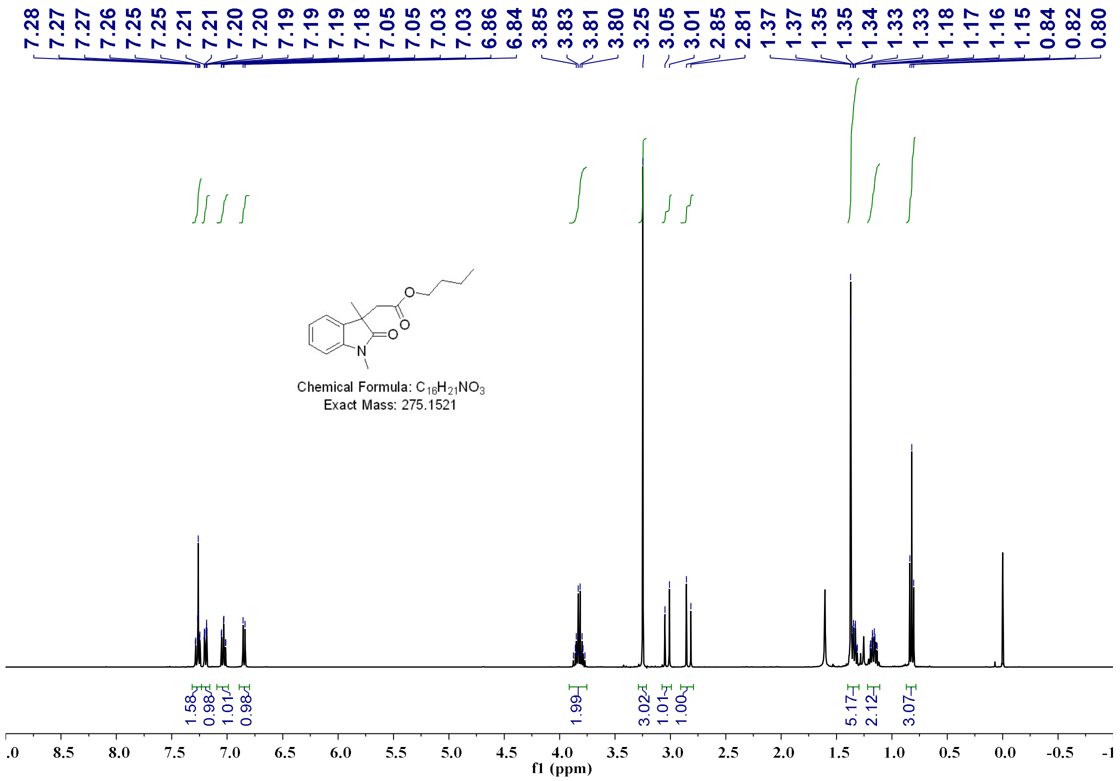
7.28 7.27 7.27 7.26 7.25 7.25 7.21 7.21 7.20 7.20 7.19 7.19 7.18 7.05 7.05 7.03 7.03 6.86 6.84 3.85 3.83 3.81 3.80 3.25 3.05 3.01 2.85 2.81 1.37 1.37 1.35 1.35 1.34 1.33 1.33 1.18 1.17 1.16 1.15 0.84 0.82 0.80
Chemical Formula: $C_{16}H_{21}NO_3$
Exact Mass: 275.1521
1.58 0.98 1.01 0.98 1.99 3.02 1.01 1.00 5.17 2.12 3.07
f1 (ppm)

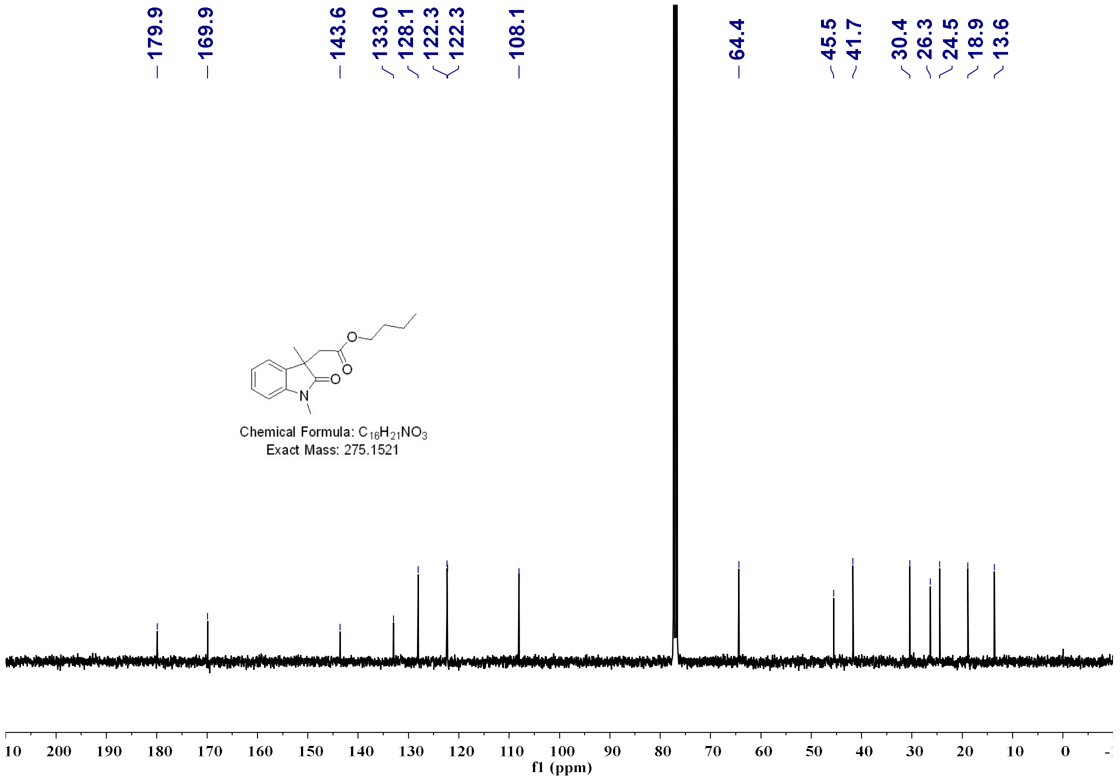
179.9 169.9 143.6 133.0 128.1 122.3 122.3 108.1 64.4 45.5 41.7 30.4 26.3 24.5 18.9 13.6
Chemical Formula: $C_{16}H_{21}NO_3$
Exact Mass: 275.1521
f1 (ppm)

7. References

[1] Wang, K.; Ding, Z.; Zhou, Z.; Kong, W. Ni-Catalyzed Enantioselective Reductive Diarylation of Activated Alkenes by Domino Cyclization/Cross-Coupling. *J. Am. Chem. Soc.* **2018**, *140*, 12364-12368.

[2] Bergonzini, G.; Cassani, C.; Lorimer-Olsson, H.; Hęrberg, J.; Wallentin, C. Visible-Light-Mediated Photocatalytic Difunctionalization of Olefins by Radical Acylarylation and Tandem Acylation/Semipinacol Rearrangement. *Chem. Eur. J.* **2016**, *22*, 3292-3295.

[3] Xu, S.; Chen, J.; Liu, D.; Bao, Y.; Liang, Y.; Xu, P. Aroyl chlorides as novel acyl radical precursors via visible-light photoredox catalysis. *Org. Chem. Front.*, **2017**, *4*, 1331-1335.

[4] Ji, W.; Tan, H. Wang, M. Lia, P.; Wang, L. Photocatalyst-free hypervalent iodine reagent catalyzed decarboxylative acylarylation of acrylamides with α-oxocarboxylic acids driven by visible-light irradiation *Chem. Commun*. **2016**, *52,* 1462-1465.

[5] Zheng, L.; Huang, H.; Yang, C.; Xia, W. UV Light-Mediated Difunctionalization of Alkenes through Aroyl Radical Addition/1,4-/1,2-Aryl Shift Cascade Reactions. *Org. Lett.* **2015**, *17*, 1034-1037.

[6] Xu, S.; Wang, k.; Kong W. Ni-Catalyzed Reductive Arylacylation of Alkenes toward Carbonyl-Containing Oxindoles. *Org. Lett.* **2019**, *21*, 7498-7503.

[7] Wang, G.; Wang, S.; Wang, J.; Chen, S.; Yu, X. Synthesis of oxindole-3-acetates through iron-catalyzed oxidative arylalkoxycarbonylation of activated alkenes. *Tetrahedron*, **2014**, *70*, 3466-3470.

[8] Wang, H.; Guo, L.; Duan, X. Silver-Catalyzed Decarboxylative Acylarylation of Acrylamides with α-Oxocarboxylic Acids in Aqueous Media. *Adv. Synth. Catal.* **2013**, *355*, 2222-2226.
